# The Orthologue of the Fruitfly Sex Behaviour Gene *Fruitless* in the Mosquito *Aedes aegypti*: Evolution of Genomic Organisation and Alternative Splicing

**DOI:** 10.1371/journal.pone.0048554

**Published:** 2013-02-13

**Authors:** Marco Salvemini, Rocco D'Amato, Valeria Petrella, Serena Aceto, Derric Nimmo, Marco Neira, Luke Alphey, Lino C. Polito, Giuseppe Saccone

**Affiliations:** 1 Department of Biological Sciences – Section of Genetics and Molecular Biology, University of Naples “Federico II”, Naples, Italy; 2 Oxitec Limited, Oxford, United Kingdom; 3 Department of Zoology, University of Oxford, Oxford, United Kingdom; New Mexico State University, United States of America

## Abstract

In Drosophila melanogaster the doublesex (dsx) and fruitless (fru) regulatory genes act at the bottom of the somatic sex determination pathway. Both are regulated via alternative splicing by an upstream female-specific TRA/TRA-2 complex, recognizing a common cis element. dsx controls somatic sexual differentiation of non-neural as well as of neural tissues. fru, on the other hand, expresses male-specific functions only in neural system where it is required to built the neural circuits underlying proper courtship behaviour. In the mosquito Aedes aegypti sex determination is different from Drosophila. The key male determiner M, which is located on one of a pair of homomorphic sex chromosomes, controls sex-specific splicing of the mosquito dsx orthologue. In this study we report the genomic organization and expression of the fru homologue in Ae. aegypti (Aeafru). We found that it is sex-specifically spliced suggesting that it is also under the control of the sex determination pathway. Comparative analyses between the Aeafru and Anopheles gambiae fru (Angfru) genomic loci revealed partial conservation of exon organization and extensive divergence of intron lengths. We find that Aeadsx and Aeafru share novel cis splicing regulatory elements conserved in the alternatively spliced regions. We propose that in Aedes aegypti sex-specific splicing of dsx and fru is most likely under the control of splicing regulatory factors which are different from TRA and TRA-2 found in other dipteran insects and discuss the potential use of fru and dsx for developing new genetic strategies in vector control.

## Introduction

The *fruitless* gene of *D. melanogaster* (*Dmfru*) encodes transcription factors, of which one has a key role in the determination of male sexual behaviour, and the others are required for multiple non sex-specific developmental functions [1], [2], [3], [4], [5], [6], [7]. Male courtship in *Drosophila* is an elaborate ritual that involves multiple sensory inputs and complex motor outputs showing largely a fixed-action pattern (male versus female orientation, tapping the female, singing by vibrating the wing, licking female genitalia and curling his abdomen for copulation) [8]. Certain *fru* loss-of-function alleles disrupt both male courtship behaviour and sexual orientation: performance of the male courtship ritual is reduced, and it is directed indiscriminately at either sex [9], [10], [11], [12], [13]. Strong *fru* alleles completely abolish male courtship behaviour, while weaker *fru* alleles can disrupt individual steps of this courtship [9], [11]. These observations suggest that *fru* is required during development to let the adult male brain execute each step of the courtship ritual, not just a single critical step. Hence sexual behaviour is apparently “hard wired” in the Drosophila CNS, leaving little plasticity, if any. However no easily detectable neuronal anatomical differences that might account for the dramatically different sexual behaviours of males and females have been found in the overall 100.000 neurons fly brain, until recently. Indeed in contrast to the preliminary conclusion that in *Drosophila* the *fruitless* circuit (2000 *fru* ^+^ neurons forming an interconnected circuit) is anatomically largely isomorphic in the two sexes [14], [15], substantial differences in wiring and gross anatomy between male and female fly brains have been recently discovered [16]. Even more interesting is the very recent finding that the *Drosophila* males can learn to distinguish (by a male pheromone lingered on mated females cuticles) and then court virgin females rather then mated ones, revealing plasticity in an innate behaviour [17]. Those olfactory neurons and mushroom bodies neurons, involved in this courtship learning, express FRU in males and use dopamine as an instructive-learning signal. This suggests that differences in neuronal anatomy of specific brain regions, might underlie the profound differences in behaviour between males and females in *Drosophila* [18], and presumably in many other species as well.

*fru* is one of the most complex genes of *Drosophila* and also one of the largest, spanning about 130 kb. All FRU isoforms contain a BTB (Broad-complex, Tramtrack and Bric-a-brac) domain, which serves as dimerization interface and a C-terminal C_2_H_2_ zinc-finger domain for the DNA binding function [19]. The FRU proteins are encoded by eighteen different transcripts which arise from four alternative non-sex-specific promoters (P1–P4) and alternative splicing at both the 5′ and 3′ ends [10], [12], [20], [21]. The *fru* functions dedicated to promote male sexual behaviour are mediated by transcripts, derived from the most distal *fru* promoter (P1), which undergo male-specific alternative splicing; female-specific *fru* transcripts appear not to encode a functional protein. The sex-specific *fru* mRNAs are detectable from 3^rd^ instar larval stage till adulthood [22]. Transcripts derived from the other known *Dmfru* promoters (P2, P3 and P4 – located between P1 and the first BTB encoding exon) are present in both sexes from embryonic stage (P3 and P4), mediating the correct development of neuronal tissues [9], [23], or from pupal stage (P2), involved in the differentiation of imaginal-disc derivatives [9], [22].

In parallel to *fru*, the *doublesex* gene controls nearly all somatic sexual differences outside the nervous system, as well as many aspects of the nervous system sexual dimorphism. Both genes act as the bottom regulators of the somatic sex determination cascade of *D. melanogaster* and they are regulated through common *cis* elements *via* alternative splicing by a complex containing the serine-arginine-rich splicing regulators *transformer* (TRA) and *transformer-2* (TRA-2) [24], [25]. Of these, TRA-2 protein is present in both males and females, but functional TRA is expressed only in females. The female-specific splicing of *fru* and *dsx* pre-mRNAs requires the binding of these proteins to TRA/TRA-2 binding sites: 13 nt *cis*-acting elements present in multiple clustered copies only in female-specific exons of both genes [26], [27]. Putative conserved TRA/TRA-2 binding sites have been identified in all female-specific exons of the known *dsx* dipteran homologs [28]. In particular, in the *Dmfru* locus, three TRA/TRA-2 binding sites are present in the *fru* female-specific exon [27], located immediately upstream (50–230 nt away) of a female-specific 5′ splice donor site, and 1.3 kb downstream the 5′ donor site of the preceding exon. Both *Dmfru* male- and female-specific 5′ donor splicing sites are canonical splicing sites [27]; however, binding of the TRA/TRA2 enhancer complex activates the female-specific 5′ splice site, while its activity is not required for the processing of the *fru* pre-mRNA in males [29], [30].

Recent insights from non-drosophilid dipteran and non-dipteran insects suggest an evolutionarily conserved role for FRU in innate sexual behaviour (for a review see [17]). In the dipteran *An. gambiae* [31] and in the hymenopteran *Nasonia vitripennis* [32] *fru* orthologues show conservation of sex-specific alternative splicing and male-specific protein expression in neural tissues. The female-specific exons of both *Angdsx* and *Angfru* genes each contain short sequences resembling the TRA/TRA-2 binding sites but showing degeneration and a lack of a consensus. The authors proposed that this observation indicates that the TRA/TRA-2 dependent mechanism of sex-specific splicing could be conserved in mosquitoes [31], [33]. In contrast a *tra* orthologue seems to be absent from both *Anopheles* and *Aedes* genomes [31], [34]. In the hymenopteran *Nasonia vitripennis* the *fru* architecture is essentially identical to *Drosophila* and the P1-transcripts undergo a conserved sex-specific splicing regulation. These findings suggest that conserved *fru* sex-specific splicing evolved prior to the split between Hymenoptera and Diptera (250–300 Myr) rather than acquired independently in both lineages [32]. In orthopteran insects, as various grasshoppers of *Chorthippus* spp. [35], the desert locust *Schistocerca gregaria* [36] and the cockroach *Blatella germanica* [37], *fru* orthologues were isolated but no sex-specific transcripts were detected by RT-PCR analysis. In spite of this, *fru* nymphal RNAi knockdown experiments revealed that in *S. gregaria* and *B. germanica fru* orthologues play important roles respectively in the regulation of successful copulation in the adult male [36] and in male sexual behaviour [37]. This suggests that the function of the *fru* gene as master regulator of male sexual behaviour has been conserved during insect evolution [36].

Due to the complex genomic organization of the *Drosophila fru* locus and the low expression level of the *fru* sex-specific transcripts, restricted in many cases to small cluster of neurons [22], [38], [39], automatic annotation usually fails to identify a complete *fru* orthologue in sequenced genomes, despite an increasing number of corresponding ESTs. This suggests that a manually curated *in silico* search followed by a molecular data validation may currently be required to unambiguously identify a complete *fru* locus.

In this work we present a detailed structural analysis of the *fru* gene in the mosquito *Aedes aegypti* (*Aeafru*), focusing on the evolution of its genomic organization and splicing regulation. *Ae. aegypti* is a major arboviral disease vector and has been studied for decades both for basic science and to develop new control methods. Despite this, very little is known about the genetic control of some significant aspects of its biology, including sex determination and reproductive behaviour, that could be important targets for the future control strategies [40], [41]. Current knowledge about sex determination in *Ae. aegypti* is restricted to the primary signal which, as observed for other mosquitoes, comprises an autosomal locus, *M*, which has a dominant male determiner, not yet molecularly identified [34], and to the evolutionarily conserved double switch gene *doublesex* (*Aeadsx*). The *Aeadsx* gene is thought to be involved in the developmental control of sex-specific somatic tissues, based on its conservation of sex-specific alternative splicing and of encoded sex-specific proteins [42]. When compared to *dsx* orthologues in other dipteran species, [28], [43], *Aeadsx* sex-specific splicing regulation seems to be more complex, suggesting the possibility of somewhat divergent regulation.

The courtship behaviour of *Ae. aegypti* has been much less studied and understood, as for many mosquito species with a swarming reproductive behaviour [44]. In this species both sexes interact acoustically by shifting their flight tones to match, resulting in a courtship duet [45]. Which are the key genetic regulators of *Aedes* promoting this complex sex-specific sexual behaviour? An *Aedes* orthologue of *Drosophila fru* would be a plausible candidate, if functionally conserved. Such conservation is likely considering that the *Anopheles gambiae fru* (*Angfru*) has a conserved sex-specific splicing regulation and that both mosquito species show a similar sexual behaviour [31]. With the aim to address this question we isolated the *Aeafru* gene and we reported the first developmental expression analysis of the *fru* gene outside drosophilids. Furthermore a sequence comparison of the female-specifically regulated exons of *Aeafru* and *Aeadsx* led us to identify new putative *cis*-acting elements, shared by both *dsx* and *fru*, potentially involved in their sex-specific alternative splicing, suggesting that a novel sex-specific upstream splicing regulator(s) has been recruited in *Ae. aegypti* during evolution.

## Results and Discussion

### Molecular characterization of the *Aeafru* gene

As observed for *Drosophila* and *An. gambiae*, *fru* seems to be a single copy gene in *Ae. aegypti*.

A putative *fru* orthologue was predicted in the supercontig 1.199 of the AaegL1.2 annotation of the *Ae. aegypti* genome sequence as the AAEL006301 gene, which however seems to be incomplete.

**Figure 1:**
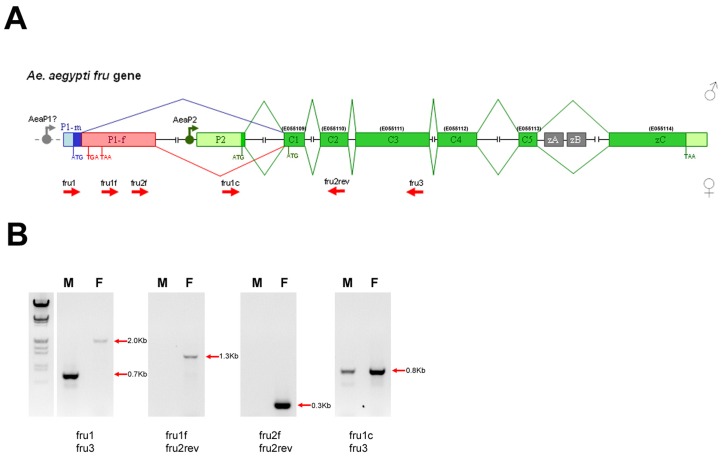
Aeafru gene structure. (A) Schematic drawing of the *fru* genomic in *Ae. aegypti* (not to scale). We renamed the five common *Aeafru* exons as C1, C2, C3, C4 and C5 and the zinc finger type C encoding exon as zC. Ensembl exon names (Ensembl id: AAEL006301) are shown in parentheses above exons. Translational start (ATG) and stop (TGA, TAA) sites are marked. The exons C1 and C2 encode the BTB domain; the exons C3, C4 and C5 encode the connecting region; the terminal exon zC encodes the type C zinc-finger domain. Exon P1 (named exon S in Demir et al., 2005) is divided in two sub-regions, a male- (P1-m in blue) and a female-specific (P1-f in pink) portion, which are alternatively spliced in a sex-specific mode. This regulation results in different 5′ encoding regions with a male-specific ATG signal in exon P1-m and multiple stop signals in exon P1-f that lead in female to the use of a non-sex-specific ATG signal in exon C1. Exon P2 is present in both sexes and encodes a short non-sex-specific N-terminal box 8 aa long. (B) RT-PCR amplifications of *Aeafru* sex-specific and common cDNA fragments on sexed adult *Ae. aegypti* mosquitoes. Primers used in the PCR amplifications are indicated as short red arrows in Fig. 1A.

Using the AAEL006301 gene prediction as our start point, we searched for additional *Aeafru* exons encoding the apparently missing portions. By combining a classical PCR-based approach with available bioinformatic and genomic tools (see Methods) we identified: 1) a sex-specifically regulated exon, named exon P1, located 420 kb upstream of the E055109 exon, encoding a putative FRU^M^ N-terminal amino acid portion; 2) a non-sex-specifically regulated exon, named exon P2, located 230 kb upstream the E055109 exon, encoding a short additional FRU N-terminal and 3) two putative alternative zinc-finger encoding exons, corresponding to zinc-finger type A and B, located between E055113 and E055114 exons, in the 3′ region of the AAEL06301 gene, in a conserved position respect to the *Dmfru* and *Angfru* genes (Figure 1A).

Using primer pairs specific for P1 and P2 exons and for the exons E055110 and E055111 of the *Aeafru* gene, RT-PCR experiments were performed on RNA samples extracted from adult sexed *Ae. aegypti* mosquitoes and both sex-specific and non-sex-specific *Aeafru* cDNA fragments were successfully amplified, confirming these predicted exons (Figure 1B). RT-PCR analysis with a forward primer located in P1 exon and a reverse primer located in P2 exon failed to produce any cDNA amplification product, suggesting that the two exons are mutually exclusive in the mRNAs (data not shown). As the corresponding homologous *Drosophila fru* P2 exon is transcribed from a different promoter from the one responsible of P1 exon transcription, we speculate that *Aedes* has a similar mechanism leading to alternative transcripts.

Three cDNA products were cloned and sequenced: a male-specific cDNA (700 bp), a female-specific cDNA (2000 bp) and a common cDNA (800 bp). Conceptual translation of these cDNA sequences and subsequent aminoacid sequence comparison with *An. gambiae* FRU isoforms confirmed their orthology. 5 and 3′ RACE RT-PCR analyses led to obtain additional *fru*-specific sequences which then were assembled with the previous ones leading to obtain three longer cDNAs named *Aeafru^P1-m-C^* (1870 bp; male-specific), *Aeafru^P1-f-C^* (3169 bp; female-specific) and *Aeafru^P2-C^* (1907 bp; common to both sexes), encoding the *Ae. aegypti* FRU^MC^ (601 aa), FRU^C^ (552 aa) and FRU^P2-C^ (560 aa) isoforms respectively. Interestingly, as in *Drosophila* and in *Anopheles*, in *Aedes* the P1-f female-specific exon introduces a stop codon interrupting the ORF which starts in the P1-m exon, and suggesting that, as in *Drosophila*, no full length FRU is expressed in females from this promoter. The alignment of the three *Aeafru* cDNA sequences and of the *in silico* identified alternative zinc finger encoding exons (zA and zB) with the AAEL006301 genomic sequence led us to define an updated *Aeafru* genomic organization represented in Figure 1A.

**Figure 2:**
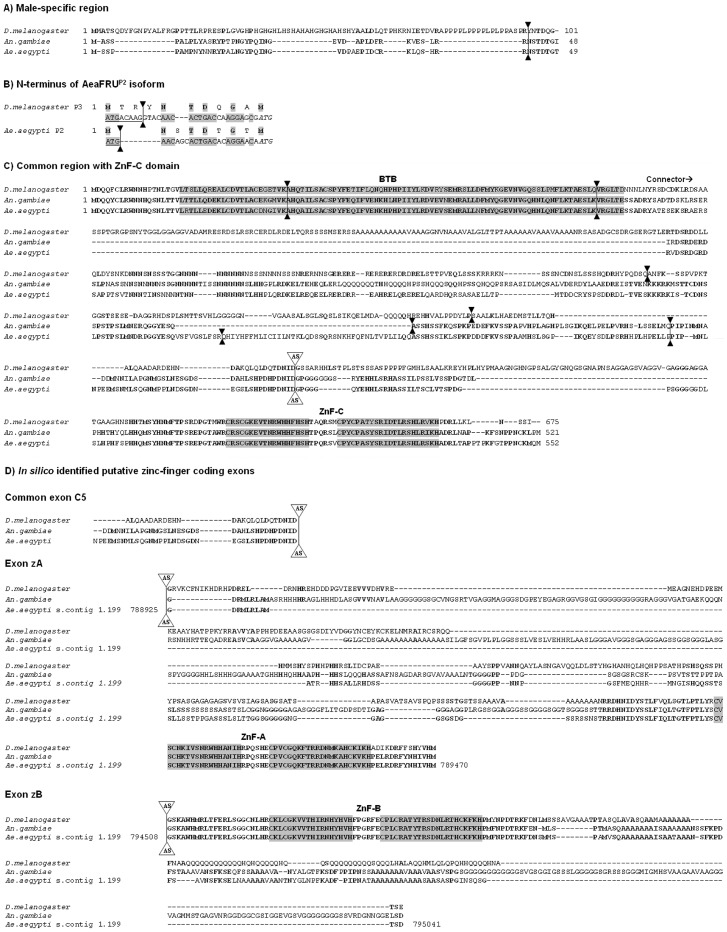
Multiples sequence alignment of the FRU isoforms. Protein sequence alignment of the *fru* isoforms of *D. melanogaster*, *An. gambiae* and *Ae. aegypti*. The conserved BTB domain and zinc finger domains are boxed in grey. Bold letters indicate amino acid identity among *Drosophila*, *Anopheles* and *Aedes* or between two of them. Intron positions are indicated by solid triangles and position of 3′ alternative splicing site is indicated by AS triangles. Gaps were introduced in the alignments to maximize similarity. The sequences are divided into: (A) a male-specific N-terminal portion encoded by AeaP1 transcripts; (B) an alternative common N-terminal portion encoded by AeaP2 transcripts (the N-terminal extension of the *Aedes* FRU^P2-C^ isoform is similar to the *Drosophila* FRU isoforms encoded by transcripts derived from the P3 promoter; in these *Dmfru* transcripts an ATG signal, located upstream the ATG present in the exon C1, leads to the in frame insertion of a short conserved amino acid box in both species); (C) a portion, common to males and females, including the BTB domain, the connector region and the zinc finger type C domain; (D) putative *in silico* identified zinc-finger type A and B domains of *Ae. aegypti* aligned with the homologous domains of *D. melanogaster* and *An. gambiae*.

The comparison of the putative AeaFRU isoforms with the FRU isoforms of *D. melanogaster* and *An. gambiae* is presented in Figure 2. The alignments revealed, as expected, high conservation of the BTB and ZnF-C domains between the three species, but very low similarity between the connector and male-specific N-terminal domains. Higher conservation of the male-specific N-terminal extension domain (65% identity) and of the connector region (43% identity) is observed comparing the sequences of the two mosquitoes only.

### Evolution of the *Aeafru* genomic organization

**Figure 3:**
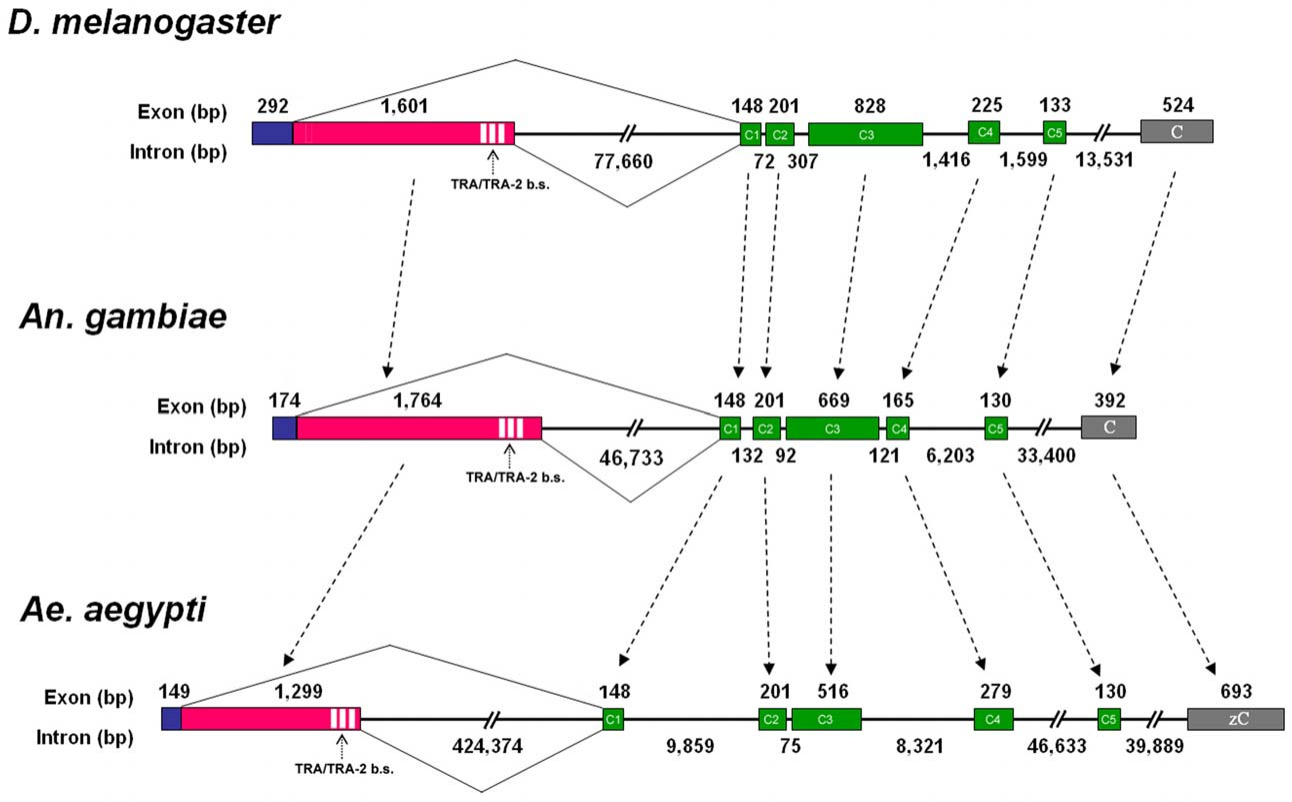
Comparative scheme of *D. melanogaster*, *Ae. aegypti* and *An. gambiae fru*-P1 genomic structure. Due to its complex structure, with multiple promoters and 5′ and 3′ alternative splicing, we compare the homologous portion of *fru* genes, starting with the sex-specific regulated region and ending with the ZnF-C domain encoding exon. *fru*-P1 common (but encoding the male-specific N-terminus) and female-specific exons are represented as blue boxes and pink boxes, respectively. Green boxes represent the non-sex-specific exons encoding BTB domain and connector region of FRU proteins while terminal grey boxes represent the ZnF-C domain encoding exons. White rectangles represent TRA/TRA-2 binding sites. The *Drosophila fru* corresponding region spans 98 Kb and is organized in 7 exons and 6 introns, with 6 common exons, preceded by the sex-specific regulated region with a male-specific and a female-specific exons. *Dmfru^MC^* translation initiates at the ATG within exon P1-m and terminates within the ZnF-C encoding exon C, while in the case of *Dmfru^C^* translation initiates at the ATG within the BTB encoding exon C1 and terminates within at the same stop signal in exon ZnF-C.

The *Aeafru* gene is contained within a large genomic region of 533 kb, significantly larger then the *Drosophila* 130 kb-long *fru*. To analyse the evolution of the *fru* genomic organization we compared the gene structure in *Drosophila*, *Anopheles* and *Aedes* (Figure 3).

*Aeafru* consists of eight exons and seven introns that vary markedly in length; to date, *Aeafru* intron 4 (424374 bp) is the largest intron reported in *Ae. aegypti* [34] and its sequence analysis reveals a high frequency of repetitive elements of various nature (constituting about 40% of whole intron sequence). The five non-sex-specific *Aeafru* exons (C1-C2-C3-C4-C5) have a corresponding similarity to the 5 non-sex-specific exons of *Drosophila* and *Anopheles fru* genes. The highest conservation is observed for exon C1 and C2, whose encoded amino acid domains are essentially the same in all three species; exon C3, C4 and C5 exhibit a more variable size and amino acid content of the encoded domain. Exon zC (zinc-finger encoding exon) is highly conserved respect both *Drosophila* and *Anopheles* species. Finally, exon P1 exhibits a conserved male-specific encoded N-terminal domain and a sex-specific alternative splicing regulation, as observed in the *Drosophila* and *Anopheles* orthologues. An extensive divergence in intron length of the *Aeafru* gene was observed respect to *Drosophila* and *Anopheles* (Figure 3). The *fru* gene of *An. gambiae* is contained within a 90 kb genomic region; the *Ae. aegypti* genomic sequence is ∼5.8-fold larger. This difference is due to the presence of very large introns in the *Ae. aegypti* homologue, with an average intron size of 88 kb, in contrast to the observed average intron size for *Angfru* (16 kb). This is consistent with the overall difference in the genome size of the two species; ∼243 Mbp for *An. gambiae* and ∼1.31 Gbp for *Ae. aegypti*. This difference is mostly due to the high frequency of repetitive sequences which constitute about 50% of the *Ae. aegypti* genome [3]. We searched for repetitive elements within *Aeafru* introns using the CENSOR software [46] and we observed the presence of multiple copies of a wide range of elements in all introns, except for the short intron 3 (75 bp long) (Figure S1, Table S1 and Table S2).

To assess the degree of genomic microsynteny between the *fru* containing region of *Ae. aegypti* (supercontig 1.199–1,9 Mb long) and *An. gambiae* (X chromosome – from 1,23 Mb to 1,48 Mb) and to analyse its nature, we compared the virtual amino acid sequences encoded by all the putative genes present in both regions, identifying and locating on genomic positions the respective putative orthologues in the two species. This analysis reveals a complex situation with substantial absence of synteny between the two *fru*-containing regions (Figure S2).

### Phylogenetic relationship and molecular evolution of the *Aeafru* gene

**Figure 4:**
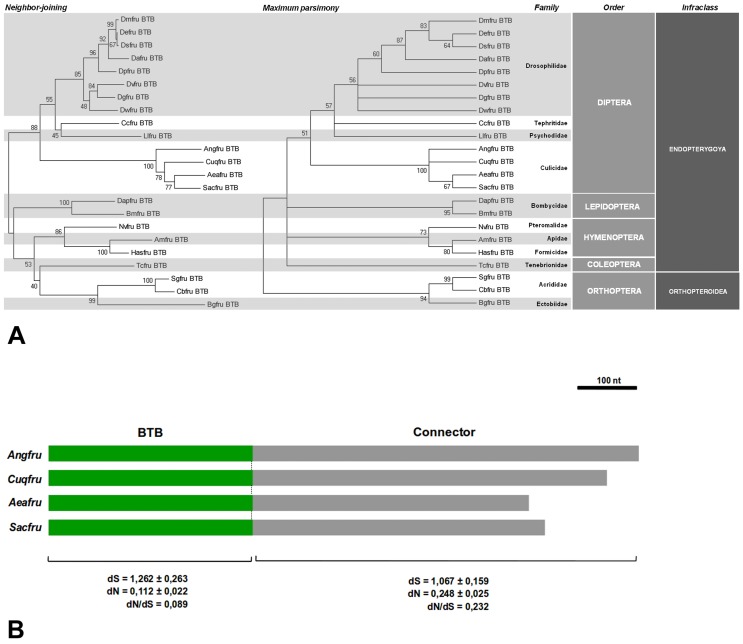
Phylogenetic and molecular evolution analyses. (A) NJ and MP consensus trees based on nucleotide alignment of the BTB encoding region of the *fru* gene of different insect species. (B) Diagram showing the BTB and the connector domain of the *fru* gene in four mosquito species. The dN, dS and dN/dS (ω) values for each domain are reported below the scheme.

To determine the phylogenetic position of the *Aeafru* gene we aligned its nucleotide sequence encoding the BTB domain to the corresponding region of the *fru* orthologues of 22 insect species (Figure 4). We included in this analysis the *fru* sequence of the arboreal mosquito species *Sabethes cyaneus* (*Sacfru*), that we have recently isolated. Males of the *Sa. cyaneus* exhibit a complex stereotyped courtship behaviour [47]; this feature makes *Sa. cyaneus* a very interesting species for future studies of courtship behaviour evolution and functional RNAi mediated knockdown assays in mosquitoes.

The mean evolutionary divergence estimated over the aligned sites (excluding gaps, 342 nucleotide positions on 348 total sites) is 0.38±0.055. The Neighbour-Joining (NJ) and Maximum Parsimony (MP) bootstrap consensus trees are shown in Figure 4A. The topology of both trees reveals a general agreement between the gene genealogy of the BTB domain encoding region and the insect phylogeny.

In particular, *Aeafru* and *Sacfru* BTB constitute a highly statistically significant cluster (100% bootstrap percentage) together with the other mosquito sequences (*Culex quinquefasciatus* and *An. gambiae*.

Subsequently, nucleotide sequences encoding the BTB and the connector domain of *Ae. aegypti* and *Sa. cyaneus* were compared to the corresponding region of the two *fru* genes of mosquitoes available in GenBank: *An. gambiae* and *Cu. quinquefasciatus*. The mean evolutionary divergence estimated over the aligned sites encoding the mosquito BTB and connector domains (excluding gaps, 345 nucleotide positions on 348 total sites for the BTB domain and 462 nucleotide positions on 765 total sites for the connector domain) are 0.283±0.03 and 0.402±0.03, respectively. Mean non-synonymous (dN) and synonymous (dS) substitution rates and their ratios (ω) were calculated among the nucleotide sequences of the mosquito *fru* orthologues available, partitioned into BTB and connector domain (Figure 4B). Both regions are subjected to strong purifying selection, with a relaxation of selective constraints in the connector region revealed by the ω value of the connector domain (0.232) significantly higher than that observed in the BTB domain (0.089).

### Developmental expression analysis of the *Aeafru* gene

**Figure 5:**
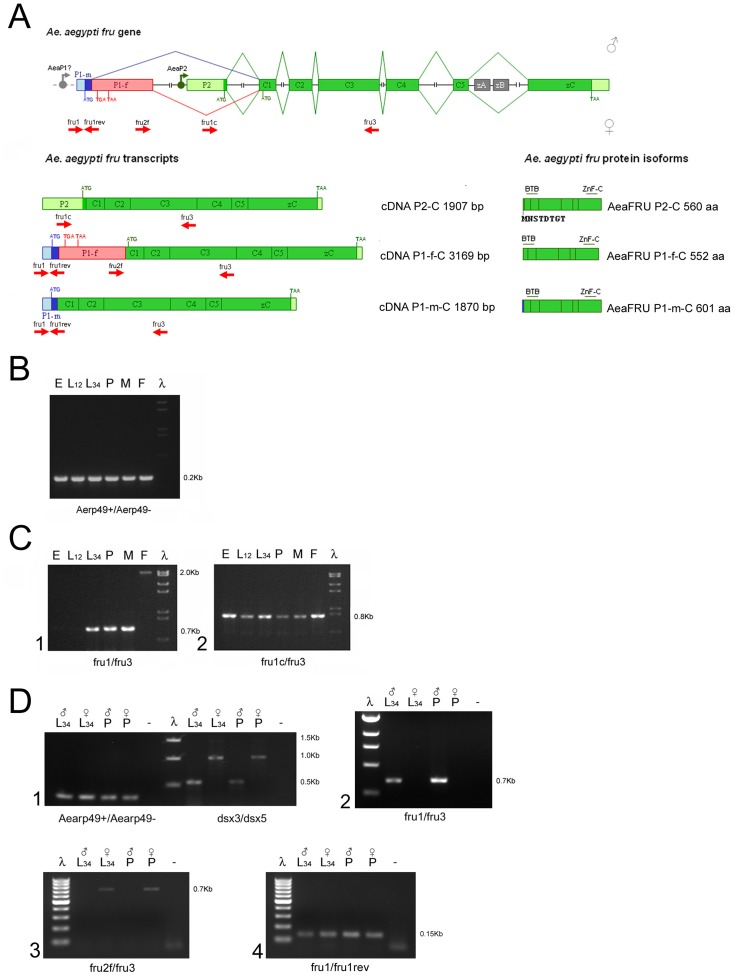
Developmental expression analysis of the *Aeafru* gene. (A) *Aeafru* gene, transcripts and protein isoforms. Transcripts derived from a putative AeaP1 promoter consist of seven exons with six introns and are alternatively spliced. The transcripts derived from promoter AaeP2 consist of seven exons and six introns and share with AaeP1 ones the common exons C1-C2-C3-C4-C5 and the zinc-finger exon zC but have the upstream exon P2 with and an alternative ATG signal which use led to the translation of AeaFRU^P2-C^ isoform. The translation of all isolated *Aeafru*-C isoforms terminates in both sexes at the stop codon (TAA) in the zinc-finger exon zC. Primers used in the following amplifications are indicated as short red arrows. (B) *Aedes aegypti* ribosomal gene *rp49* positive control. (C) *Aeafru* P1 and P2 developmental expression patterns. (D) *Aeafru* expression pattern on single sexed larval samples. Sexing of samples was performed using *Aeadsx* primer pair described in [42], which produces a unique amplification signal of 0.5 Kb in the male sample and two amplification signals, of 1.5 and 1.0 Kb, in the female sample. These signals correspond to *Aeadsx* gene sex-specifically spliced transcripts. E = 0–36 h old embryos; L12 =  early larvae; L34 =  late larvae; P =  pupae; M =  adult males; F =  adult female. All samples are composed of mixed sexes except for larvae and pupae samples of panel C, which are constitute of single sexed late larvae or pupae.

*fru* does not influence a behaviour as it happens, but rather acts during development to create the potential for a behaviour [9]. To analyze the developmental expression pattern of the *Aeafru* gene, we performed an RT-PCR analysis on total RNA extracted from different stages, from embryonic till adulthood, using primer pairs spanning the P1 sex-specifically regulated exon or the P2 exon and the common region of the gene (Figure 5). We used the *rp49* gene, constitutively expressed in *Ae. aegypti* [42], as endogenous positive control (Figure 5B). This analysis confirmed the existence in *Ae. aegypti* of two classes of transcripts with two different developmental expression patterns.

The first class of transcripts (presumably derived from an *Ae. aegypti* promoter which could correspond to the *fru*-P1 promoter of *D. melanogaster*) amplified with fru1/fru3 primers, are detected from 3^rd^ instar larval stage till adulthood, as reported for *Drosophila fru*-P1 transcripts. These transcripts are alternatively spliced in a sex-specific manner, leading to the production of the *Aeafru^P1-m-C^* and *Aeafru^P1-f-C^* mRNAs (Fig. 5C.1). RT-PCR analyses on single 3^rd^–4^th^ instar larvae and pupae, sexed using *dsx* sex-specific splicing (Figures 5D.1), detected the male-specifically spliced AeaP1 *fru* transcript (using fru1/fru3 primers; Fig. 5D.2) as well as the female-specifically spliced one (using fru2f/fru3 primers; Fig. 5D.3). These sex-specific *fru* transcripts share a common 5′ exonic region, as shown by the RT-PCR with fru1/fru1rev primers, indicating that the P1 promoter is active in both sexes (Fig. 5D.4).

At adult stage female-specific amplification product was observed at lower level respect to male-specific ones in our non quantitative conditions; however, this result is consistent with a recent microarray study in which a probe located in the common region (exon C4) of the *Aeafru* gene detected a 3-fold expression in adult males respect to females [48]. These data suggest that *fru* female-specific transcripts may be turned over more rapidly in *Ae. aegypti* at larval, pupal and adult stages.

In *Drosophila*, even if male- and female-specific *fru*-P1 transcripts are present at a similar level in both the male and female central nervous system (CNS), FRU protein is not detected in the female. This suggested that the female-specific transcripts are not translated, and also indicates that the presence/absence of the FRU protein, rather than sex-specific structural differences, is responsible for the sexually dimorphic actions of the *fru* gene in the CNS of *D. melanogaster* flies [21], [49].

The second class of *Aeafru* transcripts (amplified with fru1c/fru3 primers and corresponding to the *Aeafru^P2-C^* mRNA) is non-sex-specific and, as observed in *Drosophila*, possiblyderived from a different promoter respect to P1 promoter) that seems to be active from embryonic stage till adulthood (Fig. 5C.2). The non-sex-specific transcript was also detected in sexed larvae and pupae samples (data not shown). The P3 and P4 promoters of *D. melanogaster fru* gene, also exhibit a similar constitutive transcriptional activity [9], [23].

### *In silico* analysis of the *Aeafru* splicing sites

*Aeafru* P1 transcripts undergo sex-specific alternative splicing from late larval stage till adulthood. An *in silico* analysis of the 5′ donor/3′ acceptor splicing sites (5′ss/3′ss) at *Aeafru* exon/intron junctions was performed to find suboptimal sites, using the 5′ss consensus (MAG/GTRAGT) and 3′ss consensus (Y_n_NYAG/G; n = 8,02+/−2,15) as in [42]. The results are reported in Figure S3–A.

This analysis revealed that the *Aeafru* gene, as the *Drosophila* orthologue, exhibits two canonical 5′ss, although used *in vivo* as alternative sex-specific [27]. In contrast, in *Anopheles* the female-specific 5′ ss of the sex-specifically regulated *fru* exon is a suboptimal splicing site (Figure S3–B).

To score the intrinsic strength (independently from additional flanking signals) of the two sex-specific alternative 5′ donor splice sites, the male-specific P1-m and the female-specific P1-f, as well as of the corresponding common 3′ ss acceptor of exon C1, in the *Aaefru* gene, the MaxEntScan algorithm was applied [50]. This program is based on an approach for modelling the sequences of short motifs such as those involved in RNA splicing which accounts for non-adjacent as well as adjacent dependencies between positions. Although MaxEntScan scores are derived from human splice sites, this approach was recently used successfully to predict the *D. melanogaster* splice site strength according with the observation that *Drosophila* splice-site motifs are highly similar to human, and many spliceosomal components involved in splice-site recognition are highly conserved [51], [52], [53]. Results are reported in Figure S3–B.

This analysis confirmed the previously described results and revealed that *Aeafru* P1 female-specific splicing site (MaxEntScan score = 10.65), as well as the *Drosophila* female-specific 5′ss (MaxEntScan score = 11.37), are significantly stronger than the respective alternative male-specific 5′ss (*Dm* MaxEntScan score = 8.89; *Aea* MaxEntScan score = 9.79). Surprisingly also in *Drosophila* the *fru* female-specific 5′ss, which requires TRA/TRA-2 activation, is predicted to be stronger (MaxEntScan score = 11.37), then the male-specific one, which is used by default (*Dm* MaxEntScan score = 8.89). Most likely also the genomic contest including the flanking intronic sequences contribute to define the relative strength of these splice sites. Hence it is unclear from these data in which of the two sexes the splicing regulation requires additional sex-specific upstream factors.

In order to further investigate how alternative splicing used for *fru* is controlled in *Aedes* males and females, we searched for known *cis* splicing elements as well as for novel ones.

### Known splicing regulatory *cis*-elements of the *Aeafru* and *Aeadsx* genes

In other dipteran species the *fru* and *dsx* genes share common upstream regulators. In *Drosophila* the TRA/TRA-2 binding sites (13 nt long) [54], the RBP1 binding sites (7 nt long) [55] and the TRA-2 ISS (5 nt long) [56] are involved in both splicing activation or repression of *dsx* and *fru* pre-mRNA. Hypothesizing that in *Ae. aegypti* a similar situation exists we searched for conservation in both genes of previously characterized *cis*-elements involved in alternative splicing regulation of insects sex-specifically regulated genes.

We analyzed the *Aeafru* P1 exon and its flanking regions (500 bp-long upstream and downstream intronic sequence), using the regular expression tool of MACAW alignment software and we found: 1) nine putative type-B RBP1 binding sites (7 out of 7 nt conserved) and 2) three putative TRA-2 ISS (5 out of 5 nt conserved). Considering such high sequence conservation it is likely that these elements are involved in controlling alternative splicing of this *fru* region. In Figure S4 we provide a graphical representation of the identified putative *cis*-elements and in Table S3 their sequence and position respect to the male-specific and female-specific 5′ss. The three putative TRA-2 ISS in *Drosophila* are clustered while in *Aedes* are dispersed along the *Aeafru* P1 exon and flanking regions. Interestingly, 4 out of 9 putative RBP1 type-B binding sites form a cluster located close to the female-specific 5′ss. The RBP1 type-B binding site is present only in one copy and in a non conserved position with the respect of *Dmfru* and *Angfru* homologs. In contrast, we have found 3 sequences showing only some similarity to the 13 nt long *Drosophila* TRA/TRA-2 binding site consensus (see Table S3). Although showing some divergence, the relative positions of these 3 putative *cis* elements appear to be conserved with the respect of *Drosophila*. These putative *Aeafru* TRA/TRA-2 binding elements exhibit a higher sequence variability respect to the *Drosophila* TRA/TRA-2 binding sites, with major variations occurring within the first four and last other four bases of the 13 nt long sequence. Even though each putative *Aeafru* TRA/TRA-2 has some similarity when compared with the TRA/TRA-2 consensus of *Drosophila* (9 or 10 nucleotides identical out of 13) comparing the various *Aedes fru* TRA/TRA-2 binding sites no consensus can be defined even if slightly different from the *Drosophila* one (Figure S5). In the *Aeadsx* female-specific exons, the putative TRA/TRA-2 binding elements are similarly divergent [42].

In contrast to the situation in mosquitoes, the TRA/TRA-2 binding sites identified in other dipteran species are highly conserved in their consensus sequence and relative position in genes such as *dsx*, *fru* and *transformer* (*tra*) [43], [57], [58], [59], [60], [61], [62], [63], [64], [65], [66].

### Novel putative splicing regulatory *cis*-elements of the *Aeafru and Aeadsx* genes

We then conceived a different approach to the identification of putative regulatory *cis*-elements involved in the sex-specific alternative splicing. In *Drosophila*, as we have described before, *dsx* and *fru* share a common and highly conserved 13 nt long *cis* regulatory element, recognised by TRA and TRA-2, which is repeated various times in both genes. In *Aedes aegypti*, which apparently lacks a TRA homologue, if another analogous splicing factor plays a similar regulatory role in one sex (either the female or the male one), a parallel evolutionary constraint acting on a novel corresponding *cis* element shared by both *dsx* and *fru*, could have been maintained its recognition sequence during evolution. If this is the case, we might expect to observe a conserved multicopy distribution of a motif, forming a cluster in proximity of the regulated alternative splicing sites. Indeed the position of splicing regulatory elements within a gene has often been shown to influence their impact on splicing of its pre-mRNA and to let them work as either enhancers or silencers. This has been best studied for the SR proteins, which usually enhance splicing when bound in an exon but are inhibitory as intron-binding factors [67].

Performing a comparative analysis of the nucleotide sequences corresponding to the female-specific exons (*fru* P1-f and *dsx* 5a) of both the *Ae. aegypti* genes, flanked by 500 bp-long upstream and downstream sequences using MEME tool [68], 38 conserved motifs were identified (Figure S6, we arbitrarily selected as conserved a motif including 8–12 bp long sequence with 0 substitution and 13–15 bp long sequence with 0–2 substitutions). Most of them (28/38) are present in single copies in both *dsx* and *fru*, but 10 are present in multiple copies. Seven motifs have more then 2 copies in one or both genes (Figure S6). The 4 longer single copy motifs (11–15) are all localised either in the 2 flanking introns of *Aeadsx* or within the exon of *Aeafru*. This peculiar opposite localisation may indicate a functional significance. Previous studies have suggested that motifs of larger size seem to play important role in splicing regulation [69]. To verify if some of these identified motifs correspond to or contain described metazoan regulatory elements involved in splicing regulation, we searched in RegRNA, an integrated web server of a variety of regulatory RNA motif databases [70]. We found 9 known regulatory elements that intersect with our predicted elements (Table S4). These previously described cis elements are shorter (4–7 nt long) but contained within 14 out of 38 identified motifs. Some of these elements constitute binding sites for known RNA binding proteins, as SRp20, SRp40 or hnRNP-G, and for neuronal-specific RNA binding protein as Nova-1 protein. Their sequence conservation in both *fru* and *dsx* support their involvement in the splicing either to define exon-intron boundaries and/or to assist other *cis* regulatory elements, for example the remaining novel ones, in the specification of the sex-specific regulatory events. These results indicate a possible involvement of these conserved motifs in the regulation of the sex-specific alternative splicing of *dsx* and *fru* in *Ae. aegypti* and represent a starting point for future functional analyses.

Usually, *cis* splicing regulatory elements are present in multiple copies and can be easily expanded during evolution [71]. For example the TRA/TRA-2 binding sites involved in the autoregulation of *transformer* gene have a different copy number in other dipteran species ranging from 6 (*Ceratitis*) to 46 (*Musca domestica*) [63].

We then investigated which of the conserved motifs found in *Aeafru* and *Aeadsx* are present in multiple copies and form clusters (a localized group of repeated copies of one single cis element) or even patterns (multiple copies of a localized group of different cis elements, showing ordered succession).

**Figure 6:**
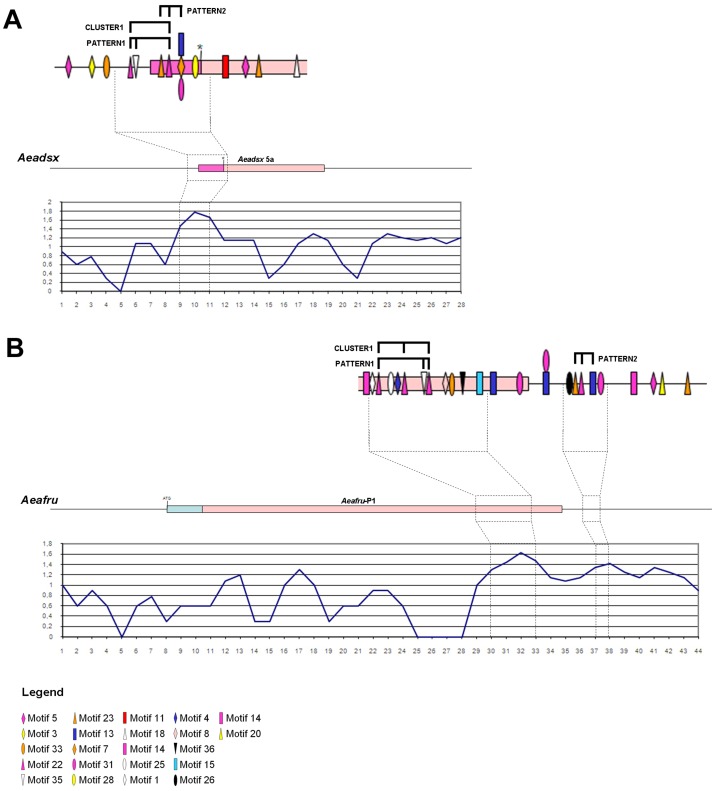
Sliding window analysis of MEME identified motifs. Schematic diagram of the sliding window analysis performed on (A) *Aeadsx* and (B) *Aeafru* sex-specifically regulated regions. Both regions are represented in scale and aligned with the corresponding sliding window graph. Each sliding window is 100 bp long and overlaps for 50 bp with the following and preceding sliding windows; each x axis position represent the nucleotide position of the centre of the sequence window. Scores (y axis) are calculated as described in Methods Section and are expressed in log_10_ of the total sliding window score multiplied for 2. Motif legend is reporter below graphs.

We performed a sliding window analysis by sampling 100 nucleotide long sequences every 50 nucleotides in both *fru* and *dsx*. We scored these windows in log_10_ (see Methods) and we graphed the results coupled with the scheme of the two analyzed regions (Figure 6). Interestingly three high-score regions, have been identified, all very close to either the female-specific *Aeadsx* 3′ss or *Aeafru* 5′ss.

Comparing the elements contained in the two regions we observed the presence of: 1) a cluster of motif 22, with two copies in *Aeadsx* and three copies in *Aeafru*; 2) a pattern, located downstream of *Aeadsx* 3′ss (in the exon) and *Aeafru* 5′ss (in the intron), composed by motifs 23-22-13 (orange-purple triangles, blue rectangle). Out of 38 motifs, the 8 nt long motif 22 (indicated in Figure 6 by a purple triangle) has the highest number of copies, with 5 in *dsx* and 6 in *fru*. The motif 23 has 2 copies in *dsx* (in the exon) and 3 in *fru* (in the intron). Motif 13 has one copy in the *Aeadsx* (exon) and 4 copies in *Aeafru* (3 flanking the exon/intron border). Hence the splicing control of *dsx* and *fru* is most likely based on more then one shared and repeated element and on more then one upstream splicing regulator recognising these elements.

### Upstream regulators of *Aeafru* in mosquitoes

As previously reported, the *tra* homologue seems to be absent in the genome of *Ae. aegypti* as well as in that of *An. gambiae* [31], [72], even though conserved in the hymenopteran *Nasonia vitripennis* genome [73].

We further searched for the presence of *tra* sequences within both genomes, using BLAST with the TRA-CAM domain of TRA [59], but no significant putative orthologues were isolated. These data suggest that the *tra* function is most likely substituted by a different splicing factor, controlling *fru* and *dsx*. However this novel splicing factor could still interact with TRA-2, as the corresponding gene is functionally conserved as essential auxiliary player in female sex determination in another dipteran species such as *Drosophila*, *Ceratitis* and *Musca* [63]. Furthermore, it is known that in the complex TRA/TRA-2 the TRA-2 protein has the stronger and more specific binding activity to TRA/TRA-2 binding sites *cis* elements [54].

TRA-2 belongs to the SR-related protein family and is directly required for female-specific splicing of *dsx* and *fru* pre-mRNA in *Drosophila* [27], [74] as well as in other distantly related dipteran species such as Tephritidae and Muscidae [75], [76]. Its secondary structure is organized in a arginine-serine rich domain, an RNA recognition motif (RRM – 81 aa long) followed by a 19 aa-long stretch named Linker region which is a unique feature of the *tra-2* homologs, and a second C-terminal RS domain. The RRM and the Linker region represent the most conserved domains of the TRA-2 proteins among dipteran and non-dipteran homologs.

The BLAST search for *tra-2*, using as virtual probe the conserved RRM and linker domains (RRM+Linker) of *D. melanogaster*, found four putative homologs within the genome of *Ae. aegypti* (AAEL009224, AAEL006416, AAEL009222 and AAEL004293) as well as two putative homologs within the genome of *An. gambiae* (AGAP006798 and a second unannotated putative *tra-2* homolog, identified starting from the EST gb|BM620287.1) The presence of multiple copies of the *tra-2* gene in these two dipteran genomes is a new feature, since *tra-2* is a single copy gene in Drosophilidae and apparently also in Tephritidae [75], [77], [78].

We investigated if this evolutionary feature had impact on the evolution of the TRA-2 sequence in mosquitoes. We compared the amino acid sequence of the RRM+Linker of the six TRA-2 homologs of mosquitoes to the corresponding domains present in *tra-2* orthologues of dipteran species (*D. melanogaster*, *C. capitata*, *Anastrepha obliqua*, *Lucilia cuprina*, *M. domestica* and *Glossina morsitans*), of the hymenopteran *Apis mellifera* and of the brachiopod crustacean *Daphnia pulex*. In addition, we included the corresponding domains of the *Homo sapiens* hTRA-2α homolog, which, although not involved in sex determination, is surprisingly able to functionally replace the endogenous *tra-2* gene in XX (chromosomally female) transgenic *Drosophila* individuals homozygous for the loss-of-function *tra-2^B^* mutation [79].

**Figure 7:**
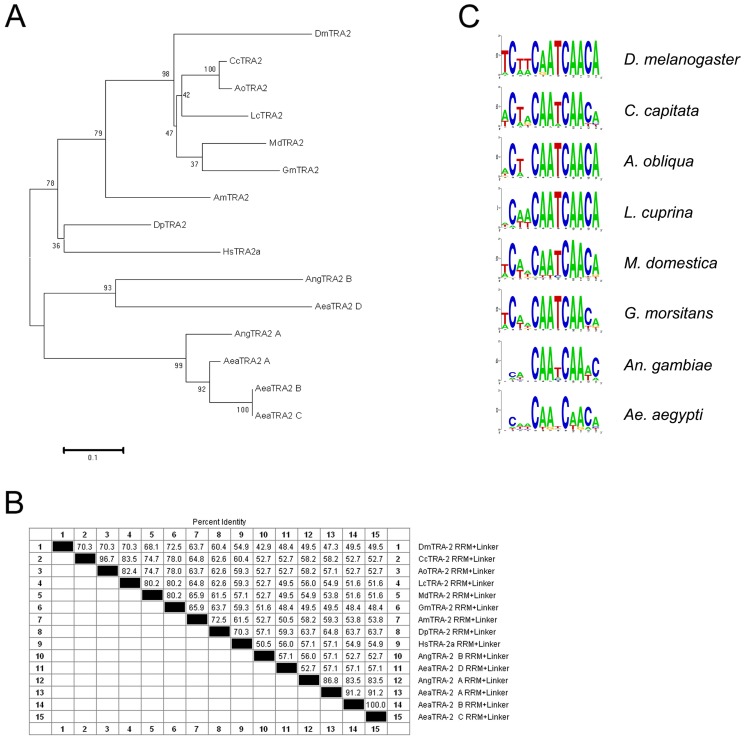
Phylogenetic and molecular evolution analyses of *tra-2* in mosquitoes. (A) NJ consensus tree based on nucleotide alignment of the RRM+Linker encoding regions of the *tra-2* gene of different insect and non insect species. (B) Table with the percentage of identity of the same nucleotide sequences analysed in the NJ tree. (C) WebLogo consensus sequences of TRA/TRA-2 binding sites of the indicated species. Only for mosquito species is it not possible to define a clear consensus sequence.

Interestingly, we found that the mosquitoes' TRA-2 RRM+Linker has the lowest amino acid conservation when compared to *Drosophila* or even *H. sapiens* TRA-2. The NJ tree of the examined amino acid sequences showed a statistically supported group including all the non-mosquito species (bootstrap percentage 78%), whereas the six mosquito TRA-2 paralogs belong to a separate group (Figure 7). This may correlate with the high degeneration of the putative TRA/TRA-2-like binding sites discernable in *Aeadsx* and *Aeafru*. Hence other upstream sex-specific splicing regulators have been most likely recruited in the mosquito lineage. Indeed preliminary results of embryonic RNAi experiments against three out of four *Ae. aegypti tra-2* paralogs failed to alter the *dsx* splicing at larval stages, or to produce intersexual phenotypes at adult stages (data not shown). Alternatively one or more of the *Aedes* TRA-2 proteins could have co-evolved a new specificity for one or more of the new identified motifs shared between *Aeadsx* and *Aeafru* genes.

### Conclusions

Sexual differentiation of *Ae. aegypti* seems to be, as in other dipteran species, under the control not only of *dsx* but also of the *fru* gene. The *Aeafru* gene is conserved in its genomic organisation even though, compared with other orthologues, the introns of *Aeafru* are much longer. Furthermore, *Aeafru* produces sex-specific transcripts from late larval, through pupal stages until adulthood, similarly to *Drosophila*. The sex-specificity of *fru* expression is achieved by an apparently conserved splicing regulation based on two 5′ alternative splice sites. This conservation of structure and sex-specific splicing suggests functional conservation, which would imply involvement of *Aeafru* in brain sexual differentiation and the control of sex behaviour.

The *fru* sex-specific regulation has been extensively studied in *D. melanogaster*, demonstrating the key role of TRA and TRA-2 splicing regulators in promoting female-specific *fru* and *dsx* splicing. In the *Aedes* genome, no TRA orthologues have been found. In contrast, four TRA-2 paralogues are present, which however group apart in a NJ phylogenetic tree with the respect of the other known dipteran and even non dipteran orthologues. Hence, most probably these TRA-2 paralogues evolved different sequence binding specificity and novel functions, after gene duplication and selective pressure relaxation. Furthermore, no well conserved TRA/TRA-2 binding sites have been found in both *fru* and *dsx* of *Aedes*, while they are highly conserved in many other dipteran species [42], [63]. Hence changes in the putative upstream splicing regulators (absence of *tra* and evolution by gene duplication and sequence divergence of *tra-2*) and in the splicing mechanisms of *dsx* gene (3′ splice site versus exon skipping) seem to be paralleled by changes in the putative *cis* acting elements. Interestingly, *fru*, unlike *dsx*, maintained a very similar 5′ alternative splicing pattern in *Aedes*, in spite of changes in the upstream splicing regulators.

We investigated whether *Aedes dsx* and *fru* might share a common sex-specific splicing regulator, even if this is not TRA and/or TRA-2. We found multiple novel motifs around the alternative sex-specific splice sites of *Aedes dsx* and *fru*, three of them forming a pattern present in both genes. Furthermore, a cluster of 3 motifs has been found to overlap the 3′ splice site region of the *Aedes dsx* female-specific first exon as well as to localise close to the *Aedes fru* female-specific 5′ splice site (200 nt away), similar to the localisation of the TRA/TRA-2 binding sites in *Drosophila fru*. It is interesting to note that motif of this cluster contains also an RBP1 binding site type B sequence. Hence, one may speculate that a sex-specific splicing factor or splicing regulatory complex might bind to these two regions. We propose that these findings may indicate a common splicing control exerted in parallel on both genes by novel sex-specific splicing factors.

We tried to approach the problem of how *fru* is sex-specifically regulated in *Ae. aegypti*. As the two alternative 5′ splice sites are apparently optimal we would expect a splicing competition mechanism.

As both the *dsx* 5a exon 3′female-specific ss and the *fru* exon P1-f 5′ female-specific ss appear to be optimal, the action of the sex-specific splicing factor would be to repress their use in males, rather than promoting it in females. This in turn implies some sex specificity of the splicing system. Furthermore, this splicing repression could be achieved by a direct action of the male-determining gene of *Aedes*, which would encode a splicing factor in this case, as proposed also for *Aeadsx* [42]. The future cloning of the *Aedes M* gene will help to understand if it directly regulates *dsx* and *fru*, promoting male-pattern splicing [80].

Further knowledge of the molecular mechanisms involved in regulation of gene expression related to sex determination and sexual differentiation in vector species, such as *Aedes aegypti*, would contribute to the development of novel control strategies whereby the vector is modified genetically for example to eliminate females, and to release sterilized males [41]. Transcriptional female-specific *cis*-acting regulatory DNA fragments have been used in combination with sex-specific alternative splicing to develop a first transgenic sexing strain in *Ae. aegypti*, [81], [82], [83]. This study will help to choose new genomic regions of either *dsx* or *fru* to build up sex-specifically expressible transgenes useful for sexing males and vector control.

## Materials and Methods

### Cloning strategy of the *Aeafru* gene

To identify the male-specific region of the *Aeafru* gene, we searched for the putative 5′ upstream portion within the genomic supercontig containing the putative *Aeafru* gene (AAEL006301 – supercontig 1.199). Using the 49 aa male-specific region of the FRU^MC^ protein of *An. gambiae* as virtual probe in a TBLASTN search, a distantly linked but highly significant hit (70% of identity; 34/49 aa identical) was obtained. Subsequently, we performed a BLASTN search on ESTs database at NCBI site (http://www.ncbi.nlm.nih.gov/) using as virtual probe a 126 bp sequence encoding the N-terminal domain starting with the common ATG codon, to isolate the upstream transcribed sequences of the *Aeafru* gene. We identified five partially overlapping ESTs (DV373639.1; DV334145.1; DV332858.1; DV324359.1; DV270086.1), containing 5′ upstream sequences located 260 kb upstream the non-sex-specific ATG codon of the *Aeafru* exon C1. Finally, we utilized the *An. gambiae* coding sequences of exons zA and zB (zA: [GenBank: AY785361]; from position +1437. zB: [GenBank: XM_311072]; from position +1314) for the *in silico* identification of putative homologous exons in *Aeafru* locus. We used these sequences as virtual probes in a BLASTX analysis on the *Ae. aegypti* genomic database and we successfully identified two regions in the supercontig 1.199 encoding the putative zing finger type A (92% of identity respect to *Anopheles* homolog) and B (72% of identity respect to *Anopheles* homolog) of the *Aeafru* gene.

### Cloning of the *fru* cDNA of *Sabethes cyaneus*

Total RNA was extracted from males (M) and females (F) of *Sa. cyaneus* adult mosquitoes using the TRIZOL Reagent (Invitrogen). Aliquots of 1 μg of each RNA were treated with RNase free-DNase I (Ambion), and first strand cDNAs were synthesized by Megascript system (Ambion) according to manufacturer's instruction. 1/40 of cDNA template was used in 50 µl PCRs containing primer pairs designed on the most conserved positions of the nucleotide alignment of BTB and zinc-finger C encoding sequences of the *Aeafru* and *Angfru* genes. The reaction mixture contained 50 mM KCl, 10 mM Tris·HCl (pH 8.3), 1.5 mM MgCl_2_, 1 µM each primer, 200 µM dNTPs (Roche), and 2.5 units *Taq* DNA polymerase (Roche). Appropriate annealing temperatures and cycle numbers were adjusted empirically for each primer pairs (Methods S1).

### Rapid amplification of cDNA ends (RACE) and sequence analyses

The 5′- and 3′- ends of the *Aeafru* cDNAs were determined with the Smart Race amplification kit (Clontech Laboratories). Reverse transcription was performed as recommended by the supplier. cDNAs containing open reading frames (ORFs) were cloned into the pGEMT-Easy Vector (Promega) and sequenced with the Applied Biosystem BigDye 1.1 sequencing kit. Sequence alignments were performed with the ClustalW software. Sequence alignments were accomplished with the ClustalW software or MAFFT online alignment tool. The following *Ae. aegypti* cDNA sequences *AeafruP1-m-C* [GenBank: JX186753], *AeafruP1-f-C* [GenBank: JX186754], *AeafruP2-C* [GenBank: JX186755] and *Sa. cyaneus* cDNA sequence *Sacfru-C* [GenBank: JX186756] were deposited in GenBank.

### Nucleic acids extractions and RT-PCR analyses

Genomic DNA and total RNA were extracted from males (M) and females (F) of *Ae. aegypti* adult mosquitoes and from the different developmental stages embryos (E), larvae of 1^st^ and 2^nd^ instar (L_12_), larvae of 3^rd^ and 4^th^ instar (L_34_), single sexed L_34_ larvae and single sexed pupae using the TRIZOL Reagent (Invitrogen). Aliquots of 1 μg of each RNA were treated with RNase free-DNase I Amplification Grade (Invitrogen) and first strand cDNAs were synthesized by Superscript First-Strand Synthesis System (Invitrogen) according to manufacturer's instruction. 1/20 of cDNA template was used in 50 µl PCRs containing primer pairs specific for the various cDNAs, 50 mM KCl, 10 mM Tris·HCl (pH 8.3), 1.5 mM MgCl_2_, 1 µM each primer, 200 µM dNTPs (Roche), and 2.5 units *Taq* DNA polymerase (Roche). Appropriate annealing temperatures and cycle numbers were adjusted to individual primer pairs (see Methods S1). Positive controls and standardization was performed as described in [42].

### Phylogenetic and evolutionary analysis

Nucleotide sequence encoding the BTB domain of the *fru* cDNAs of *Ae. aegypti* (*AeafruBTB*) and *Sa. cyaneus* (*SacfruBTB*) were aligned to the corresponding region of the *fru* gene of 21 insect species downloaded from GenBank (Methods S1) and the nucleotide sequence encoding the RRM+Linker region of the *tra-2* cDNAs of *Ae. aegypti* and *An. gambiae* were aligned to the corresponding region of the *tra-2* gene of 9 insect species downloaded from GenBank (see Methods S1) using the MAFFT software. The resulting alignment files (348 and 273 sites, respectively) was used to perform Maximum Parsimony (MP) and Neighbor-Joining analyses using the MEGA 5 software [84], with 1000 bootstrap replicates.

Nucleotide sequences encoding the BTB and the connector domain of the *fru* cDNAs of *Ae. aegypti* and *Sa. cyaneus* were separately aligned to the corresponding region of the *fru* gene of two different mosquito species, *Cu. quinquefasciatus* and *An. gambiae* using the MAFFT software. The resulting alignment files (348 sites for the BTB domain and 765 sites for the connector domain) were used to estimate the pairwise synonymous (dS) and nonsynonymous (dN) nucleotide substitution rates within the partitioned domains (BTB and connector) using the Jukes-Cantor distance model with the modified Nei-Gojobori method, implemented in MEGA 5 software. The mean pairwise ratios of dN/dS (ω) were calculated and used to examine whether the BTB and the connector domains of the available *fru* genes of mosquitoes evolve under purifying constraint for amino acid sequences (ω<1), positive selection for amino acid changes (ω>1), or neutrally (ω = 1) and to compare the selective pressures that act on the two domains.

### Measurements of splice-site strength with MaxEntScan

MaxEntScan models short sequence motifs and accounts for relationships between adjacent and non adjacent nucleotide positions to assess how well a sequence conforms to the well-established 5′ss or 3′ss consensus motif. We used these scores as an indication of splice-site strength. The 5′ss sequence is defined as position (−3, +6) and the 3′ss sequence at position (−20, +3), relative to the exon–intron junction.

### *In silico* MEME analyses

Nucleotide sequences of female-specific exon 5a of the *dsx* gene and exon P1-f of the *fru* gene of *Ae. aegypti*, flanked by 500 bp-long upstream and downstream sequences, were analyzed by MEME online tool (see Methods S1). This web server allows for the identification of short motifs in a group of related DNA or protein sequences. A motif is a sequence pattern that occurs repeatedly in a group of related protein or DNA sequences. MEME represents motifs as position-dependent letter-probability matrices which describe the probability of each possible letter at each position in the pattern. Individual MEME motifs do not contain gaps. Patterns with variable-length gaps are split by MEME into two or more separate motifs.

The search was conducted for eight times with motif lengths ranging from 8 to 15 and with 0–2 mutations per motif. Default values were used for all other parameters except for the occurrence of the motifs (set to any number of repetition) and the maximum number to search (set to 50).

With these settings we identified 105 sequences clustered in 38 conserved motifs, with p-value higher than 1.0e-4. As negative control we repeated the MEME analysis with the same parameter on shuffled sequences obtained with uShuffle java applet (k-let: 2). With these randomized sequences we cannot identified the same type and number of motifs obtained with normal sequences.

### Sliding window analysis on MEME motifs

To verify if there was an enrichment of putative regulatory elements, identified by MEME analysis, near the splice sites of *Aeadsx* and *Aeafru* sex-specifically regulated regions we performed a sliding window analysis and defined a scoring scheme, inspired to the papers of Ule et al., 2006 and Brooks et al., 2011 [53], [85]. We sampled, using a modified version of REcount perl script (available at http://splicing.rockefeller.edu/map/REcount.zip) 100 nucleotide long sequences every 50 nucleotides from the female-specific *Aeafru* P1-f and *Aeadsx* 5a exons, flanked by 500 bp-long upstream and downstream sequences. We counted for each 100 nt-long window the number of MEME identified motifs and we defined a window-score calculated in the following way:

1. 1 point for each MEME motif.
2. 1 additional point for each MEME motif with at least three copies in the *Aeafru* or *Aeadsx* analyzed sequences.
3. 1 additional point for each MEME motif with an identical MEME motif in +/−100 bp (cluster forming motifs) in the *Aeafru* or *Aeadsx* analyzed sequences.
4. 1 additional point for each MEME motif with at least two different MEME motifs in +/−200 bp with the same relative order (pattern forming motifs) in the *Aeafru* or *Aeadsx* analyzed sequences. This is and additive point i.e. if a MEME motif forms two different patterns it gains +2 points, if it forms 3 different patterns it gains +3 points etc.

The total score for each window was expressed as the log_10_ (2× Score).

## Supporting Information

Figure S1 **Censor analysis of** ***Aeafru*** **introns.** Graphical output of Censor analysis on *Aeafru* intron sequences. For a legend see: http://www.girinst.org/censor/help.html#GRAPH. For the intron 1 (424 kb-long) the output is reported for the whole sequence and for 42,4 kb-long sub regions of the same intron. For each *Aeafru* intron the number of repetitive elements per kb (NoRE/kb) and the percentage of repetitive element nucleotides respect to intron nucleotides (REbp) are reported.
(PDF)

Figure S2 **Microsyntheny of mosquitoes** ***fru*** **containing regions.** Ensembl genome browser view of the *fru* containing regions of *Ae. aegypti* and *An. gambiae* genomes. The homologues are connected by braked lines. The green arrow indicates a putative chromosomal breakpoint site involved in genomic rearrangement after the split of the two species. Only 3 genes (*Aeafru*, AAEL006293 and AAEL006290) out of 22 present in the *Aedes* supercontig 1.199 exhibit conserved synthenic relationship with their *Anopheles* putative orthologues (*Angfru*, AGAP00079 and AGAP00078, respectively); of the remaining 19, 3 genes (AAEL006304, AAEL006285 and AAEL006288) have not a homolog in *Anopheles* and 16 correspond to *Anopheles* putative homologs located in different genomic positions. Interestingly, microsynteny was found between the second half of the *Aedes* supercontig 1.199, downstream the *fru* gene, and a genomic region located on the chromosome 3R of *Anopheles* (position 5, 7–5, 8 Mb). We identified two duplication events occurred in this region of *Ae. aegypti*, with the AAEE006296 and the AAEL006292 genes corresponding to the *Anopheles* AGAP008103 gene and the AAEL006302 and AAEL006289 corresponding to the *Anopheles* AGAP008101 gene. The *Anopheles fru-*containing region contains 15 genes. 3 out of 15 genes exhibit synthenic relationship with the *Aedes* putative homologs, including *Angfru* (AGAP00080 – *fru*, AGAP00078 and AGAP00079). Of the remaining 12, one gene has no homolog in *Aedes* (AGAP00084) and 11 correspond to *Aedes* putative homologs located in different genomic positions. For these genes we observed a peculiar situation, with 4 couples of *Anopheles* genes corresponding to couples of *Aedes* putative orthologues located in 4 different *Aedes* supercontigs (AGAP013356 and AGAP00075 – *Aedes* supercontig 1.127; AGAP013406 and AGAP00076 – *Aedes* supercontig 1.487; AGAP00081 and AGAP00082 – *Aedes* supercontig 1.166; CPR129 and AGAP00085 – *Aedes* supercontig 1.894). This finding suggests that the *fru-*containing region in *Anopheles* has been involved in multiple genome shuffling events after the split of the two species, according with previous. Finally, we identified two intronic genes (AGAP00088 and AGAP013490) located within the gene AGAP00086, corresponding to the *Aedes* orthologue AAEL013684, and hence, most probably, due to a duplication event in *An. gambiae*.
(PDF)

Figure S3 **Exon/Intron junctions and MaxEntScan scores of** ***fru*** **genes.** A) Coding sequences are shown in upper case letters and non-coding regions in lower case letters. The 5′ss consensus sequence is MAG/GTRAGT and the 3′ss consensus is YnNYAG. The number of pyrimidines (N° of Y) in the 12 bp preceding the 3′ ss (NYAG) is indicated for each 3′ss. The consensus number of pyrimidines for *Ae. aegypti* (8,02±2,15) is derived from the tabulation of 4688 *Ae. aegypti* splice-acceptor sites [36]. M = A or C. R = A or G nucleotide. Y = T or C or nucleotide. N = any nucleotide. B) Schematic representation of fru-P1 gene. Shaded in grey negative MaxEntScan scores. The scores are in bits and a higher MaxEntScan score correspond to a stronger splice-site sequence. A ss with a MaxEntScan score of 12–13 is a strong ss while negative scores are usually associated with decoy splicing sites (Yeo G. pers. comm.).
(PDF)

Figure S4 **Schematic representation of the position of the identified putative** ***cis*** **-elements involved in splicing regulation of** ***Aeadsx*** **and** ***Aeafru*** **.** We identified in *Aeafru* P1 exon an additional putative TRA/TRA-2 binding site located close to the male-specific 5′ss, which appears to be highly conserved in the very same region of *Drosophila fru*, although previously not reported, and in the *Anopheles* orthologue *Angfru* genes (data not shown).
(PDF)

Figure S5 **Consensus sequences of TRA/TRA-2 binding sites.** Consensus sequences of TRA/TRA-2 binding sites of *D. melanogaster*, *An. gambiae* and *Ae. aegypti dsx*, *fru* and *dsx+fru* genes obtained with WebLogo web tool. The absence of a consensus is clear for mosquitoes genes.
(PDF)

Figure S6 **Graphical representation and list of motifs identified by MEME analysis.** Schematic graphical representation and tabular list of the motifs identified by MEME analysis in *Aeafru* and *Aeadsx* genes.
(PDF)

Methods S1 **Lists of primers, GenBank accession numbers and web tools utilized in this paper.**
(PDF)

Table S1 **Modified Censor output of repetitive elements identified in** ***Aeafru*** **intronic regions.** The identified repetitive elements greatly vary in length and among them the most abundant are the NON-LTR/Jockey LINE-1_AA element [86], detected in 38 copies, and the NON-LTR/SINE Feilai elements [87], detected in 36 copies.
(PDF)

Table S2 ***Ae. aegypti*** **intron analysis.** Tabular output of *Aedes aegypti* intron analysis. The average number of repetitive elements per kb (indicated as NoRE/kb) in *Aeafru* introns is 2,03±0,28 while the average percentage of nucleotides of the identified repetitive elements with the respect to the nucleotides of the *Aeafru* introns (indicated as REbp) is 34,56%±19,54 (Figure S1). To compare these values with the average values of the *Ae. aegypti* introns, we analysed 1000 randomly chosen *Ae. aegypti* introns (size range from 1 to 130 kb) with the CENSOR software, defining an average NoRE/kb (2,19±0,68) and REbp (47,22±17,75%) intron values for this species. This analysis indicates that within the introns of *Ae. aegypti* the number of repetitive elements per kb is almost constant, with a value of about 2, while the size of these elements is variable, ranging from 30% to 60% of the whole intron sequences. The NoRE/kb and REbp values of the *Aeafru* introns do not deviate significantly this observation.
(PDF)

Table S3 **Regulatory elements in the sex-specifically regulated region of** ***fru*** **homologs.** Sequence of the putative *Aeafru cis* identified in the female-specific exon P1-f. The upper case indicate conserved nucleotides respect to the consensus sequences of *Drosophila*. The distance of these elements from the male- and female-specific 5′ splicing donor sites are indicated.
(PDF)

Table S4 **Correspondence between MEME identified motifs and motif from RegRNA database.** The nucleotides of MEME motifs corresponding to RegRNA motifs areshaded in light grey.
(PDF)
