# Supplementary material for: The Orthologue of the Fruitfly Sex Behaviour Gene Fruitless in the Mosquito Aedes aegypti: Evolution of Genomic Organisation and Alternative Splicing
Source: PLoS One. 2013 Feb 13;8(2):e48554. doi: 10.1371/journal.pone.0048554 (PMC3572092; doi:10.1371/journal.pone.0048554)
Supplement: Figure S1 — Censor analysis of Aeafru introns. Graphical output of Censor analysis on Aeafru intron sequences. For a legend see: http://www.girinst.org/censor/help.html#GRAPH. For the intron 1 (424 kb-long) the output is reported for the whole sequence and for 42,4 kb-long sub regions of the same intron. For each Aeafru intron the number of repetitive elements per kb (NoRE/kb) and the percentage of repetitive element nucleotides respect to intron nucleotides (REbp) are reported. (PDF) [file pone.0048554.s001.pdf]

Figure S1 – Censor analysis of *Aeafru* introns

Intron 1

Length: 424374 Bp

Censor graphic output:

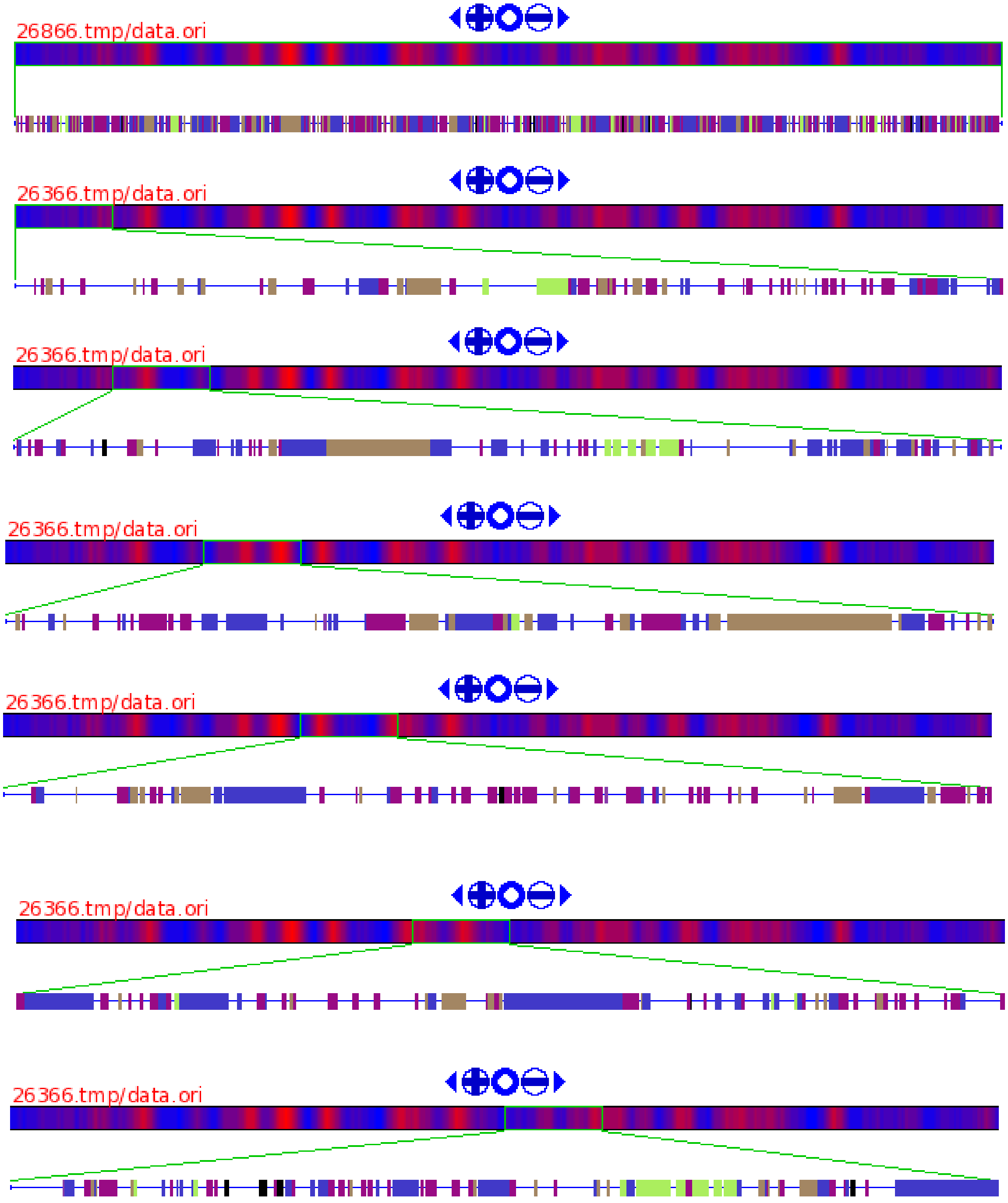

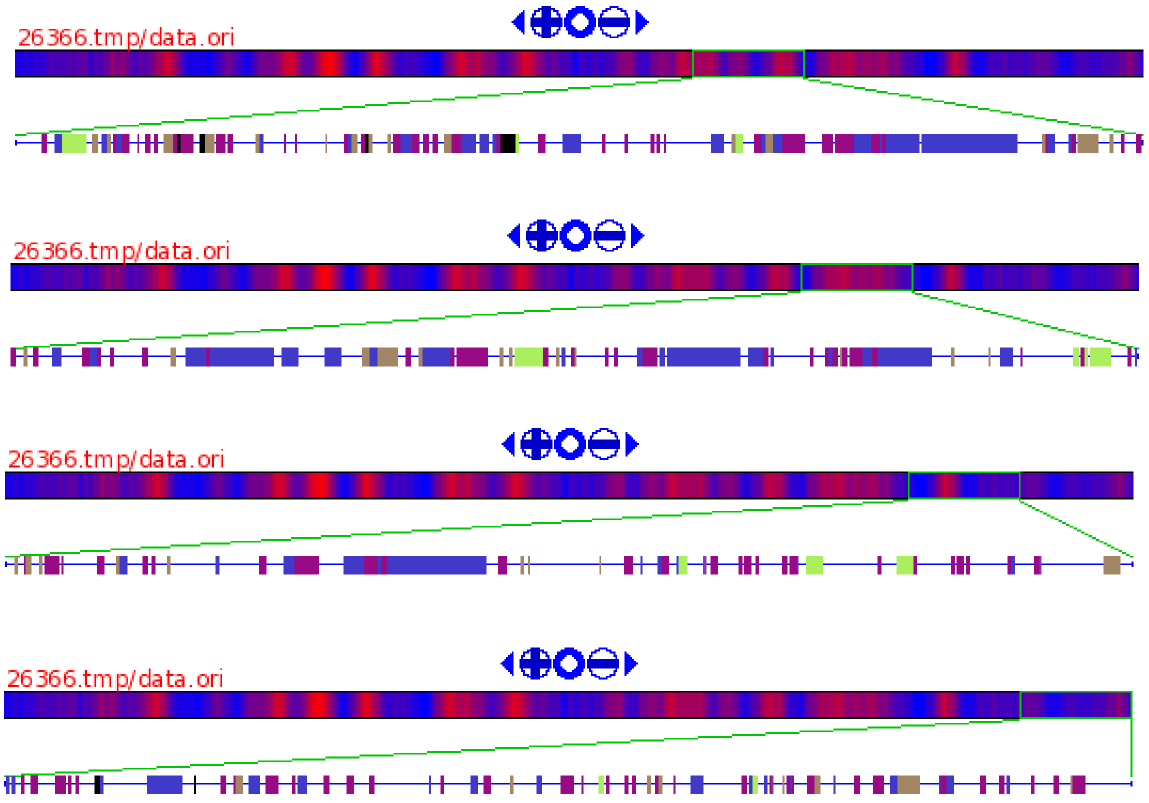

Number of Number of repetitive elements per kb (NoRE/kb): 1,95

Percentage of repetitive element nucleotides respect to intron nucleotides (REbp): 41,8%

Intron 2

Length: 9859 Bp

Censor graphic output:

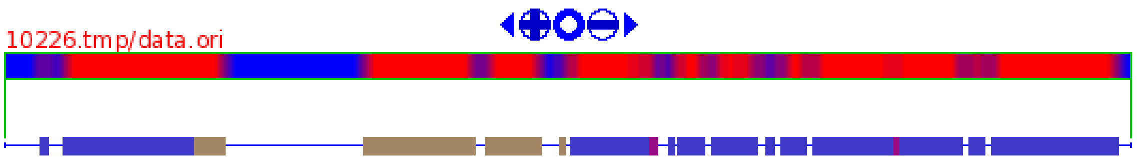

Number of Number of repetitive elements per kb (NoRE/kb): 2,55

Percentage of repetitive element nucleotides respect to intron nucleotides (REbp): 72,5%

Intron 3

Length: 75 Bp

Number of Number of repetitive elements per kb (NoRE/kb): 0

Percentage of repetitive element nucleotides respect to intron nucleotides (REbp): 0

Intron 4

Length: 8321 Bp

Censor graphic output:

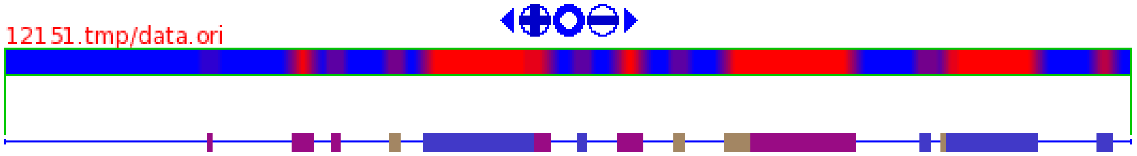

Number of Number of repetitive elements per kb (NoRE/kb): 1,92

Percentage of repetitive element nucleotides respect to intron nucleotides (REbp): 40,6%

Intron 5

Length: 46633 Bp

Censor graphic output:

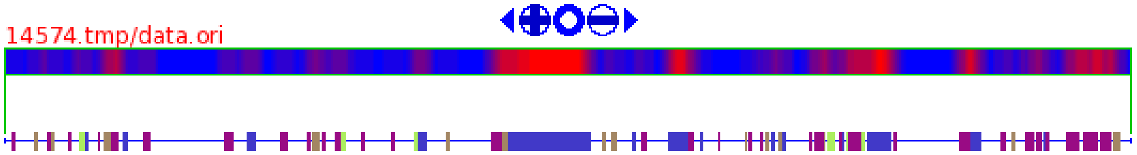

Number of Number of repetitive elements per kb (NoRE/kb): 1,89

Percentage of repetitive element nucleotides respect to intron nucleotides (REbp): 32,2%

<u>Intron 6</u>

Length: 39889 Bp

Censor graphic output:

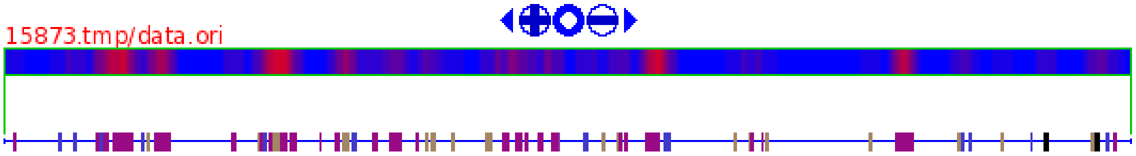

Number of Number of repetitive elements per kb (NoRE/kb): 1,92

Percentage of repetitive element nucleotides respect to intron nucleotides (REbp): 19,6%

Average values for *Aeafru* introns (excluding intron 3):

Average *Aeafru* NoRE/kb: 2,03 ± 0,28 and

Average *Aeafru* REbp: 34,56 % ± 19,54
