# Supplementary figures and images for: The Orthologue of the Fruitfly Sex Behaviour Gene Fruitless in the Mosquito Aedes aegypti: Evolution of Genomic Organisation and Alternative Splicing
Source: PLoS One. 2013 Feb 13;8(2):e48554. doi: 10.1371/journal.pone.0048554 (PMC3572092; doi:10.1371/journal.pone.0048554)

**Figure S2 - Microsynthemy of mosquitoes fru containing regions**

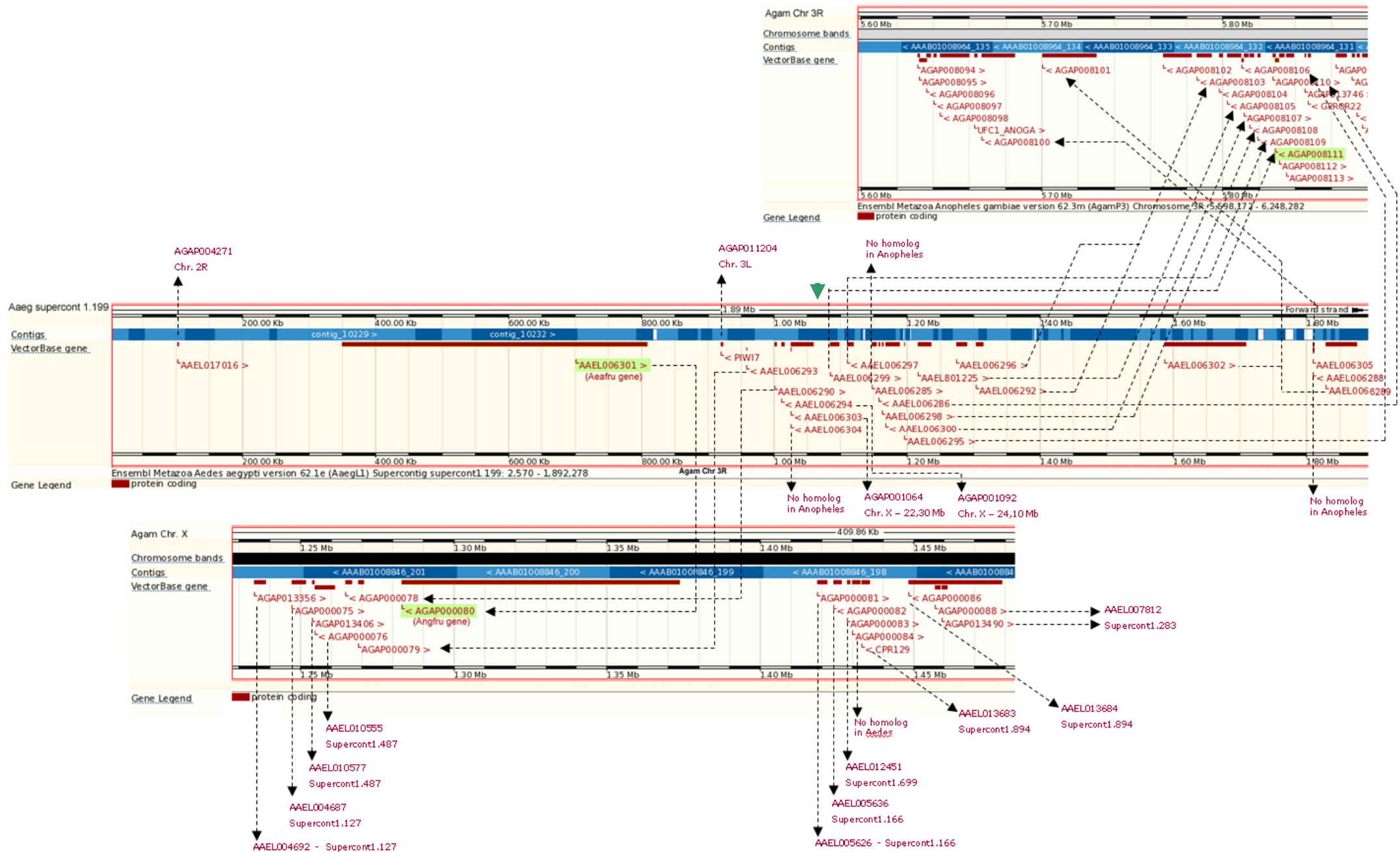

Supplement: Figure S2 — Microsyntheny of mosquitoes fru containing regions. Ensembl genome browser view of the fru containing regions of Ae. aegypti and An. gambiae genomes. The homologues are connected by braked lines. The green arrow indicates a putative chromosomal breakpoint site involved in genomic rearrangement after the split of the two species. Only 3 genes (Aeafru, AAEL006293 and AAEL006290) out of 22 present in the Aedes supercontig 1.199 exhibit conserved synthenic relationship with their Anopheles putative orthologues (Angfru, AGAP00079 and AGAP00078, respectively); of the remaining 19, 3 genes (AAEL006304, AAEL006285 and AAEL006288) have not a homolog in Anopheles and 16 correspond to Anopheles putative homologs located in different genomic positions. Interestingly, microsynteny was found between the second half of the Aedes supercontig 1.199, downstream the fru gene, and a genomic region located on the chromosome 3R of Anopheles (position 5, 7–5, 8 Mb). We identified two duplication events occurred in this region of Ae. aegypti, with the AAEE006296 and the AAEL006292 genes corresponding to the Anopheles AGAP008103 gene and the AAEL006302 and AAEL006289 corresponding to the Anopheles AGAP008101 gene. The Anopheles fru-containing region contains 15 genes. 3 out of 15 genes exhibit synthenic relationship with the Aedes putative homologs, including Angfru (AGAP00080 – fru, AGAP00078 and AGAP00079). Of the remaining 12, one gene has no homolog in Aedes (AGAP00084) and 11 correspond to Aedes putative homologs located in different genomic positions. For these genes we observed a peculiar situation, with 4 couples of Anopheles genes corresponding to couples of Aedes putative orthologues located in 4 different Aedes supercontigs (AGAP013356 and AGAP00075 – Aedes supercontig 1.127; AGAP013406 and AGAP00076 – Aedes supercontig 1.487; AGAP00081 and AGAP00082 – Aedes supercontig 1.166; CPR129 and AGAP00085 – Aedes supercontig 1.894). This finding suggests that the fru-containing region in [file pone.0048554.s002.pdf]

Figure S6 - Graphical representation and list of motifs identified by MEME analysis

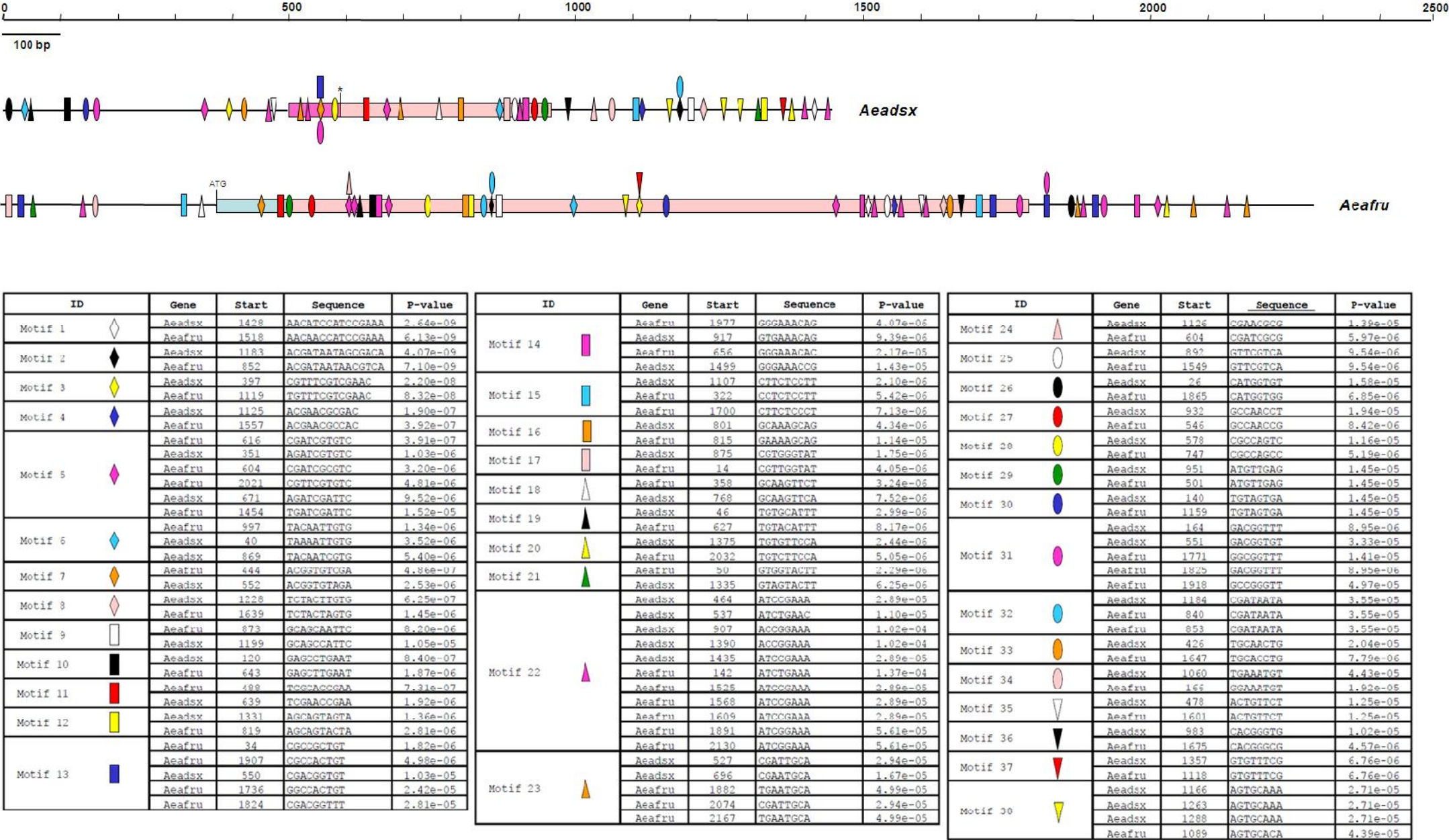

Supplement: Figure S6 — Graphical representation and list of motifs identified by MEME analysis. Schematic graphical representation and tabular list of the motifs identified by MEME analysis in Aeafru and Aeadsx genes. (PDF) [file pone.0048554.s006.pdf]
