# Supplementary material for: The Orthologue of the Fruitfly Sex Behaviour Gene Fruitless in the Mosquito Aedes aegypti: Evolution of Genomic Organisation and Alternative Splicing
Source: PLoS One. 2013 Feb 13;8(2):e48554. doi: 10.1371/journal.pone.0048554 (PMC3572092; doi:10.1371/journal.pone.0048554)
Supplement: Figure S3 — Exon/Intron junctions and MaxEntScan scores of fru genes. A) Coding sequences are shown in upper case letters and non-coding regions in lower case letters. The 5′ss consensus sequence is MAG/GTRAGT and the 3′ss consensus is YnNYAG. The number of pyrimidines (N° of Y) in the 12 bp preceding the 3′ ss (NYAG) is indicated for each 3′ss. The consensus number of pyrimidines for Ae. aegypti (8,02±2,15) is derived from the tabulation of 4688 Ae. aegypti splice-acceptor sites [36]. M = A or C. R = A or G nucleotide. Y = T or C or nucleotide. N = any nucleotide. B) Schematic representation of fru-P1 gene. Shaded in grey negative MaxEntScan scores. The scores are in bits and a higher MaxEntScan score correspond to a stronger splice-site sequence. A ss with a MaxEntScan score of 12–13 is a strong ss while negative scores are usually associated with decoy splicing sites (Yeo G. pers. comm.). (PDF) [file pone.0048554.s003.pdf]

Figure S3 – Exon/Intron junctions and MaxEntScan scores of *fru* genes

A

Aeafru gene

Exon No.	Exon size (bp)	5'ss	Intron size (bp)	3'ss	N° of Y
P1-m	149	R R<br>CCGAAG/gtatgttgagcggggt	425673	N S<br>tctttttctttttgcag/GAACAG	12
P1-f	1299	n.c.<br>CGGCTG/gtaagtactttggtgg	424374	N S<br>tctttttctttttgcag/GAACAG	12
C1	148	V K<br>GTCAAA/gtaagtattctatggg	9859	A H<br>tcctacttctccgtag/GCACAT	11
C2	201	L K<br>TTGAAA/gtaagttcccgatgcg	75	V R<br>ctttcccctattttag/GTACGA	11
C3	516	S R<br>TCTAGA/gtttatatgcaaatct	8321	Q H<br>ttaggaagggtttgag/CAACAC	4
C4	279	L P<br>TTGCCG/gtaagtgatgagtttc	46633	P I<br>ttttttttttccttcag/CCGATC	12
C5	130	D G<br>TCGACG/gtaagcgaacacgatc	39889	P G<br>cctctgttggttgcag/GGCCGG	9
zC	693	Q M *<br>CAGATGTAAaaccttcagatcat			
Consensus		MAG/gtRagt		YYYYYYYYYYYNYag/G	8,02 +/- 2,15

B

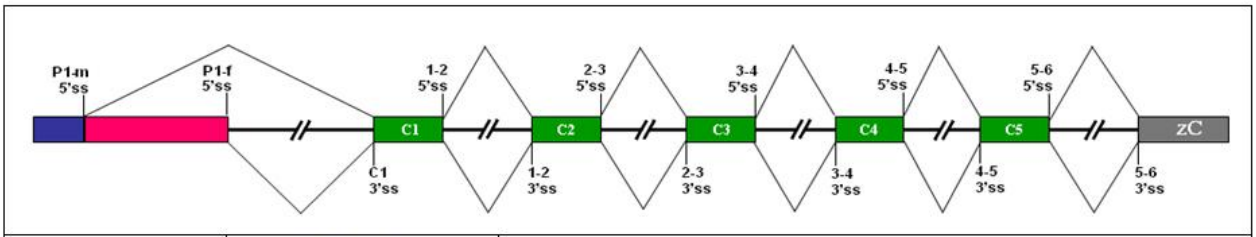

Splicing Site Name	MaxEntScan Score			Splicing site sequence		
	Dm	*Ang*	*Aea*	*Dm*	*Ang*	*Aea*
P1-m 5'ss	8,89	10,65	9,79	TAGgtaagcct	CAGgtacgtcc	AAGgtatgttg
P1-f 5'ss	11,37	-5,34	10,65	GCGgtaagtga	TACgtccgctg	CTGgtaagtac
C1 3'ss	8,28	7,62	12,09	atttatactctatattacagGTA	ccctcttccaccgcgaacagGAA	ttgctcttttctttttgcagGAA
1-2 5'ss	9,49	4,06	9,72	AAGgtgcgtcc	AAAgtaaatgc	AAAgtaagtat
1-2 3'ss	8,79	7,00	9,91	tcatattcattctattgcagGCT	ctctgtttgctgttgcacagGCA	cgtttcctacttctccgtagGCA
2-3 5'ss	6,99	10,51	9,72	CAGgtgtgtgc	AAGgtaagggg	AAAgtaagttc
2-3 3'ss	6,79	10,90	12,10	gttatttgggacttttccagGTG	tttcctgccatttgttacagGTA	catactttcccctattttagGTA
3-4 5'ss	9,60	10,12	-11,54	CAGgtgagcac	CAGgtacgccg	AGAgtttatat
3-4 3'ss	5,26	11,11	-15,88	agtactcaacttgtttgcagGCC	cctcgccccgtcccttt cagGCC	tgttttaggaagggtttgagCAA
4-5 5'ss	10,91	10,28	10,91	CCGgtaagtgt	CAGgtaggtgt	CCGgtaagtga
4-5 3'ss	-0,51	4,92	9,86	atttcttgctgcaaccaaagAGC	ctttcggtttgtggctacagCCA	acttttttttttccttcagCCG
5-6 5'ss	11,45	8,27	9,89	ACGgtgagtag	ATGgtgagcga	ACGgtaagcga
5-6 3'ss	7,81	11,11	9,34	ttctcgttctcgaaccgcagGCA	ctttctcttgtcggttgcagGGC	tgttcctctgttggttgcagGGC

A) Coding sequences are shown in upper case letters and non-coding regions in lower case letters. The 5'ss consensus sequence is MAG/GTRAGT and the 3'ss consensus is Y_nNYAG (Mount et al. 1992). The number of pyrimidines (N° of Y) in the 12 bp preceding the 3' ss (NYAG) is indicated for each 3'ss. The consensus number of pyrimidines for *Ae. aegypti* (8,02±2,15) is derived from the tabulation of 4688 *Ae. aegypti* splice-acceptor sites (Salvemini et al., 2011). M= A or C. R=A or G nucleotide. Y=T or C or nucleotide. N=any nucleotide. B) Schematic representation of fru-P1 gene. Shaded in grey negative MaxEntScan scores.
