# Supplementary material for: The Orthologue of the Fruitfly Sex Behaviour Gene Fruitless in the Mosquito Aedes aegypti: Evolution of Genomic Organisation and Alternative Splicing
Source: PLoS One. 2013 Feb 13;8(2):e48554. doi: 10.1371/journal.pone.0048554 (PMC3572092; doi:10.1371/journal.pone.0048554)
Supplement: Figure S4 — Schematic representation of the position of the identified putative cis -elements involved in splicing regulation of Aeadsx and Aeafru . We identified in Aeafru P1 exon an additional putative TRA/TRA-2 binding site located close to the male-specific 5′ss, which appears to be highly conserved in the very same region of Drosophila fru, although previously not reported, and in the Anopheles orthologue Angfru genes (data not shown). (PDF) [file pone.0048554.s004.pdf]

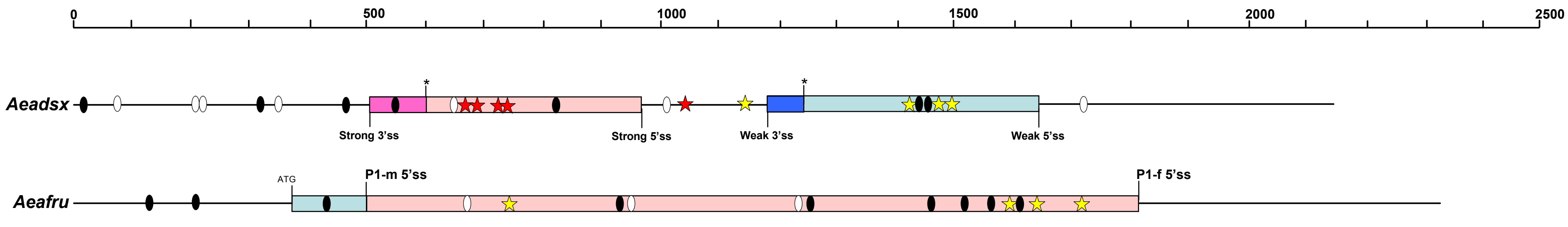

Aeadsx

TRA-2-ISS
AEADSX   77 CAAGA
AEADSX 203 CAAGA
AEADSX 212 CAAGA
AEADSX 349 CAAGA
AEADSX 650 CAAGA
AEADSX 1013 CAAGA
AEADSX 1719 CAAGG

RBP1 – TYPE B
AEADSX   21 ATCCACA
AEADSX 318 ATCTCTA
AEADSX 464 ATCCGAA
AEADSX 537 ATCTGAA
AEADSX 819 ATCTAGA
AEADSX 1435 ATCCGAA
AEADSX 1459 ATCTTCA

TRA/TRA-2 b. s.
AEADSX 1143 AATACAAACAACA
AEADSX 1419 TCAACAAGCAACA
AEADSX 1460 TCTTCAACCAACC
AEADSX 1475 CCTACAATCTACA

Nv TRA/TRA-2 binding motif
Aeadsx 663 CGAAGATC
Aeadsx 684 GGAAGAAG
Aeadsx 705 AGAAGAAT
Aeadsx 718 CGAAGAAA
Aeadsx 1041 AGAAGAAT

Aeafru

TRA-2-ISS
Aeafru 653 CAAGG
Aeafru 985 CAAGG
Aeafru 1228 CAAGA

RBP1 – Type B
Aeafru 142 ATCTGAA
Aeafru 227 ATCTAGA
Aeafru 432 ATCCCCA
Aeafru 954 ATCTTTA
Aeafru 1243 ATCCGTA
Aeafru 1468 ATCTAAA
Aeafru 1525 ATCCGAA
Aeafru 1568 ATCCGAA
Aeafru 1609 ATCCGAA

TRA/TRA-2 b. s.
Aeafru 731 ATAACAATCAAAA
Aeafru 1591 TCATCAATCTACT
Aeafru 1648 GCACCTGTCAACA
Aeafru 1705 CCCTCAATCAGCA

Figure S4 – Schematic representation of the position of the identified putative *cis*-elements involved in splicing regulation of *Aeadsx* and *Aeafru*.
