# Supplementary material for: The Orthologue of the Fruitfly Sex Behaviour Gene Fruitless in the Mosquito Aedes aegypti: Evolution of Genomic Organisation and Alternative Splicing
Source: PLoS One. 2013 Feb 13;8(2):e48554. doi: 10.1371/journal.pone.0048554 (PMC3572092; doi:10.1371/journal.pone.0048554)
Supplement: Figure S5 — Consensus sequences of TRA/TRA-2 binding sites. Consensus sequences of TRA/TRA-2 binding sites of D. melanogaster, An. gambiae and Ae. aegypti dsx, fru and dsx+fru genes obtained with WebLogo web tool. The absence of a consensus is clear for mosquitoes genes. (PDF) [file pone.0048554.s005.pdf]

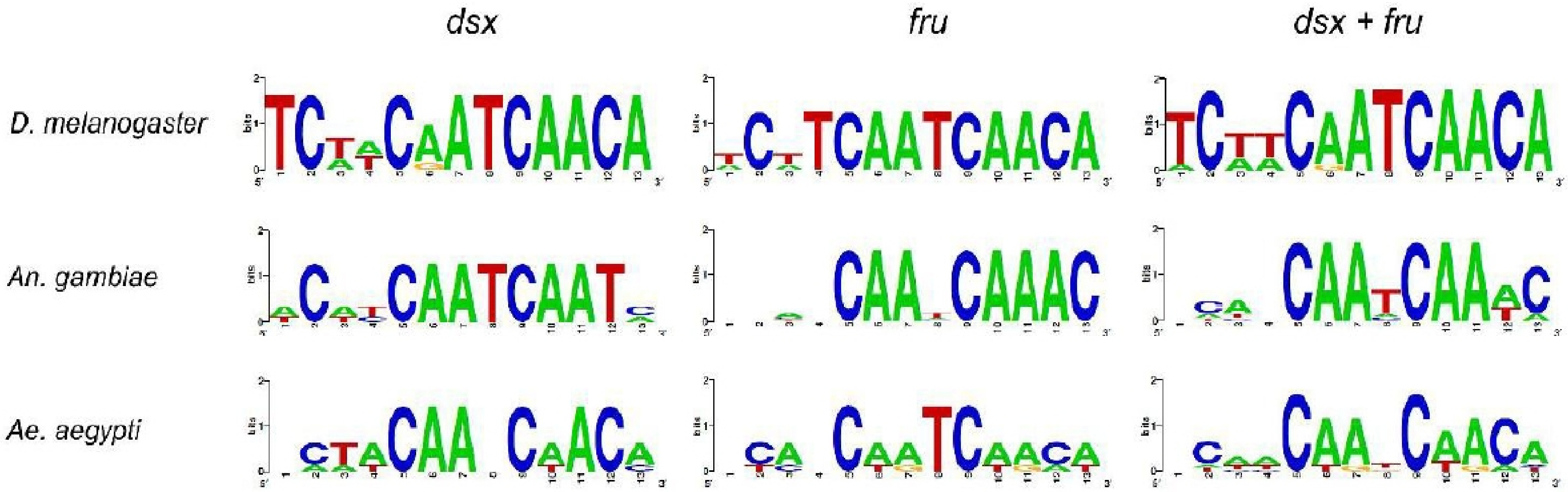

Figure S5 - Consensus sequences of TRA/TRA-2 binding site of *D. melanogaster*, *An. Gambiae* and *Ae. aegypti dsx*, *fru*, and *dsx*+*fru* genes obtained with Weblogo web tool. The used sequences are reported below.

Dmdsx
>1
TCTTCAATCAACA
>2
TCTTCAATCAACA
>3
TCTACAATCAACA
>4
TCTTCAATCAACA
>5
TCAACAATCAACA
>6
TCAACGATCAACA

Dmfru
>1
ACTTCAATCAACA
>2
TCTTCAATCAACA
>3
TCATCAATCAACA

Angdsx
>1
TCTCCAATCAATC
>2
ACATCAATCAATC
>3
ACATCAATCAATA

Angfru
>1
TAAACAAACAAAC
>2
GTATCAATCAAAC
>3
CCTTCAACCAAAC
>4
TAAACAATCAAAC
>5
CCCGCAATCAAAC

Aeadsx
>1
TCAACAAGCAACA
>2
TCTTCAACCAACC
>3
CCTACAATCTACA
>4
AATACAAACAACA

Aeafru
>1
ATAACAATCAAAA
>2
TCATCAATCTACT
>3
GCACCTGTCAACA
>4
CCCTCAATCAGCA
