# Supplementary material for: The Orthologue of the Fruitfly Sex Behaviour Gene Fruitless in the Mosquito Aedes aegypti: Evolution of Genomic Organisation and Alternative Splicing
Source: PLoS One. 2013 Feb 13;8(2):e48554. doi: 10.1371/journal.pone.0048554 (PMC3572092; doi:10.1371/journal.pone.0048554)
Supplement: Methods S1 — Lists of primers, GenBank accession numbers and web tools utilized in this paper. (PDF) [file pone.0048554.s007.pdf]

Methods S1

Primers for *Aedes aegypti fru* cloning and developmental analyses

fru1: 5'-CGGCATTGAACGGCTATCC-3'
fru1rev: 5'-CCTTCGGTGCGATTGAAGC-3'
fru1f: 5'-GCGTCATCGATCGTGTCTTG-3'
fru2f: 5'-GTTTGCCAGCACTTCTCCC -3'
fru2rev: 5'-GACAGTATCGCTTGATGTGC-3'
fru1c: 5'-CAATACCGAGTGGTGTGTGG-3'
fru3: 5'-GTTCTCTCTGCAGCTCTCG-3'
fruL1: 5'-TACTGTTGCAAGTTCTCAATAG-3'
fruL2: 5'-CAATACCGAGTGGTGTGTGG-3'
Aearp49+: 5'-CCAAGATCGTCAAGAAGCGG-3'
Aearp49-: 5'-GGTTGGTCACAGCGATGG-3'
dsx3: 5'-GATACTGAAAGGCGCCGACG-3'
dsx5: 5'-GCAGAATATGGGACTGGTGC-3'

Primers for *Sabethes cyaneus fru* cloning

AeaWHH+: 5'-GCTGGAACAACCATCAG-3';
AeaYQG-: 5'-GTTCACCTCGCCCTGGTAC-3'
SacBTB+**:** 5'-GCATCCCATCATCTACCTGC-3'
AeaZNC-: 5'-TGACCTCCTTGCCGCACG-3'

GenBank accession numbers of sequences used in phylogenetic and evolutionary analysis

Drosophila melanogaster DmfruBTB, [GenBank: NP_524397]
D. sechellia DsfruBTB, [GenBank: XP_002038222]
D. erecta DefruBTB, [GenBank: XP_001979637]
D. ananassae DafruBTB, [GenBank: XP_001954108]
D. pseudobscura DpfruBTB, [GenBank: XP_001358536]
D. willistoni DwfruBTB, [GenBank: XP_002073544]
D. virilis DvfruBTB, [GenBank: XP_002056235]
D. grimshavi DgfruBTB, [GenBank: XP_001990228]
Ceratitis capitata CcfruBTB, [GenBank: ACH81147]
Lutzomyia longipaplalis LlfruBTB, [GenBank: AM090394]
Anopheles gambiae AngfruBTB, [GenBank: AAV52865]
Culex quinquefasciatus CuqfruBTB, [GenBank: XP_001860373]
Danaus plexippus DapfruBTB, [GenBank: EY257161]
Bombyx mori BmfruBTB, [GenBank: AU000406]

Tribolium castaneum TcfruBTB, [GenBank: NP_001157690]

Chorthippus brunneus CbfruBTB, [GenBank: ABD85043.1]

Schistocerca gregaria SgfruBTB, [GenBank: ADM86718.1]

Blattella germanica BgfruBTB, [GenBank: CAZ68905.1]

Nasonia vitripennis NvfruBTB, [GenBank: NP_001157595]

Apis mellifera AmfruBTB, [GenBank: XP_392552]

Harpegnathos saltator HasfruBTB, [GenBank: EFN81805]

D. melanogaster DmTRA-2 [GenBank: NM_057416.2]

C. capitata CcfruBTB CcTRA-2 [GenBank: EU999754.1]

Anastrepha obliqua AoTRA-2 [GenBank: FN658607.1]

Lucilia cuprina LcTRA-2 [GenBank: FJ461620.1]

Glossina morsitans GmTRA-2 [GenBank: DV608906.1]

Musca domestica MdTRA-2 [GenBank: AY847518.1]

Ae. aegypti AeaTRA-2_A [GenBank: AAEL009224]

Ae. aegypti AeaTRA-2_B [GenBank: AAEL006416]

Ae. aegypti AeaTRA-2_C [GenBank: AAEL009222]

Ae. aegypti AeaTRA-2_D [GenBank: AAEL004293]

An. gambiae AngTRA-2_A [GenBank: AGAP006798]

An. gambiae AngTRA-2_B [GenBank: BM620287.1]

A. mellifera AmTRA-2 [GenBank: XP_001121070.2]

Daphnia pulex DpTRA-2 [GenBank: EFX90042.1]

Homo sapiens HsTRA_2α [GenBank: NP_037425]

Databases and web tools

-RegRNA
(http://regrna.mbc.nctu.edu.tw/index.php)

-TRANSFAC
(http://www.gene-regulation.com/pub/databases/transfac/doc/toc.html)

-PROMO (Messeguer et al. 2002, Farrè et al. 2003)
(http://alggen.lsi.upc.es/)

-CENSOR (Jurka *et al.* 1996)
(http://www.girisnst.org/censor/index.php)

-BLASTX, BLASTN, TBLASTN and TBLASTX
(http://blast.ncbi.nlm.nih.gov/)

-Promoter 2.0 (Knudsen, 1999)
(www.cbc.dtu.dk/services/Promoter)

-BDGP Neural Network Promoter Prediction (Reese, 2001)
(www.fruitfly.org:9005/seq_tools/promoter)

-GPMiner (Lee et al., 2012)
(http://gpminer.mbc.nctu.edu.tw/index.php).

-Footprinter 3.0 (Blanchette and Tompa 2003; Fang and Blanchette 2006)

(http://wingless.cs.washington.edu/htbin-post/unrestricted/FootPrinterWeb/FootPrinterInput2.pl)

-MaxEntScore algorithms

(http://genes.mit.edu/burgelab/maxent/Xmaxentscan_scoreseq.html)
