# Supplementary material for: The Orthologue of the Fruitfly Sex Behaviour Gene Fruitless in the Mosquito Aedes aegypti: Evolution of Genomic Organisation and Alternative Splicing
Source: PLoS One. 2013 Feb 13;8(2):e48554. doi: 10.1371/journal.pone.0048554 (PMC3572092; doi:10.1371/journal.pone.0048554)
Supplement: Table S1 — Modified Censor output of repetitive elements identified in Aeafru intronic regions. The identified repetitive elements greatly vary in length and among them the most abundant are the NON-LTR/Jockey LINE-1_AA element [86], detected in 38 copies, and the NON-LTR/SINE Feilai elements [87], detected in 36 copies. (PDF) [file pone.0048554.s008.pdf]

Table S1 - Modified Censor output of repetitive elements identified in *Aeafru* intronic regions

Aeafru Intron	Name	Class	From	To	Score
1	AeBuster3	DNA/hAT	375736	375807	293
2	AeHerves2	DNA/hAT	7775	7827	253
1	Ag-Outcast-6	NonLTR/Outcast	227962	227997	206
1	AMPLICON_AA	NonLTR/RTE	415303	415359	249
5	AMPLICON_AA	NonLTR/RTE	17149	17469	1135
1	ATCOPIA30_I	LTR/Copia	339909	340002	221
1	ATENSPM5	DNA/EnSpm	372385	372427	225
1	ATLINE1_6	NonLTR/L1	410542	410622	250
1	ATREP20	DNA/Helitron	409738	409816	211
2	BEL-10_AA-I	LTR/BEL	3153	3985	1083
2	BEL-10_AA-I	LTR/BEL	3986	4120	384
2	BEL-10_AA-I	LTR/BEL	4211	4430	933
2	BEL-10_AA-I	LTR/BEL	4858	4912	219
1	BEL-10_AA-LTR	LTR/BEL	5113	5191	269
2	BEL-10_AA-LTR	LTR/BEL	1672	1935	862
2	BEL-10_AA-LTR	LTR/BEL	4431	4694	862
1	BEL-172_AA-I	LTR/BEL	263741	263776	236
1	BEL-176_AA-I	LTR/BEL	246110	246277	676
1	BEL-182_AA-I	LTR/BEL	135210	135387	611
1	BEL-208_AA-I	LTR/BEL	187342	187420	252
1	BEL-212_AA-I	LTR/BEL	215733	215762	270
1	BEL-212_AA-I	LTR/BEL	215920	215949	246
1	BEL-22_AA-I	LTR/BEL	135484	135958	951
1	BEL-221_AA-I	LTR/BEL	115989	122455	57971
1	BEL-221_AA-LTR	LTR/BEL	115137	115404	2449
1	BEL-221_AA-LTR	LTR/BEL	115405	115495	788
1	BEL-221_AA-LTR	LTR/BEL	115496	115556	551
1	BEL-221_AA-LTR	LTR/BEL	115912	115988	703
1	BEL-221_AA-LTR	LTR/BEL	122456	122723	2449
1	BEL-221_AA-LTR	LTR/BEL	122724	122814	788
1	BEL-221_AA-LTR	LTR/BEL	122815	122875	551
1	BEL-221_AA-LTR	LTR/BEL	123231	123307	703
1	BEL-228_AA-I	LTR/BEL	53425	53712	1196
1	BEL-228_AA-I	LTR/BEL	132823	133089	1161
1	BEL-233_AA-I	LTR/BEL	134997	135187	538
1	BEL-243_AA-I	LTR/BEL	246278	246800	986
1	BEL-26_CQ-I	LTR/BEL	328298	328356	229
6	BEL-31_CQ-LTR	LTR/BEL	35287	35335	248
1	BEL-32_AA-I	LTR/BEL	237412	237514	307
6	BEL-32_AA-I	LTR/BEL	17145	17191	254
1	BEL-38-I_DR	LTR/BEL	234740	234807	220
1	BEL-48_AA-I	LTR/BEL	7021	7224	699
1	BEL-48_AA-I	LTR/BEL	8045	8160	537
1	BEL-48_AA-I	LTR/BEL	26533	26942	1265
5	BEL-52_AA-I	LTR/BEL	1313	1399	237
1	BEL-54_AA-I	LTR/BEL	102251	103474	7552
1	BEL-58_AA-I	LTR/BEL	98984	99023	221
1	BEL-58_AA-I	LTR/BEL	257550	257620	289
4	BEL-608_AA-I	LTR/BEL	5323	5510	1520
4	BEL-608_AA-I	LTR/BEL	6914	6961	416
1	BEL-613_AA-I	LTR/BEL	134744	134848	292
1	BEL-621_AA-I	LTR/BEL	136016	136194	483
1	BEL-69_AA-I	LTR/BEL	294632	295324	4776
1	BEL-69_AA-I	LTR/BEL	295325	295358	249
1	BEL-6-I_XT	LTR/BEL	155645	155694	235
1	BEL-7_AA-I	LTR/BEL	190011	190333	1938
1	BEL-7_AA-I	LTR/BEL	190499	190573	495

1	BEL-81_AA-LTR	LTR/BEL	151010	151077	390
1	BEL-8-I_NV	LTR/BEL	237871	237929	222
1	BEL-91_AA-I	LTR/BEL	85367	85472	430
1	BEL-91_AA-I	LTR/BEL	232090	232201	619
1	BEL-91_AA-I	LTR/BEL	406611	406666	211
5	BRODYAGA2	DNA	350	439	204
1	CER15-1-I_CE	LTR	163034	163634	600
1	CER15-1-I_CE	LTR	163682	164118	325
5	Chapaev-1_CE	DNA/Chapaev	2651	2779	252
4	Chapaev-16_HM	DNA/Chapaev	2422	2479	234
1	Chapaev3-1_AA	DNA/Chapaev	154374	154686	784
1	Chapaev3-1N1_AAe	DNA/Chapaev	313890	315022	10407
1	Chapaev-9_HM	DNA/Chapaev	175805	175865	220
1	Chapaev-N1_AAe	DNA/Chapaev	270048	270169	237
1	Chapaev-N1_AAe	DNA/Chapaev	383939	384070	800
1	Chapaev-N2_AAe	DNA/Chapaev	156815	156918	778
1	Chapaev-N3_AAe	DNA/Chapaev	268915	268969	256
1	Chapaev-N4_AAe	DNA/Chapaev	149775	150041	1986
1	Chapaev-N4_AAe	DNA/Chapaev	220641	220853	900
1	Chapaev-N5_AAe	DNA/Chapaev	91390	91553	364
1	Chapaev-N5_AAe	DNA/Chapaev	91557	91652	261
1	Chapaev-N5_AAe	DNA/Chapaev	248060	248101	225
1	Chapaev-N5_AAe	DNA/Chapaev	378224	378402	447
6	Chapaev-N6_AAe	DNA/Chapaev	3901	4289	1893
1	Chapaev-N7_AAe	DNA/Chapaev	189973	190010	266
6	Chapaev-N7_AAe	DNA/Chapaev	5334	5566	1505
1	Ci000012	Interspersed_Repeat	198697	198752	205
5	COP8_I_MT	LTR/Copia	33894	33925	204
1	Copia-1_DPer-I	LTR/Copia	293306	293385	214
1	Copia-101_AA-I	LTR/Copia	16412	16624	392
1	Copia-107_AA-I	LTR/Copia	1368	1589	289
1	Copia-107_AA-I	LTR/Copia	81033	81154	771
1	Copia-107_AA-I	LTR/Copia	168708	168814	337
5	Copia-107_AA-I	LTR/Copia	4187	4249	438
5	Copia-107_AA-I	LTR/Copia	4250	4455	1081
6	Copia-107_AA-I	LTR/Copia	11983	12224	1232
1	Copia-115_AA-LTR	LTR/Copia	69446	69620	1283
1	Copia11-NVi_I	LTR/Copia	270970	271029	227
1	Copia-12_BD-I	LTR/Copia	270829	270888	245
1	Copia-125_AA-I	LTR/Copia	17748	18128	647
1	Copia-125_AA-I	LTR/Copia	310344	310443	749
1	Copia-125_AA-I	LTR/Copia	310444	310631	1319
1	Copia-125_AA-I	LTR/Copia	310926	311554	4738
1	Copia-130_AA-I	LTR/Copia	295861	295889	220
1	Copia-130_AA-I	LTR/Copia	311555	311607	226
1	Copia-133_AA-I	LTR/Copia	16997	17284	780
1	Copia-133_AA-I	LTR/Copia	17352	17728	1021
1	Copia-133_AA-I	LTR/Copia	18135	18311	297
1	Copia-138_AA-I	LTR/Copia	267330	267484	455
1	Copia-14_AA-I	LTR/Copia	266315	266361	245
6	Copia-14_MLP-I	LTR/Copia	17195	17258	262
1	Copia-16_CQ-I	LTR/Copia	303119	303249	265
6	Copia-23_MLP-I	LTR/Copia	5110	5167	234
6	Copia-26_AA-I	LTR/Copia	9573	9794	1764
5	Copia-27_MLP-I	LTR/Copia	45932	46115	336
5	Copia27-VV_LTR	LTR/Copia	2006	2063	240
1	Copia-29_DPu-I	LTR/Copia	126669	126737	223
6	Copia-29-I_VV	LTR/Copia	17037	17076	217
1	Copia-33_SB-I	LTR/Copia	358935	358986	201
1	Copia-34_SB-I	LTR/Copia	401021	401062	219
1	Copia3-I_CR	LTR/Copia	359203	359247	218
6	Copia3-I_DR	LTR/Copia	33744	33780	235
1	Copia-4_AT-I	LTR/Copia	204440	204499	253

1	Copia-41_PIT-I	LTR/Copia	196605	196636	220
1	Copia-43_SB-I	LTR/Copia	317651	317685	219
1	Copia-7_MLP-I	LTR/Copia	127103	127180	243
1	Copia-9_AA-I	LTR/Copia	56086	57280	3241
1	Copia-96_AA-I	LTR/Copia	16836	16945	370
1	Copia-97_AA-I	LTR/Copia	57281	60148	25323
1	Copia-97_AA-LTR	LTR/Copia	55881	56085	1745
1	Copia-97_AA-LTR	LTR/Copia	60149	60368	1865
1	CR1_Ele10	NonLTR/CR1	369325	369363	217
1	CR1_Ele36	NonLTR/CR1	293534	293735	454
1	CR1_Ele42	NonLTR/CR1	28821	28931	399
1	CR1_Ele42	NonLTR/CR1	202366	202477	354
1	CR1_Ele42	NonLTR/CR1	364773	364856	329
5	CR1_Ele42	NonLTR/CR1	3394	3486	299
1	CR1-10_HM	NonLTR/CR1	75765	75830	207
1	CR1-101_AAe	NonLTR/CR1	175890	176201	2446
1	CR1-101_AAe	NonLTR/CR1	176774	178825	17778
1	CR1-101_AAe	NonLTR/CR1	347492	347535	215
6	CR1-11_HM	NonLTR/CR1	2463	2593	252
1	CR1-110_AAe	NonLTR/CR1	349991	350436	2864
6	CR1-18_HM	NonLTR/CR1	12341	12452	215
6	CR1-21_HM	NonLTR/CR1	3422	3534	233
6	CR1-23_SP	NonLTR/CR1	20512	20617	224
1	CR1-4_HM	NonLTR/CR1	385617	385682	249
6	CR1-4_HM	NonLTR/CR1	4886	4951	228
6	CR1-48_HM	NonLTR/CR1	33877	33947	213
1	CR1-52_AAe	NonLTR/CR1	98747	98876	334
4	CR1-52_HM	NonLTR/CR1	6757	6837	232
5	CR1-60_HM	NonLTR/CR1	43017	43075	230
1	CR1-61_HM	NonLTR/CR1	377416	377471	226
1	CR1-86_AAe	NonLTR/CR1	214597	214939	3015
1	CR1-98_AAe	NonLTR/CR1	89929	89995	266
1	CR1-98_AAe	NonLTR/CR1	282735	282877	1271
1	CR1-98_AAe	NonLTR/CR1	325324	325393	308
1	CR1-98_AAe	NonLTR/CR1	415417	415543	291
1	Crack-4_AAe	NonLTR/Crack	411519	411578	231
1	Crack-7_BF	NonLTR/Crack	257958	258081	277
1	Daphne-1_NV	NonLTR/Daphne	413152	413188	223
1	DIRS-1_DPu	LTR/DIRS	87416	87479	220
6	DIRS-18_XT	LTR/DIRS	26328	26405	255
1	DNA-1_AAe	DNA	30260	30455	587
1	DNA-1_AAe	DNA	287304	287373	325
1	DNA-1_AAe	DNA	343048	343216	323
6	DNA-1_AAe	DNA	22868	22988	408
6	DNA-1_AAe	DNA	22989	23134	742
1	DNA-1_CQ	DNA	296851	296950	231
1	DNA-10_AAe	DNA	258683	258887	1174
1	DNA-11_AAe	DNA	341080	341566	3432
1	DNA-12_AAe	DNA	151694	151916	1914
1	DNA-12_AAe	DNA	205065	205317	1875
1	DNA-12_AAe	DNA	362860	363078	1022
1	DNA-12_AAe	DNA	394849	395105	1357
1	DNA-13_AAe	DNA	133645	133847	329
1	DNA-13_AAe	DNA	155202	155323	522
4	DNA-13_AAe	DNA	5518	6273	4201
1	DNA-14_AAe	DNA	225915	226106	615
1	DNA-14_AAe	DNA	226335	226420	240
1	DNA-2_AAe	DNA	38817	38996	363
1	DNA-2_AAe	DNA	79377	79685	2401
1	DNA-2_AAe	DNA	83597	83865	1875
1	DNA-2_AAe	DNA	151920	152022	630
1	DNA-2_AAe	DNA	157401	157586	1081
1	DNA-2_AAe	DNA	158465	158536	379

1	DNA-2_AAe	DNA	231873	231980	258
1	DNA-2_AAe	DNA	232023	232087	237
1	DNA-2_AAe	DNA	366056	366307	1170
4	DNA-2_AAe	DNA	2130	2278	511
6	DNA-2_AAe	DNA	19365	19612	691
1	DNA-2_CQ	DNA	145053	145263	386
1	DNA-2_CQ	DNA	147050	147398	562
1	DNA-2-1_HM	DNA	34874	34918	207
6	DNA-2-24B_DR	DNA	385	464	238
1	DNA-2-3_HM	DNA	392818	392897	203
1	DNA3-1_AAe	DNA	205723	205759	265
1	DNA3-1_AAe	DNA	216044	216127	482
1	DNA3-1_AAe	DNA	220975	221102	915
1	DNA3-1_AAe	DNA	261110	261301	1112
1	DNA3-1_AAe	DNA	410752	410786	292
1	DNA3-1_AAe	DNA	411405	411495	667
1	DNA-5_AAe	DNA	31276	31321	296
1	DNA-5_AAe	DNA	37649	37728	466
1	DNA-5_AAe	DNA	47327	47580	708
1	DNA-5_AAe	DNA	81590	81835	1117
1	DNA-5_AAe	DNA	105819	105847	217
1	DNA-5_AAe	DNA	105894	106086	449
1	DNA-5_AAe	DNA	110805	110921	455
1	DNA-5_AAe	DNA	167625	168110	3282
1	DNA-5_AAe	DNA	168114	168165	259
1	DNA-5_AAe	DNA	168166	168360	1086
1	DNA-5_AAe	DNA	168368	168427	365
1	DNA-5_AAe	DNA	169175	169433	1963
1	DNA-5_AAe	DNA	228435	228487	289
1	DNA-5_AAe	DNA	255673	255735	315
1	DNA-5_AAe	DNA	255798	255843	233
1	DNA-5_AAe	DNA	262651	262829	595
1	DNA-5_AAe	DNA	335066	335110	259
1	DNA-5_AAe	DNA	375363	375579	408
1	DNA-5_AAe	DNA	422356	422579	363
5	DNA-5_AAe	DNA	4457	4699	620
5	DNA-5_AAe	DNA	20147	20400	637
5	DNA-5_AAe	DNA	36803	36885	461
5	DNA-5_AAe	DNA	39491	39976	1840
5	DNA-5_AAe	DNA	41231	41304	334
5	DNA-5_AAe	DNA	41318	41396	457
1	DNA5-1_AAe	DNA	157134	157309	485
1	DNA5-1_AAe	DNA	216308	216609	1557
1	DNA5-1_AAe	DNA	216623	216736	838
1	DNA5-1_AAe	DNA	391818	392213	1721
1	DNA5-1_AAe	DNA	398366	398492	211
6	DNA5-1_AAe	DNA	4290	4442	728
6	DNA5-1_AAe	DNA	4490	4556	413
1	DNA-6_AAe	DNA	168437	168598	522
1	DNA-6_AAe	DNA	311941	312098	373
1	DNA-6_AAe	DNA	364368	364489	510
1	DNA-6-N6_DR	DNA	230292	230394	238
1	DNA-7_AAe	DNA	15695	16014	1328
1	DNA-7_AAe	DNA	186933	187005	373
5	DNA-7_AAe	DNA	34972	35414	2454
6	DNA-7_AAe	DNA	31564	32008	2439
1	DNA7-1_AAe	DNA	37300	37577	2066
1	DNA7-1_AAe	DNA	145587	145687	288
1	DNA7-1_AAe	DNA	146581	146717	292
1	DNA7-1_AAe	DNA	241227	241520	2334
1	DNA7-1_AAe	DNA	364254	364367	922
1	DNA-8_AAe	DNA	23816	23920	296
5	DNA8-1_AAe	DNA	9161	9260	645

6	DNA8-1_AAe	DNA	18937	19063	543
1	DNA8-101_AP	DNA	148122	148153	214
1	DNA8-11_CQ	DNA	205760	205846	416
1	DNA8-2_AAe	DNA	207465	207573	472
1	DNA8-2_AAe	DNA	207688	207935	891
1	DNA8-2_AAe	DNA	209913	210272	1467
1	DNA8-2_AAe	DNA	327155	327239	242
6	DNA8-7_DR	DNA	18458	18500	212
1	DNA8-8_CQ	DNA	48545	48611	223
1	DNA8-8_CQ	DNA	367814	367850	237
5	DNA8-8_CQ	DNA	11650	11712	254
1	DNA8-9_CQ	DNA	126173	126231	249
1	DNA-9_CQ	DNA	404627	404690	217
1	DNA-9-1A_SBi	DNA	267677	267743	214
1	DNA9-2_AAe	DNA	52585	52644	214
1	DNA9-2_AAe	DNA	377259	377362	575
1	DNA9NNN1_DR	DNA	142460	142511	205
1	DNA-TA-1_AAe	DNA	200908	200999	320
1	DNA-TA-1_AAe	DNA	341019	341063	244
1	DNA-TA-1_AAe	DNA	341654	341683	240
1	DNA-TA-3_CQ	DNA	405583	405681	400
1	DNA-TA-4_AAe	DNA	149674	149774	284
1	DNA-TA-4_AAe	DNA	350603	351207	3875
1	DNA-TA-4_CQ	DNA	85610	85717	364
1	DNA-TA-6_AAe	DNA	18695	18895	629
1	DNA-TA-6_AAe	DNA	92408	92601	470
1	DNA-TA-6_AAe	DNA	207019	207128	290
1	DNA-TA-6_AAe	DNA	231213	231465	1134
1	DNA-TA-6_AAe	DNA	232306	232338	220
1	DNA-TA-6_AAe	DNA	233684	233893	936
1	DNA-TA-6_AAe	DNA	297089	297160	287
1	DNA-TA-6_AAe	DNA	403135	403375	684
5	DNA-TA-6_AAe	DNA	29556	29594	230
1	DNA-TA-6_CQ	DNA	134016	134116	245
1	DNA-TA-7_AAe	DNA	47662	47775	318
1	DNA-TA-7_AAe	DNA	106157	106276	317
1	DNA-TA-7_AAe	DNA	143973	144122	258
1	DNA-TA-7_AAe	DNA	422128	422267	368
5	DNA-TA-7_AAe	DNA	9354	9411	270
5	DNA-TA-7_AAe	DNA	20480	20607	397
1	DNA-TA-8_AAe	DNA	226467	226556	397
1	DNA-TA-8_AAe	DNA	226761	226804	253
1	DNA-TA-8_AAe	DNA	402913	403050	962
1	DNA-TA-8_AAe	DNA	420569	420662	602
1	DNA-TTAA-5_NV	DNA	259247	259307	211
1	DNAX-7_SP	DNA	53837	53923	253
1	EnSpm-1_AA	DNA	34675	34834	499
1	EnSpm-1_AA	DNA	216188	216280	273
1	EnSpm-1_AA	DNA	297172	297225	228
1	EnSpm-1_AA	DNA	302057	302209	658
1	EnSpm-1_AA	DNA	390590	390639	239
5	EnSpm-1_AA	DNA	12583	12646	420
5	EnSpm-1_AA	DNA	26416	26508	239
6	EnSpm-1_AA	DNA	13660	13693	280
6	EnSpm-1_AA	DNA	13699	13932	788
6	EnSpm-1_AA	DNA	17638	17799	616
6	EnSpm-1_AA	DNA	17813	17883	366
6	EnSpm-1_AA	DNA	22730	22808	253
6	EnSpm-1_AA	DNA	23135	23203	316
1	EnSpm-1_HV	DNA/EnSpm	248931	249003	243
1	ENSPM2_VV	DNA/EnSpm	325510	325557	201
1	ENSPM2_VV	DNA/EnSpm	367357	367540	213
1	EnSpm-22_SBi	DNA/EnSpm	164350	164559	324

1	EnSpm-3_HM	DNA/EnSpm	300863	300934	207
1	EnSpm-4_HM	DNA/EnSpm	419502	419540	203
1	EnSpm-6_VV	DNA/EnSpm	265188	265246	209
1	EnSpm-6_VV	DNA/EnSpm	340235	340336	202
1	EnSpm-N1_AT	DNA/EnSpm	269730	269794	234
1	EnSpm-N17_SBi	DNA/EnSpm	270411	270503	264
1	EnSpm-N37_SBi	DNA/EnSpm	33233	33301	216
6	ERE2_EH	Interspersed_Repeat	36820	36903	220
6	ERE2_EH	Interspersed_Repeat	38623	38724	245
1	ERV1-N7-I_DR	ERV/ERV1	153187	153246	219
1	ERV2_MD_I	ERV/ERV1	378446	378488	215
1	ERV2-1N-OP_I	ERV/ERV2	410811	410866	229
1	ERV2-5_Pca-LTR	ERV/ERV2	98592	98656	212
1	FEILAI_AA	NonLTR/SINE	64277	64321	307
1	FEILAI_AA	NonLTR/SINE	65109	65185	588
1	FEILAI_AA	NonLTR/SINE	151645	151693	446
1	FEILAI_AA	NonLTR/SINE	154708	154816	456
1	FEILAI_AA	NonLTR/SINE	203357	203460	825
1	FEILAI_AA	NonLTR/SINE	391187	391306	583
5	FEILAI_AA	NonLTR/SINE	39985	40025	237
5	FEILAI_AA	NonLTR/SINE	40075	40343	1946
6	FEILAI_AA	NonLTR/SINE	9233	9300	478
1	FEILAI_B	NonLTR/SINE/SINE2	38423	38699	2321
1	FEILAI_B	NonLTR/SINE/SINE2	42000	42282	2359
1	FEILAI_B	NonLTR/SINE/SINE2	128786	129070	2254
1	FEILAI_B	NonLTR/SINE/SINE2	145731	145924	962
1	FEILAI_B	NonLTR/SINE/SINE2	179295	179421	857
1	FEILAI_B	NonLTR/SINE/SINE2	187493	187772	2063
1	FEILAI_B	NonLTR/SINE/SINE2	199760	200000	1874
1	FEILAI_B	NonLTR/SINE/SINE2	200639	200907	2129
1	FEILAI_B	NonLTR/SINE/SINE2	225088	225235	1111
1	FEILAI_B	NonLTR/SINE/SINE2	225745	225878	977
1	FEILAI_B	NonLTR/SINE/SINE2	238703	238988	2084
1	FEILAI_B	NonLTR/SINE/SINE2	258472	258681	1769
1	FEILAI_B	NonLTR/SINE/SINE2	272160	272443	2390
1	FEILAI_B	NonLTR/SINE/SINE2	310636	310920	2601
1	FEILAI_B	NonLTR/SINE/SINE2	364092	364238	1072
1	FEILAI_B	NonLTR/SINE/SINE2	391437	391542	504
1	FEILAI_B	NonLTR/SINE/SINE2	399501	399768	1633
5	FEILAI_B	NonLTR/SINE/SINE2	10079	10363	2122
1	FEILAI-1B_AAe	NonLTR/SINE	50392	50678	2214
1	FEILAI-1B_AAe	NonLTR/SINE	155374	155447	654
1	FEILAI-1B_AAe	NonLTR/SINE	250243	250522	2133
1	FEILAI-1B_AAe	NonLTR/SINE	256170	256340	999
1	FEILAI-1B_AAe	NonLTR/SINE	294301	294398	388
1	FEILAI-1B_AAe	NonLTR/SINE	298673	298956	2500
1	FEILAI-1B_AAe	NonLTR/SINE	307440	307721	2357
1	FEILAI-1B_AAe	NonLTR/SINE	325658	325780	765
5	FEILAI-1B_AAe	NonLTR/SINE	21642	21923	2201
1	Gecko	NonLTR/SINE/SINE2	65210	65397	1504
1	Gecko	NonLTR/SINE/SINE2	402008	402126	827
5	Gecko	NonLTR/SINE/SINE2	4905	5086	757
5	Gecko	NonLTR/SINE/SINE2	40035	40074	321
6	Ginger2-1_TS	DNA/Ginger2	14024	14080	206
6	Gyp_LTR_MT	LTR/Gypsy	8998	9060	206
5	Gypsy-10_ES-I	LTR/Gypsy	41693	41762	221
1	Gypsy-111_AA-I	LTR/Gypsy	133331	133376	255
1	Gypsy-111_AA-I	LTR/Gypsy	158931	158974	212
1	Gypsy-111_AA-I	LTR/Gypsy	340367	340511	419
1	Gypsy-111_AA-I	LTR/Gypsy	340853	340898	237
1	Gypsy-111_AA-I	LTR/Gypsy	415562	416363	4262
4	Gypsy-111_AA-I	LTR/Gypsy	4951	5017	276
5	Gypsy-111_AA-I	LTR/Gypsy	12773	12991	772

1	Gypsy-115_AA-I	LTR/Gypsy	78954	79210	2068
1	Gypsy-115_AA-I	LTR/Gypsy	103952	104212	2010
1	Gypsy-115_AA-I	LTR/Gypsy	244360	244614	1960
1	Gypsy13-I_SP	LTR/Gypsy	79944	79988	233
1	Gypsy-14N_PPa-LTR	LTR/Gypsy	27844	27957	253
1	Gypsy-14N_PPa-LTR	LTR/Gypsy	217391	217507	210
1	Gypsy-154_AA-I	LTR/Gypsy	25105	25449	804
1	Gypsy-154_AA-I	LTR/Gypsy	25571	25674	503
1	Gypsy-154_AA-I	LTR/Gypsy	260270	260375	288
1	Gypsy-154_AA-I	LTR/Gypsy	281617	281739	933
1	Gypsy-154_AA-I	LTR/Gypsy	282878	283161	1742
6	Gypsy156-LTR_DR	LTR/Gypsy	25817	25883	235
1	GYPSY16-I_AG	LTR/Gypsy	345617	345720	222
1	Gypsy16-I_Dya	LTR/Gypsy	297608	297670	221
1	Gypsy-181_AA-I	LTR/Gypsy	161762	161810	304
5	Gypsy-188_AA-LTR	LTR/Gypsy	31518	31609	249
1	Gypsy-18-I_NVi	LTR/Gypsy	315885	315946	201
6	Gypsy-19_DWil-I	LTR/Gypsy	38575	38603	214
1	Gypsy-197_AA-I	LTR/Gypsy	188035	189044	8125
1	Gypsy-2_OD-I	LTR/Gypsy	73087	73162	239
1	Gypsy-20_OD-I	LTR/Gypsy	33542	33584	200
1	Gypsy-21_MLP-I	LTR/Gypsy	130448	130501	226
5	Gypsy-21-I_DR	LTR/Gypsy	30630	30679	252
1	Gypsy-220_AA-LTR	LTR/Gypsy	332450	332542	301
1	Gypsy-226_AA-I	LTR/Gypsy	33889	33951	340
1	Gypsy-227_AA-I	LTR/Gypsy	111311	111751	1388
1	Gypsy-23_CQ-I	LTR/Gypsy	343753	343795	229
1	Gypsy-23_OD-I	LTR/Gypsy	333863	333924	234
5	Gypsy-243_AA-I	LTR/Gypsy	25159	25275	214
1	Gypsy-245_AA-LTR	LTR/Gypsy	84336	84405	305
1	Gypsy-247_AA-I	LTR/Gypsy	226807	226886	227
1	Gypsy-255_AA-I	LTR/Gypsy	257682	257753	351
1	Gypsy-255_AA-I	LTR/Gypsy	258138	258227	394
1	Gypsy-255_AA-I	LTR/Gypsy	261786	261925	549
1	Gypsy-255_AA-I	LTR/Gypsy	261931	262138	1518
1	Gypsy-255_AA-I	LTR/Gypsy	312437	312580	911
1	Gypsy-256_AA-I	LTR/Gypsy	206709	206951	1327
1	Gypsy-257_AA-I	LTR/Gypsy	422063	422121	336
5	Gypsy-257_AA-I	LTR/Gypsy	20625	20812	1342
1	Gypsy-258_AA-I	LTR/Gypsy	133231	133313	612
4	Gypsy-258_AA-I	LTR/Gypsy	2850	2914	383
6	Gypsy-258_AA-I	LTR/Gypsy	15147	15261	838
1	Gypsy-259_AA-I	LTR/Gypsy	260418	260544	860
1	Gypsy-259_AA-I	LTR/Gypsy	412438	412608	427
6	Gypsy-259_AA-I	LTR/Gypsy	14900	14962	372
6	Gypsy-26_DPu-I	LTR/Gypsy	26985	27049	263
1	Gypsy-29_VV-I	LTR/Gypsy	75964	75997	200
1	Gypsy2-I_Dmoj	LTR/Gypsy	167070	167318	217
1	Gypsy3_MH-I	LTR/Gypsy	318330	318360	214
1	Gypsy-3_PPM-I	LTR/Gypsy	98195	98229	222
6	Gypsy-38_DPu-I	LTR/Gypsy	21175	21249	210
1	Gypsy-45_BD-I	LTR/Gypsy	406169	406212	249
1	GYPSY49-I_AG	LTR/Gypsy	361881	361943	208
1	Gypsy4-SM_I	LTR/Gypsy	82786	82900	216
6	Gypsy-54_AA-LTR	LTR/Gypsy	30645	30690	223
1	Gypsy-589_AA-I	LTR/Gypsy	315513	315571	217
1	Gypsy-594_AA-I	LTR/Gypsy	47788	47990	1676
1	Gypsy-594_AA-I	LTR/Gypsy	106277	106489	1447
1	Gypsy-597_AA-I	LTR/Gypsy	244740	244786	230
1	Gypsy-597_AA-I	LTR/Gypsy	245216	245269	233
1	Gypsy-597_AA-LTR	LTR/Gypsy	380898	381460	5031
5	Gypsy-65_AA-LTR	LTR/Gypsy	24725	24809	328
1	Gypsy6-I_AP	LTR/Gypsy	268688	268742	211

1	Gypsy6-NVi_I	LTR/Gypsy	142666	142705	207
6	Gypsy-71_CQ-I	LTR/Gypsy	21719	21750	212
1	Gypsy-86_AA-I	LTR/Gypsy	390689	390912	883
1	Gypsy-88_CQ-I	LTR/Gypsy	204087	204149	311
1	Gypsy-9_OD-I	LTR/Gypsy	267941	268038	205
5	GYPSY9-I_AG	LTR/Gypsy	18328	18396	208
1	HAL1-3_Cho	NonLTR/L1	42705	42777	253
1	HAL1-4_Cho	NonLTR/L1	334677	334724	220
6	HAL1B	NonLTR/L1	1981	2018	223
1	HARBINGER3_DR	DNA/Harbinger	269015	269144	217
2	Harbinger-4_BF	DNA/Harbinger	5649	5704	227
1	HARB-N5_Mad	DNA/Harbinger	25512	25555	232
1	hAT-10_XT	DNA/hAT	184395	184468	272
5	hAT-12_XT	DNA/hAT	13173	13227	262
1	hAT-13_HM	DNA/hAT	337348	337417	222
1	hAT-15_SM	DNA/hAT	35061	35165	235
6	hAT-2_NV	DNA/hAT	14624	14674	230
1	hAT-28_HM	DNA/hAT	66962	67058	237
1	HAT3_CB	DNA/hAT	328372	328508	312
1	hAT5-3_NV	DNA/hAT	175089	175142	234
6	hAT5-3_NV	DNA/hAT	11171	11224	236
5	hAT-58_HM	DNA/hAT	43126	43191	205
1	hAT-6_SM	DNA/hAT	373731	373793	205
6	hAT-6_VV	DNA/hAT	21945	22028	211
1	hAT-63_HM	DNA/hAT	169603	169666	275
6	hAT-68_HM	DNA/hAT	39255	39315	204
1	hAT-7_AP	DNA/hAT	42359	42399	221
1	hAT-72_HM	DNA/hAT	1143	1267	236
1	hAT-81_HM	DNA/hAT	319455	319506	238
6	hAT-83_HMa	DNA/hAT	9093	9165	291
1	hATm-1_HM	DNA/hAT	35554	35611	200
1	hATm-38_HM	DNA/hAT	264771	264808	221
1	hATm-47_HM	DNA/hAT	199315	199372	234
1	hAT-N1B_CQ	DNA/hAT	157605	157655	305
1	hAT-N22_DR	DNA/hAT	328636	329141	501
1	hATx-10_SM	DNA/hAT	42617	42654	218
1	HELITRON1_CE	DNA/Helitron	2024	2081	239
5	HELITRON1_CE	DNA/Helitron	34577	34650	246
1	Helitron-2_AAe	DNA/Helitron	112318	112347	234
1	Helitron-2_AAe	DNA/Helitron	112857	113864	8064
1	Helitron2_PPa	DNA/Helitron	32456	32509	202
1	HELITRON7_CB	DNA/Helitron	317101	317289	249
5	HELITRON7_CB	DNA/Helitron	43983	44445	447
5	HELITRON7_CB	DNA/Helitron	44688	45170	337
5	HELITRON7_CB	DNA/Helitron	45345	45712	274
6	HELITRON9A_CB	DNA/Helitron	3536	3621	205
1	HELITRONY1E	DNA/Helitron	224331	224384	213
5	I_Ele18	NonLTR/I	34675	34729	451
1	I_Ele20	NonLTR/I	288270	288629	789
1	I_Ele43	NonLTR/I	287539	288269	4267
1	I_Ele8	NonLTR/I	32902	32933	221
1	I-44_AAe	NonLTR/I	269145	269243	722
1	I-44_AAe	NonLTR/I	269244	269552	2364
1	I-44_AAe	NonLTR/I	271471	271640	1241
1	I-44_AAe	NonLTR/I	271641	271803	1182
1	I-44_AAe	NonLTR/I	271804	271935	1039
1	I-50_AAe	NonLTR/I	397954	398000	340
1	I-60_AAe	NonLTR/I	196701	196939	586
1	I-60_AAe	NonLTR/I	225470	225723	679
1	I-76_AAe	NonLTR/I	410019	410065	249
1	I-7B_AAe	NonLTR/I	334300	334479	1088
1	I-7B_AAe	NonLTR/I	334481	334577	455
5	I-7B_AAe	NonLTR/I	25962	26063	413

1	I-7C_AAe	NonLTR/I	312614	313578	5916
1	I-80B_AAe	NonLTR/I	108040	108524	2636
1	IAPEYI	ERV/ERV2	214533	214569	230
6	IAPLTR4_I	ERV/ERV2	19619	19663	213
6	IBGYPSY1_I	LTR/Gypsy	38470	38546	221
1	ID_B1	NonLTR/SINE	382313	382367	253
1	INVADER3_I	LTR/Gypsy	411241	411280	204
1	ITmD37D_Ele1	DNA/Mariner	90667	90833	654
1	ITmD37D_Ele1	DNA/Mariner	90837	91380	1744
1	ITmD37D_Ele1	DNA/Mariner	91660	91757	501
1	ITmD37D_Ele3	DNA/Mariner	227320	227410	205
1	ITmD37D_Ele3	DNA/Mariner	227565	227723	218
1	ITmD37D_Ele7	DNA/Mariner	413942	414065	292
1	ITmD37E_Ele11	DNA/Mariner	128544	128667	279
1	ITmD37E_Ele9	DNA/Mariner	260586	260678	309
1	JAM1	NonLTR/RTE	62990	63447	1988
1	JAM1	NonLTR/RTE	63454	63609	619
1	JAM1	NonLTR/RTE	170148	170433	2276
1	JAM1	NonLTR/RTE	170434	171038	4861
1	JAM1	NonLTR/RTE	171050	172843	14523
1	JAM1	NonLTR/RTE	172854	173065	1762
1	JAM1	NonLTR/RTE	263867	263954	478
1	JAM1	NonLTR/RTE	300083	300379	1500
1	JAM1B_AAe	NonLTR/RTE	67357	67443	265
1	JAM1B_AAe	NonLTR/RTE	134600	134711	396
1	JAM1C_AAe	NonLTR/RTE	39680	39998	1946
1	JAM1C_AAe	NonLTR/RTE	93361	94001	4457
1	JAM1C_AAe	NonLTR/RTE	94407	94566	1037
1	JAM1C_AAe	NonLTR/RTE	94567	96029	9892
1	JAM1C_AAe	NonLTR/RTE	96030	96072	308
5	JAM1C_AAe	NonLTR/RTE	28788	28852	337
1	Jockey_Ele7	NonLTR/Jockey	113900	114054	451
1	Jockey_Ele7	NonLTR/Jockey	114403	114585	432
1	Jockey_Ele7	NonLTR/Jockey	114592	114624	209
1	Jockey_Ele7	NonLTR/Jockey	114975	115059	449
1	Jockey_Ele7	NonLTR/Jockey	125512	125561	312
1	Jockey-11_AAe	NonLTR/Jockey	115060	115136	235
1	Jockey-11_AAe	NonLTR/Jockey	123375	123463	399
1	Jockey-11_AAe	NonLTR/Jockey	123464	123584	706
1	Jockey-11_AAe	NonLTR/Jockey	123585	124319	4006
1	Kiri-1_AAe	NonLTR/Kiri	210350	210440	221
1	Kiri-11_CQ	NonLTR/Kiri	53997	54882	2510
1	Kiri-11_CQ	NonLTR/Kiri	54894	55853	2618
1	Kiri-11_CQ	NonLTR/Kiri	60375	61203	1662
1	Kiri-26_AAe	NonLTR/Kiri	51778	51897	313
5	Kiri-35_AAe	NonLTR/Kiri	35677	36685	8925
1	Kiri-6_CQ	NonLTR/Kiri	51991	52211	457
1	Kolobok-13_HM	DNA/Kolobok	154227	154276	209
1	Kolobok-16_HM	DNA/Kolobok	229867	229911	214
6	L1_Ele13	NonLTR/L1	23394	23484	454
6	L1_Ele13	NonLTR/L1	23485	23537	273
1	L1_RS1_5end	NonLTR/L1	219362	219411	220
1	L1-1_ET	NonLTR/L1	339405	339442	212
1	L1-3_NV	NonLTR/L1	180442	180511	240
1	L1-43_AAe	NonLTR/L1	393005	393175	961
6	L1-5_MD	NonLTR/L1	36308	36351	222
6	L1MC4_5end	NonLTR/L1	38981	39033	201
1	L2B-1_HM	NonLTR/L2B	143812	143899	201
1	L2B-2_AAe	NonLTR/L2B	363438	363485	205
1	Lian-Aa1	NonLTR/Loa	76552	76658	542
1	Lian-Aa1	NonLTR/Loa	76659	76903	1043
1	Lian-Aa1	NonLTR/Loa	80884	80985	354
1	Lian-Aa1	NonLTR/Loa	86747	86970	760

1	Lian-Aa1	NonLTR/Loa	304802	305153	3065
1	Lian-Aa1	NonLTR/Loa	317854	317912	328
1	Lian-Aa1	NonLTR/Loa	327900	328056	1387
5	Lian-Aa1	NonLTR/Loa	27510	27569	432
5	Lian-Aa1	NonLTR/Loa	27588	27748	547
5	Lian-Aa1	NonLTR/Loa	27764	27935	597
5	Lian-Aa1	NonLTR/Loa	27936	28002	280
5	Lian-Aa1	NonLTR/Loa	28028	28061	267
5	Lian-Aa1	NonLTR/Loa	28077	28276	692
1	LINE-1_AA	NonLTR/Jockey	14847	15687	7081
1	LINE-1_AA	NonLTR/Jockey	50214	50314	357
1	LINE-1_AA	NonLTR/Jockey	50321	50387	327
1	LINE-1_AA	NonLTR/Jockey	50679	50888	1000
1	LINE-1_AA	NonLTR/Jockey	50895	51082	1178
1	LINE-1_AA	NonLTR/Jockey	81952	82059	464
1	LINE-1_AA	NonLTR/Jockey	100305	100393	543
1	LINE-1_AA	NonLTR/Jockey	103832	103951	532
1	LINE-1_AA	NonLTR/Jockey	104213	104954	3793
1	LINE-1_AA	NonLTR/Jockey	104995	105818	5306
1	LINE-1_AA	NonLTR/Jockey	106493	106616	755
1	LINE-1_AA	NonLTR/Jockey	111752	111836	392
1	LINE-1_AA	NonLTR/Jockey	204648	205043	2225
1	LINE-1_AA	NonLTR/Jockey	228673	229249	3750
1	LINE-1_AA	NonLTR/Jockey	229291	229732	2582
1	LINE-1_AA	NonLTR/Jockey	250564	252258	12435
1	LINE-1_AA	NonLTR/Jockey	252260	254630	16092
1	LINE-1_AA	NonLTR/Jockey	275233	275771	3062
1	LINE-1_AA	NonLTR/Jockey	275787	275884	365
1	LINE-1_AA	NonLTR/Jockey	303679	304465	4790
1	LINE-1_AA	NonLTR/Jockey	304636	304791	969
1	LINE-1_AA	NonLTR/Jockey	305154	306918	11154
1	LINE-1_AA	NonLTR/Jockey	307324	307439	731
1	LINE-1_AA	NonLTR/Jockey	307722	307807	633
1	LINE-1_AA	NonLTR/Jockey	307810	307867	263
1	LINE-1_AA	NonLTR/Jockey	308934	308992	282
1	LINE-1_AA	NonLTR/Jockey	308998	309056	293
1	LINE-1_AA	NonLTR/Jockey	309057	309490	2930
1	LINE-1_AA	NonLTR/Jockey	352269	353051	5955
1	LINE-1_AA	NonLTR/Jockey	353561	353660	872
1	LINE-1_AA	NonLTR/Jockey	353927	354870	7552
1	LINE-1_AA	NonLTR/Jockey	354871	354974	572
1	LINE-1_AA	NonLTR/Jockey	355036	357611	20790
1	LINE-1_AA	NonLTR/Jockey	387400	387958	3418
1	LINE-1_AA	NonLTR/Jockey	387968	388216	1154
1	LINE-1_AA	NonLTR/Jockey	388219	388620	2888
1	LINE-1_AA	NonLTR/Jockey	407821	407957	1050
1	LINE-1_AA	NonLTR/Jockey	407958	408252	1480
1	LINE1-34_SBi	NonLTR/L1	215824	215863	227
1	LINE1-8_ZM	NonLTR/L1	220005	220062	208
1	LOA_Ele4	NonLTR/Loa	109187	109239	402
1	LOA_Ele6	NonLTR/Loa	7905	8006	292
1	Loner_Ele1	NonLTR/I	82060	82164	511
1	Loner_Ele1	NonLTR/I	83451	83540	575
1	Loner_Ele1	NonLTR/I	83866	83972	723
1	Loner_Ele1	NonLTR/I	232339	233022	4151
1	Loner_Ele1	NonLTR/I	233023	233681	5253
1	LTR43_I	ERV/ERV1	365788	365876	227
1	LTR77-int_TS	ERV	84442	84484	223
1	Mariner-14_HM	DNA/Mariner	407149	407196	222
1	Mariner-3_HM	DNA/Mariner	212020	212179	220
1	Mariner-35_SM	DNA/Mariner	35208	35258	206
6	Mariner-6_HM	DNA/Mariner	26417	26526	252
5	MARINER60_CB	DNA/Mariner	3896	3940	204

1	Mariner-9_HM	DNA/Mariner	206620	206683	205
1	MarinerN-2_AP	DNA/Mariner	276778	276812	224
1	Merlin1_CB	DNA/Merlin	65700	65761	220
1	MITE_AA	Interspersed_Repeat	248294	248438	785
1	MITE_AA	Interspersed_Repeat	248452	248486	256
1	MITE_AA	Interspersed_Repeat	272925	273444	3600
6	MOGWAI1_EI	DNA/Mariner	3295	3377	231
1	MosquI_Aa2	NonLTR/I	417558	417626	411
1	MSAT-1_AAe	Simple/Sat/MSAT	22481	22799	600
1	MSAT-1_AAe	Simple/Sat/MSAT	22838	22981	355
1	MSAT-1_AAe	Simple/Sat/MSAT	22983	23568	1110
1	MSAT-1_AAe	Simple/Sat/MSAT	23588	23744	342
1	MSAT-1_AAe	Simple/Sat/MSAT	67858	67920	200
1	MSAT-1_AAe	Simple/Sat/MSAT	67927	68057	337
1	MSAT-1_AAe	Simple/Sat/MSAT	68258	68529	458
1	MSAT-1_AAe	Simple/Sat/MSAT	68871	69166	460
1	MSAT-1_AAe	Simple/Sat/MSAT	69654	69975	665
1	MSAT-1_AAe	Simple/Sat/MSAT	70228	70703	449
1	MSAT-1_AAe	Simple/Sat/MSAT	70758	70951	331
1	MSAT-1_AAe	Simple/Sat/MSAT	106650	106948	363
1	MSAT-1_AAe	Simple/Sat/MSAT	202215	202287	220
1	MSAT-1_AAe	Simple/Sat/MSAT	203182	203314	379
1	MSAT-1_AAe	Simple/Sat/MSAT	220108	220233	262
1	MSAT-1_AAe	Simple/Sat/MSAT	238443	238675	459
1	MSAT-1_AAe	Simple/Sat/MSAT	239097	239203	258
1	MSAT-1_AAe	Simple/Sat/MSAT	239205	239260	231
1	MSAT-1_AAe	Simple/Sat/MSAT	239295	239869	759
1	MSAT-1_AAe	Simple/Sat/MSAT	239872	239975	277
1	MSAT-1_AAe	Simple/Sat/MSAT	239988	240530	1011
1	MSAT-1_AAe	Simple/Sat/MSAT	240810	241144	665
1	MSAT-1_AAe	Simple/Sat/MSAT	241546	241603	203
1	MSAT-1_AAe	Simple/Sat/MSAT	241633	241975	371
1	MSAT-1_AAe	Simple/Sat/MSAT	241980	242161	241
1	MSAT-1_AAe	Simple/Sat/MSAT	242464	242741	423
1	MSAT-1_AAe	Simple/Sat/MSAT	242842	243401	487
1	MSAT-1_AAe	Simple/Sat/MSAT	256465	256719	293
1	MSAT-1_AAe	Simple/Sat/MSAT	256800	257296	575
1	MSAT-1_AAe	Simple/Sat/MSAT	273496	273592	264
1	MSAT-1_AAe	Simple/Sat/MSAT	316105	316536	634
1	MSAT-1_AAe	Simple/Sat/MSAT	316570	317056	749
1	MSAT-1_AAe	Simple/Sat/MSAT	364908	365132	361
1	MSAT-1_AAe	Simple/Sat/MSAT	369666	370238	926
5	MSAT-1_AAe	Simple/Sat/MSAT	3133	3305	283
5	MSAT-1_AAe	Simple/Sat/MSAT	13976	14132	243
5	MSAT-1_AAe	Simple/Sat/MSAT	34108	34333	318
5	MSAT-1_AAe	Simple/Sat/MSAT	34830	34970	295
5	MSAT-1_AAe	Simple/Sat/MSAT	35451	35539	253
1	MSAT-2_AAe	Simple/Sat/MSAT	217514	217594	237
1	MSAT-2_AAe	Simple/Sat/MSAT	337062	337242	332
1	MSAT-2_AAe	Simple/Sat/MSAT	337477	337592	227
1	MSAT-2_AAe	Simple/Sat/MSAT	337751	337978	629
1	MSAT-2_AAe	Simple/Sat/MSAT	337982	338135	349
1	MSAT-2_AAe	Simple/Sat/MSAT	338145	338420	531
1	MSAT-2_AAe	Simple/Sat/MSAT	373117	373375	422
1	MSAT-2_AAe	Simple/Sat/MSAT	410118	410272	277
1	MSAT-5_AAe	Simple/Sat/MSAT	373427	373615	263
1	mTA_Ele42	DNA	71051	71228	452
1	mTA_Ele42	DNA	89691	89798	356
1	mTA_Ele42	DNA	282541	282702	219
1	mTA_Ele42	DNA	293438	293481	236
1	mTA_Ele42	DNA	403687	403718	220
1	mTA_Ele45	DNA	368745	368906	644
1	mTA_Ele45	DNA	369038	369233	607

1	MuDr-2_HM	DNA/MuDR	394040	394176	219
1	MuDR-2_NV	DNA/MuDR	156785	156814	206
1	MuDR-21_VV	DNA/MuDR	36749	36839	247
1	MuDR3x_AP	DNA/MuDR	208330	208475	219
1	MuDR-7_VV	DNA/MuDR	296300	296352	210
1	MuDR-7_ZM	DNA/MuDR	209609	209692	218
1	Ogre-MT4_LTR	LTR/Gypsy	181569	181604	219
1	Ogre-PT3_LTR	LTR/Gypsy	174184	174261	226
1	ORTE-1_AAe	NonLTR	44296	44508	567
1	ORTE-1_AAe	NonLTR	246889	247300	527
1	ORTE-1_AAe	NonLTR	419689	419746	251
4	ORTE-1_AAe	NonLTR	4241	4298	257
5	ORTE-1_AAe	NonLTR	31743	31855	318
5	ORTE-1_AAe	NonLTR	32213	32326	325
1	ORTE-2_AAe	NonLTR	382041	382131	277
1	ORTE-4_AAe	NonLTR	28616	28673	230
1	ORTE-6_AAe	NonLTR	267219	267320	755
5	OSTE20	DNA	11437	11545	209
1	otherMITEs_Ele11	DNA	320860	321257	2750
1	otherMITEs_Ele11	DNA	321261	321409	1157
6	Outcast-17_AAe	NonLTR/Outcast	34160	34223	239
1	P-1_AAe	DNA/P	27097	27272	675
1	P-1_AAe	DNA/P	27312	27522	844
6	P-1_CR	DNA/P	26815	26858	210
1	P-27_HM	DNA/P	91951	92035	232
1	P-4_AP	DNA/P	367132	367200	217
1	PALTA1_CE	DNA	230417	230476	212
1	Penelope-2_AAe	NonLTR/Penelope	272639	272744	453
1	Penelope-2_CQ	NonLTR/Penelope	66231	66319	274
1	Penelope-3_AAe	NonLTR/Penelope	224056	224148	459
1	Penelope-3_AAe	NonLTR/Penelope	224166	224276	717
1	Penelope-3_AAe	NonLTR/Penelope	244647	244673	256
1	Penelope-8_XT	NonLTR/Penelope	218758	218826	213
1	piggyBac-N1_CQ	DNA/piggyBac	5519	5550	254
1	PIVE	IntegratedVirus/DNAV	389119	389155	203
1	POLINTN1_SM	DNA/Polinton	66744	66803	220
1	Polinton-1_EI	DNA/Polinton	419366	419480	219
6	Polinton1_SM	DNA/Polinton	21784	21837	200
1	Polinton-1_SM	DNA/Polinton	411580	411626	206
1	Polinton-2_CB	DNA/Polinton	51216	51247	212
1	Polinton-2_HM	DNA/Polinton	43111	43176	235
1	Polinton-2_HM	DNA/Polinton	148217	148269	210
1	Polinton-2_HM	DNA/Polinton	198604	198696	209
5	Polinton-2_HM	DNA/Polinton	33325	33395	218
1	Polinton-2A_NV	DNA/Polinton	62504	62543	235
1	Polinton-3_HM	DNA/Polinton	318214	318304	204
5	Polinton-5_NV	DNA/Polinton	1852	1894	260
4	Polinton-9_NVi	DNA/Polinton	1500	1534	212
1	PONY_AA	DNA	39168	39679	3837
1	PONY_AA	DNA	112348	112856	3734
1	PONY_AA	DNA	195939	196439	2736
1	PONY_AA	DNA	353052	353559	3083
1	PONY_AA	DNA	395709	395874	852
1	PONY_AA	DNA	417203	417389	1279
1	R1_Ele1	NonLTR/R1	137082	138647	13008
1	R1_Ele1	NonLTR/R1	190751	195793	39877
1	R1_Ele4	NonLTR/R1	321800	321869	287
2	R1_Ele4	NonLTR/R1	8637	9105	2923
2	R1_Ele4	NonLTR/R1	9111	9735	4396
5	R1_Ele4	NonLTR/R1	22379	22674	1710
5	R1_Ele4	NonLTR/R1	22677	24207	10156
1	R1_Ele7	NonLTR/R1	322324	322683	2800
1	R1_Ele7	NonLTR/R1	322744	323835	7846

1	R1_Ele7	NonLTR/R1	323874	324503	3820
1	R1_Ele8	NonLTR/R1	45768	45854	289
1	R1_Ele8	NonLTR/R1	287404	287512	367
1	R1_Ele8	NonLTR/R1	321912	322322	2140
1	R1_Ele8	NonLTR/R1	322684	322736	255
2	R1_Ele8	NonLTR/R1	6254	6455	968
2	R1_Ele8	NonLTR/R1	8431	8566	386
1	R1_Ele9	NonLTR/R1	329454	329484	217
5	R1_Ele9	NonLTR/R1	20849	21292	1173
5	R1_Ele9	NonLTR/R1	21324	21404	316
5	R1_Ele9	NonLTR/R1	21445	21631	426
5	R1_Ele9	NonLTR/R1	21929	22190	791
5	R1_Ele9	NonLTR/R1	22206	22377	592
1	REP-3_CQ	Interspersed_Repeat	148681	148851	971
1	REP-3_CQ	Interspersed_Repeat	221421	221589	721
1	REP-3_CQ	Interspersed_Repeat	222918	222999	402
1	REP-3_CQ	Interspersed_Repeat	223057	223192	765
1	REP-3_CQ	Interspersed_Repeat	223716	223877	467
1	REP-3_CQ	Interspersed_Repeat	260730	260891	500
1	REP-3_CQ	Interspersed_Repeat	261610	261768	1163
1	REP-5_CQ	Interspersed_Repeat	267852	267895	256
1	REP-5_CQ	Interspersed_Repeat	385370	385506	298
6	RIRE2_I	LTR/Gypsy	15846	15902	233
1	RTE_Ele2	NonLTR/RTE	132709	132740	236
1	RTE_Ele2	NonLTR/RTE	321503	321635	393
2	RTE_Ele2	NonLTR/RTE	523	1668	7004
2	RTE_Ele2	NonLTR/RTE	4956	5048	545
2	RTE_Ele2	NonLTR/RTE	5053	5523	2465
2	RTE_Ele2	NonLTR/RTE	5549	5627	401
2	RTE_Ele2	NonLTR/RTE	5810	5865	316
2	RTE_Ele2	NonLTR/RTE	5896	6119	1619
2	RTE_Ele2	NonLTR/RTE	6183	6242	436
1	RTE_Ele2B_AAe	NonLTR/RTE	39999	40066	315
1	RTE_Ele2B_AAe	NonLTR/RTE	237263	237389	476
1	RTE_Ele2B_AAe	NonLTR/RTE	243408	243601	876
1	RTE_Ele2B_AAe	NonLTR/RTE	320683	320849	571
4	RTE_Ele2B_AAe	NonLTR/RTE	3105	3298	1532
4	RTE_Ele2B_AAe	NonLTR/RTE	3299	3909	4682
4	RTE_Ele2B_AAe	NonLTR/RTE	6962	7628	5441
1	RTE_Ele2C_AAe	NonLTR/RTE	288750	288847	641
1	RTE_Ele2C_AAe	NonLTR/RTE	288850	288919	518
1	RTE_Ele2C_AAe	NonLTR/RTE	288923	289296	2361
1	RTE_Ele4	NonLTR/RTE	417135	417186	247
1	RTE_Ele4	NonLTR/RTE	417397	417538	485
1	RTE_Ele5	NonLTR/RTE	34389	34448	421
1	RTE_Ele5	NonLTR/RTE	38997	39141	615
4	RTE_Ele5	NonLTR/RTE	8069	8173	285
1	SAT-1_AAe	Simple/Sat/SAT	20109	20261	897
1	SAT-1_AAe	Simple/Sat/SAT	20262	20312	351
1	SAT-1_AAe	Simple/Sat/SAT	135969	136015	261
1	SAT-1_AAe	Simple/Sat/SAT	176618	176772	802
1	SAT-1_AAe	Simple/Sat/SAT	281741	281893	1112
1	SAT-1_AAe	Simple/Sat/SAT	281894	282006	695
1	SAT-1_AAe	Simple/Sat/SAT	404333	404442	256
5	SAT-1_AAe	Simple/Sat/SAT	16960	17047	386
1	Shinagawa-1_AAe	DNA	325402	325479	327
6	Shinagawa-1_AAe	DNA	18138	18178	303
1	Shinagawa-10_AAe	DNA	319834	319873	245
1	Shinagawa-10_AAe	DNA	418711	418814	331
1	Shinagawa-11_AAe	DNA	853	888	240
1	Shinagawa-11_AAe	DNA	12761	12849	434
1	Shinagawa-11_AAe	DNA	90298	90375	222
1	Shinagawa-11_AAe	DNA	174604	174701	373

1	Shinagawa-11_AAe	DNA	219092	219160	234
1	Shinagawa-11_AAe	DNA	351217	351281	300
1	Shinagawa-11_AAe	DNA	384431	384499	234
1	Shinagawa-11_AAe	DNA	414648	414999	1249
5	Shinagawa-11_AAe	DNA	14790	14865	499
5	Shinagawa-11_AAe	DNA	16030	16122	645
1	Shinagawa-2_AAe	DNA	384701	384759	284
1	Shinagawa-2_AAe	DNA	421462	421495	229
1	Shinagawa-2_CQ	DNA	277626	277666	258
1	Shinagawa-2_CQ	DNA	278808	278878	289
1	Shinagawa-4_AAe	DNA	112235	112317	279
1	Shinagawa-4_AAe	DNA	267056	267122	290
1	Shinagawa-4_AAe	DNA	297951	298138	711
1	Shinagawa-4_AAe	DNA	313657	313737	263
1	Shinagawa-4_AAe	DNA	329499	329692	1241
6	Shinagawa-4_AAe	DNA	10143	10350	749
1	Shinagawa-6_AAe	DNA	53046	53117	281
1	Shinagawa-6_AAe	DNA	81155	81251	560
1	Shinagawa-6_AAe	DNA	223537	223679	829
1	Shinagawa-6_AAe	DNA	260933	261081	822
1	Shinagawa-6_AAe	DNA	285335	285397	317
1	Shinagawa-6_AAe	DNA	304466	304634	991
1	Shinagawa-6_AAe	DNA	421503	421601	368
5	Shinagawa-6_AAe	DNA	13720	13877	680
6	Shinagawa-6_AAe	DNA	11722	11889	1012
1	Shinagawa-7_AAe	DNA	350459	350601	568
1	Shinagawa-8_AAe	DNA	2838	3027	630
1	Shinagawa-8_AAe	DNA	132225	132310	376
1	Shinagawa-8_AAe	DNA	173506	173671	365
1	Shinagawa-8_AAe	DNA	274351	274553	844
1	Shinagawa-8_AAe	DNA	279048	279093	218
1	Shinagawa-9_AAe	DNA	25022	25101	401
1	Shinagawa-9_AAe	DNA	25680	25818	533
1	Shinagawa-9_AAe	DNA	26193	26261	450
1	Shinagawa-9_AAe	DNA	245270	245391	388
1	Shinagawa-9_AAe	DNA	278592	278637	215
6	Shinagawa-9_AAe	DNA	8068	8216	561
6	Shinagawa-9_AAe	DNA	9424	9571	943
6	Shinagawa-9_AAe	DNA	9795	9983	995
1	SINE2-1_AP	NonLTR/SINE/SINE2	393271	393334	204
1	SINE-4_CQ	NonLTR/SINE	14263	14337	288
1	SINE-4_CQ	NonLTR/SINE	71522	71579	298
5	SMAR4	DNA/Mariner	31369	31396	208
6	Sola1-3_AA	DNA/Sola	18185	18308	503
1	Sola1-4_AA	DNA/Sola	92602	92852	870
1	Sola1-4_AA	DNA/Sola	149276	149527	877
1	Sola1-4_AA	DNA/Sola	185139	185371	727
1	Sola1-4_AA	DNA/Sola	226704	226757	321
1	Sola1-4_AA	DNA/Sola	344716	344791	381
1	Sola1-4_AA	DNA/Sola	344826	344895	281
1	Sola1-4_AA	DNA/Sola	345074	345125	345
1	Sola1-4_AA	DNA/Sola	353668	353926	1921
5	Sola1-4_AA	DNA/Sola	5791	6001	958
1	Sola1-N1_AAe	DNA/Sola	299822	299997	634
1	Sola1-N1_AAe	DNA/Sola	300038	300081	262
1	Sola1-N1_AAe	DNA/Sola	300380	300456	350
5	Sola1-N1_CQ	DNA/Sola	30795	30860	203
1	Sola1-N5_AAe	DNA/Sola	140935	140984	303
1	Sola1-N5_AAe	DNA/Sola	400033	400206	375
1	Sola1-N5_AAe	DNA/Sola	405314	405380	293
5	Sola1-N5_AAe	DNA/Sola	28317	28510	1091
1	Sola2-2_AAe	DNA/Sola	24246	24619	1222
1	Sola2-3N1_SP	DNA/Sola	258358	258419	224

1	Sola2-N1_AAe	DNA/Sola	150044	150224	913
1	Sola2-N1_AAe	DNA/Sola	169704	169993	2148
1	Sola2-N1_AAe	DNA/Sola	169994	170121	1097
1	Sola2-N1_AAe	DNA/Sola	382659	382700	201
1	Sola2-N4_AAe	DNA/Sola	37229	37281	249
1	Sola2-N4_AAe	DNA/Sola	100395	101267	7118
1	Sola2-N4_AAe	DNA/Sola	101268	101299	236
1	Sola2-N4_AAe	DNA/Sola	101325	102019	5738
5	Sola2-N4_AAe	DNA/Sola	42399	42545	421
5	Sola2-N4_AAe	DNA/Sola	42731	42895	840
6	Sola2-N4_AAe	DNA/Sola	13068	13202	474
6	Sola2-N4_AAe	DNA/Sola	32065	32176	326
1	Sola3-1_AA	DNA/Sola	144165	144431	1397
1	Sola3-1_AA	DNA/Sola	412174	412437	1250
5	Sola3-1_AA	DNA/Sola	33573	33839	2107
1	Sola3-1N1_AA	DNA/Sola	10585	10655	421
1	Sola3-1N1_AA	DNA/Sola	12447	12712	1342
1	Sola3-1N1_AA	DNA/Sola	43427	43714	1707
1	Sola3-1N1_AA	DNA/Sola	88644	88705	287
1	Sola3-1N1_AA	DNA/Sola	88706	88880	882
1	Sola3-1N1_AA	DNA/Sola	110639	110779	656
1	Sola3-1N1_AA	DNA/Sola	132363	132664	2057
1	Sola3-1N1_AA	DNA/Sola	179243	179294	208
1	Sola3-1N1_AA	DNA/Sola	203562	203611	262
1	Sola3-1N1_AA	DNA/Sola	205357	205475	298
1	Sola3-1N1_AA	DNA/Sola	215444	215725	2054
1	Sola3-1N1_AA	DNA/Sola	235611	235669	277
1	Sola3-1N1_AA	DNA/Sola	259566	259720	304
1	Sola3-1N1_AA	DNA/Sola	259931	260008	424
1	Sola3-1N1_AA	DNA/Sola	262221	262495	1352
1	Sola3-1N1_AA	DNA/Sola	286841	287117	2072
1	Sola3-1N1_AA	DNA/Sola	294436	294512	246
1	Sola3-1N1_AA	DNA/Sola	358080	358384	2036
1	Sola3-1N1_AA	DNA/Sola	375166	375215	274
4	Sola3-1N1_AA	DNA/Sola	4523	4705	1254
6	Sola3-1N1_AA	DNA/Sola	5635	5916	2282
1	TC1A	DNA/Mariner	339116	339212	242
1	TCN760	Interspersed_Repeat	46300	46406	286
1	TDD4	DNA/Ginger2	5934	6117	225
1	TNR1	DNA	282474	282508	211
1	Transib1_AA	DNA/Transib	184250	184320	486
1	Transib1_AA	DNA/Transib	382994	383079	217
6	Transib1_AA	DNA/Transib	3653	3705	242
1	Transib3_AA	DNA/Transib	44556	44667	375
1	Transib3_AA	DNA/Transib	247395	247455	282
4	Transib3_AA	DNA/Transib	3917	4031	355
1	Transib-6_AAe	DNA/Transib	124500	124592	291
1	Transib-6_AAe	DNA/Transib	124641	125177	1178
1	Transib-6_AAe	DNA/Transib	148374	148486	468
1	Transib-6_AAe	DNA/Transib	148852	148942	520
1	Transib-6_AAe	DNA/Transib	149017	149141	510
1	Transib-6_AAe	DNA/Transib	149640	149673	201
1	Transib-6_AAe	DNA/Transib	181193	181308	241
1	Transib-6_AAe	DNA/Transib	183185	183551	1335
1	Transib-6_AAe	DNA/Transib	269605	269722	375
1	Transib-6_AAe	DNA/Transib	271072	271470	1125
1	Transib-6_AAe	DNA/Transib	283506	284137	3715
1	Transib-6_AAe	DNA/Transib	284188	284347	584
1	Transib-6_AAe	DNA/Transib	285050	285334	1100
1	Transib-6_AAe	DNA/Transib	285483	286175	3686
1	Transib-N1_AAe	DNA/Transib	181677	181738	241
1	Transib-N1_AAe	DNA/Transib	383146	383210	237
1	Transib-N1_CQ	DNA/Transib	31467	31621	460

1	Transib-N1_CQ	DNA/Transib	36383	36537	471
1	TransibN2_AG	DNA/Transib	32935	33000	217
1	Transib-N3_AAe	DNA/Transib	159458	159693	305
1	Transib-N3_AAe	DNA/Transib	180156	180377	535
5	Transib-N3_AAe	DNA/Transib	42224	42331	222
1	TransibN3_AG	DNA/Transib	230085	230181	209
5	TRUNCATOR2_LTR	LTR	32085	32192	328
1	TWIN	NonLTR/SINE	96748	96805	323
1	Tx1-3_CQ	NonLTR/Tx1	219493	219565	217
1	VHARB4_VV	DNA/Harbinger	52782	52821	231
1	VHARB4_VV	DNA/Harbinger	272763	272899	254
1	Waldo-1_AAe	NonLTR/R1	77448	77555	319
1	Waldo-2_AAe	NonLTR/R1	16768	16806	241
1	Waldo-2_AAe	NonLTR/R1	23973	24119	438
1	Waldo-2_AAe	NonLTR/R1	76910	77141	823
1	Waldo-2_AAe	NonLTR/R1	77702	77796	434
1	Waldo-2_AAe	NonLTR/R1	78003	78611	2417
1	Waldo-2_AAe	NonLTR/R1	78627	78941	1683
1	Waldo-2_AAe	NonLTR/R1	79224	79363	586
1	Waldo-2_AAe	NonLTR/R1	79686	79829	746
1	Waldo-2_AAe	NonLTR/R1	80362	80866	2057
1	Waldo-2_AAe	NonLTR/R1	81423	81580	656
1	Waldo-2_AAe	NonLTR/R1	99024	99120	344
1	Waldo-2_AAe	NonLTR/R1	107791	107842	423
1	Waldo-2_AAe	NonLTR/R1	107893	108039	585
1	Waldo-2_AAe	NonLTR/R1	201848	202061	1519
1	Waldo-2_AAe	NonLTR/R1	248103	248200	440
1	Waldo-2_AAe	NonLTR/R1	324857	325249	1856
1	Waldo-2_AAe	NonLTR/R1	329190	329251	507
1	Waldo-2_AAe	NonLTR/R1	329252	329453	1062
1	Waldo-2_AAe	NonLTR/R1	343847	344051	1201
1	Waldo-2_AAe	NonLTR/R1	391333	391433	619
2	Waldo-2_AAe	NonLTR/R1	6665	6728	254
1	Waldo-3_AAe	NonLTR/R1	41779	41867	291
1	Waldo-3_AAe	NonLTR/R1	136393	136539	1005
1	Waldo-3_AAe	NonLTR/R1	136540	136686	795
1	Waldo-3_AAe	NonLTR/R1	136866	137049	1343
1	Waldo-3_AAe	NonLTR/R1	138657	138829	1006
1	Waldo-3_AAe	NonLTR/R1	138833	138875	311
1	Waldo-3_AAe	NonLTR/R1	138876	139230	2629
1	Waldo-3_AAe	NonLTR/R1	139232	139484	2017
1	Waldo-3_AAe	NonLTR/R1	139485	140283	6192
1	Waldo-3_AAe	NonLTR/R1	280877	281025	761
1	Waldo-3_AAe	NonLTR/R1	281026	281251	471
1	Waldo-3_AAe	NonLTR/R1	283174	283312	562
1	Waldo-3_AAe	NonLTR/R1	283316	283505	738
1	Waldo-3_AAe	NonLTR/R1	286205	286451	1103
1	Waldo-3_AAe	NonLTR/R1	286456	286769	1239
1	Waldo-3_AAe	NonLTR/R1	289365	289571	550
1	Waldo-3_AAe	NonLTR/R1	329693	330013	2055
1	Waldo-3_AAe	NonLTR/R1	330015	330150	983
1	Waldo-3_AAe	NonLTR/R1	330154	331074	5920
1	Waldo-3_AAe	NonLTR/R1	331083	331547	3138
1	Waldo-3_AAe	NonLTR/R1	331548	331706	1047
1	Waldo-4_AAe	NonLTR/R1	40217	40417	517
1	Waldo-4_AAe	NonLTR/R1	164564	165186	3194
1	Waldo-4_AAe	NonLTR/R1	165187	166279	6974
1	Waldo-4_AAe	NonLTR/R1	166281	166386	545
1	Waldo-4_AAe	NonLTR/R1	166387	166851	3057
1	Waldo-4_AAe	NonLTR/R1	287210	287293	365
1	Waldo-4_AAe	NonLTR/R1	290929	291198	2018
1	Waldo-4_AAe	NonLTR/R1	291199	291294	452
2	Waldo-4_AAe	NonLTR/R1	316	380	300

2	Waldo-4_AAe	NonLTR/R1	6461	6577	331
2	Waldo-4_AAe	NonLTR/R1	6803	7017	1274
2	Waldo-4_AAe	NonLTR/R1	7076	7772	3225
2	Waldo-4_AAe	NonLTR/R1	7829	8378	2922
1	Waldo-6_AAe	NonLTR/R1	289641	290054	2413
1	Waldo-6_AAe	NonLTR/R1	290065	290716	3982
1	Waldo-6_AAe	NonLTR/R1	290717	290928	1314
1	Waldo-6_AAe	NonLTR/R1	291300	291452	823
1	Waldo-6_AAe	NonLTR/R1	291461	291663	1454
1	Waldo-6_AAe	NonLTR/R1	291678	292276	3529
1	WUJIN	DNA	141006	141074	393
1	WUJIN	DNA	162053	162099	294
1	WUJIN	DNA	349130	349318	1358
1	WUJIN	DNA	406487	406600	721
1	Zator-1_AA	DNA/Zator	173369	173473	615
1	Zator-1_AA	DNA/Zator	175560	175800	1055
1	Zator-1_AA	DNA/Zator	327766	327899	745
1	Zator-1_AA	DNA/Zator	328058	328201	664
5	Zator-1_AA	DNA/Zator	31269	31365	368
1	Zator-1_CP	DNA/Zator	152774	153019	536
1	Zator-1_CP	DNA/Zator	154095	154143	398
1	Zator-1_CP	DNA/Zator	176202	176366	421
1	Zator-1_CP	DNA/Zator	195806	195895	353
1	Zator-1_CP	DNA/Zator	244677	244713	243
1	Zator-1_CP	DNA/Zator	247459	247579	378
1	Zator-1_CP	DNA/Zator	390106	390295	447
1	Zator-N1_AAe	DNA/Zator	190334	190491	541
1	Zator-N1_AAe	DNA/Zator	384117	384280	1257
1	ZEBEDEE	LTR/Copia	10940	11211	1139
1	ZEBEDEE	LTR/Copia	107207	107448	1645
