# Supplementary material for: The Orthologue of the Fruitfly Sex Behaviour Gene Fruitless in the Mosquito Aedes aegypti: Evolution of Genomic Organisation and Alternative Splicing
Source: PLoS One. 2013 Feb 13;8(2):e48554. doi: 10.1371/journal.pone.0048554 (PMC3572092; doi:10.1371/journal.pone.0048554)
Supplement: Table S2 — Ae. aegypti intron analysis. Tabular output of Aedes aegypti intron analysis. The average number of repetitive elements per kb (indicated as NoRE/kb) in Aeafru introns is 2,03±0,28 while the average percentage of nucleotides of the identified repetitive elements with the respect to the nucleotides of the Aeafru introns (indicated as REbp) is 34,56%±19,54 (Figure S1). To compare these values with the average values of the Ae. aegypti introns, we analysed 1000 randomly chosen Ae. aegypti introns (size range from 1 to 130 kb) with the CENSOR software, defining an average NoRE/kb (2,19±0,68) and REbp (47,22±17,75%) intron values for this species. This analysis indicates that within the introns of Ae. aegypti the number of repetitive elements per kb is almost constant, with a value of about 2, while the size of these elements is variable, ranging from 30% to 60% of the whole intron sequences. The NoRE/kb and REbp values of the Aeafru introns do not deviate significantly this observation. (PDF) [file pone.0048554.s009.pdf]

Table S2 – *Ae. aegypti* intron analysis

Average NoRE/kb	2,19	dS	0,68	Average RE bp	47,22	dS	17,75

Gene ID	Intron number	Intron lenght (bp)	Number of Repeated Element	Total Repeated Element lenght (bp)	NoRE/kb	RE bp (%)
AAEL000001	2-3	5548	16	2462	2,88	44,38
AAEL000020	1-2	2738	9	622	3,29	22,72
AAEL000020	2-3	1054	3	451	2,85	42,79
AAEL000034	2-3	1627	4	560	2,46	34,42
AAEL000060	3-4	14033	24	8486	1,71	60,47
AAEL000060	5-6	4007	9	1481	2,25	36,96
AAEL000070	4-5	3106	6	938	1,93	30,20
AAEL000095	1-2	6986	4	477	0,57	6,83
AAEL000121	2-3	13070	15	9367	1,15	71,67
AAEL000121	5-6	10950	18	6929	1,64	63,28
AAEL000148	2-3	2479	8	873	3,23	35,22
AAEL000159	2-3	15316	31	4995	2,02	32,61
AAEL000160	1-2	15229	28	7527	1,84	49,43
AAEL000174	2-3	14525	37	7568	2,55	52,10
AAEL000195	3-4	20088	42	7827	2,09	38,96
AAEL000201	1-2	1487	5	528	3,36	35,51
AAEL000201	3-4	4097	6	3142	1,46	76,69
AAEL000215	2-3	2953	9	997	3,05	33,76
AAEL000221	2-3	8236	23	3664	2,79	44,49
AAEL000247	1-2	12692	27	5525	2,13	43,53
AAEL000254	2-3	29429	53	17630	1,80	59,91
AAEL000261	1-2	7397	8	3770	1,08	50,97
AAEL000283	1-2	4573	13	2793	2,84	61,08
AAEL000300	1-2	1288	4	332	3,11	25,78
AAEL000310	1-2	10424	21	5949	2,01	57,07
AAEL000325	3-4	14023	30	6231	2,14	44,43
AAEL000349	1-2	13312	24	6029	1,80	45,29
AAEL000376	1-2	3244	6	1067	1,85	32,89
AAEL000397	1-2	6513	14	3540	2,15	54,35
AAEL000402	5-6	7300	20	2504	2,74	34,30
AAEL000405	1-2	1617	2	216	1,24	13,36
AAEL000405	4-5	10507	24	3895	2,28	37,07
AAEL000426	3-4	15717	32	10833	2,04	68,93
AAEL000444	1-2	20117	37	13421	1,84	66,71
AAEL000469	2-3	36765	67	19179	1,82	52,17
AAEL000487	1-2	1156	2	532	1,73	46,02
AAEL000498	1-2	9797	10	5373	1,02	54,84
AAEL000509	2-3	5213	13	1550	2,49	29,73
AAEL000509	4-5	6848	15	1335	2,19	19,49
AAEL000536	2-3	38643	90	20661	2,33	53,47
AAEL000536	5-6	23557	64	8978	2,72	38,11
AAEL000540	1-2	5250	9	2427	1,71	46,23
AAEL000555	1-2	14153	45	7395	3,18	52,25
AAEL000567	1-2	3922	7	3126	1,78	79,70
AAEL000567	2-3	5718	16	2673	2,80	46,75
AAEL000578	1-2	19151	45	8118	2,35	42,39
AAEL000580	1-2	23060	51	10616	2,21	46,04

AAEL000593	2-3	1227	2	762	1,63	62,10
AAEL000600	1-2	37808	92	19978	2,43	52,84
AAEL000617	1-2	3723	7	2149	1,88	57,72
AAEL000643	1-2	13901	37	5289	2,66	38,05
AAEL000666	2-3	10579	21	7361	1,99	69,58
AAEL000683	1-2	5963	10	1289	1,68	21,62
AAEL000700	1-2	10397	12	6325	1,15	60,83
AAEL000714	1-2	8353	17	2801	2,04	33,53
AAEL000714	4-5	16641	37	9396	2,22	56,46
AAEL000728	1-2	106842	187	35786	1,75	33,49
AAEL000728	2-3	9097	18	2940	1,98	32,32
AAEL000738	2-3	10509	20	3188	1,90	30,34
AAEL000750	2-3	11306	23	7297	2,03	64,54
AAEL000761	1-2	3082	6	678	1,95	22,00
AAEL000780	3-4	13648	30	4058	2,20	29,73
AAEL000792	1-2	56434	108	17862	1,91	31,65
AAEL000805	1-2	15941	28	4645	1,76	29,14
AAEL000825	2-3	3175	11	2164	3,46	68,16
AAEL000845	5-6	1123	1	767	0,89	68,30
AAEL000857	1-2	1552	3	677	1,93	43,62
AAEL000857	2-3	4447	16	1733	3,60	38,97
AAEL000869	3-4	6951	8	1064	1,15	15,31
AAEL000883	1-2	7046	15	3255	2,13	46,20
AAEL000898	2-3	10546	22	3892	2,09	36,90
AAEL000906	1-2	11218	12	1546	1,07	13,78
AAEL000927	1-2	15145	38	5113	2,51	33,76
AAEL000943	1-2	31310	60	16698	1,92	53,33
AAEL000965	1-2	1210	4	329	3,31	27,19
AAEL000988	1-2	7612	20	3045	2,63	40,00
AAEL001000	2-3	8161	17	4484	2,08	54,94
AAEL001007	2-3	23267	50	10538	2,15	45,29
AAEL001027	1-2	2821	3	229	1,06	8,12
AAEL001056	1-2	6490	4	5043	0,62	77,70
AAEL001056	4-5	6376	13	2220	2,04	34,82
AAEL001072	1-2	2318	2	1298	0,86	56,00
AAEL001108	2-3	13695	31	4444	2,26	32,45
AAEL001121	6-7	16483	32	5104	1,94	30,97
AAEL001147	3-4	4624	8	4040	1,73	87,37
AAEL001149	2-3	19127	36	9783	1,88	51,15
AAEL001169	3-4	8389	30	3436	3,58	40,96
AAEL001193	1-2	7489	10	4648	1,34	62,06
AAEL001217	1-2	4435	6	2831	1,35	63,83
AAEL001234	1-2	3512	10	1930	2,85	54,95
AAEL001254	3-4	3316	11	1667	3,32	50,27
AAEL001261	1-2	7090	9	5392	1,27	76,05
AAEL001262	1-2	15854	36	7527	2,27	47,48
AAEL001278	1-2	8088	23	6857	2,84	84,78
AAEL001279	2-3	6402	13	2096	2,03	32,74
AAEL001297	5-6	15308	34	5575	2,22	36,42
AAEL001307	1-2	13670	28	5572	2,05	40,76
AAEL001323	1-2	10840	31	6877	2,86	63,44
AAEL001349	5-6	5865	20	4486	3,41	76,49
AAEL001371	4-5	1283	1	38	0,78	2,96
AAEL001393	1-2	12867	22	8260	1,71	64,20
AAEL001400	1-2	1803	3	321	1,66	17,80
AAEL001423	1-2	15857	35	6599	2,21	41,62
AAEL001458	2-3	65909	117	31789	1,78	48,23

AAEL001478	1-2	5279	3	2455	0,57	46,51
AAEL001494	3-4	16164	51	9845	3,16	60,91
AAEL001500	3-4	22596	48	9404	2,12	41,62
AAEL001500	4-5	6208	11	2201	1,77	35,45
AAEL001527	2-3	13837	21	5509	1,52	39,81
AAEL001527	3-4	9769	17	2618	1,74	26,80
AAEL001538	1-2	31688	53	22052	1,67	69,59
AAEL001549	1-2	15770	37	8782	2,35	55,69
AAEL001578	1-2	10906	23	3231	2,11	29,63
AAEL001593	1-2	14111	38	5477	2,69	38,81
AAEL001601	2-3	3977	2	3435	0,50	86,37
AAEL001622	2-3	14815	23	11908	1,55	80,38
AAEL001632	1-2	6074	14	3936	2,30	64,80
AAEL001632	3-4	5927	12	2206	2,02	37,22
AAEL001658	4-5	26006	39	17991	1,50	69,18
AAEL001660	1-2	7441	13	4731	1,75	63,58
AAEL001687	2-3	11874	21	5360	1,77	45,14
AAEL001709	3-4	12805	31	5881	2,42	45,93
AAEL001715	2-3	1083	3	258	2,77	23,82
AAEL001731	1-2	5992	4	3875	0,67	64,67
AAEL001750	1-2	2004	5	646	2,50	32,24
AAEL001765	5-6	1148	2	193	1,74	16,81
AAEL001784	1-2	8077	18	3764	2,23	46,60
AAEL001798	1-2	6600	25	4975	3,79	75,38
AAEL001798	2-3	9171	19	4419	2,07	48,18
AAEL001809	1-2	8551	26	2977	3,04	34,81
AAEL001817	1-2	16172	37	7577	2,29	46,85
AAEL001829	2-3	1856	4	549	2,16	29,58
AAEL001844	2-3	10651	13	7033	1,22	66,03
AAEL001851	1-2	11664	26	3673	2,23	31,49
AAEL001867	1-2	15688	36	11733	2,29	74,79
AAEL001875	2-3	116412	192	49640	1,65	42,64
AAEL001896	1-2	39209	78	11155	1,99	28,45
AAEL001901	1-2	5436	11	3368	2,02	61,96
AAEL001901	2-3	8527	18	2166	2,11	25,40
AAEL001919	4-5	1635	3	358	1,83	21,90
AAEL001933	1-2	67624	144	29316	2,13	43,35
AAEL001945	1-2	2699	6	922	2,22	34,16
AAEL001976	1-2	4030	9	1796	2,23	44,57
AAEL001976	2-3	1177	5	480	4,25	40,78
AAEL001979	2-3	8758	19	4654	2,17	53,14
AAEL001982	1-2	2556	6	721	2,35	28,21
AAEL001990	1-2	1072	1	1072	0,93	100,00
AAEL002009	3-4	13728	33	5243	2,40	38,19
AAEL002018	3-4	17181	24	7288	1,40	42,42
AAEL002031	1-2	7631	22	3819	2,88	50,05
AAEL002049	1-2	4929	11	2336	2,23	47,39
AAEL002049	4-5	13927	30	6996	2,15	50,23
AAEL002067	1-2	2248	3	1864	1,33	82,92
AAEL002075	1-2	5396	10	1596	1,85	29,58
AAEL002097	2-3	14783	32	5381	2,16	36,40
AAEL002100	1-2	5645	14	2437	2,48	43,17
AAEL002100	2-3	40619	67	16205	1,65	39,90
AAEL002115	2-3	24824	55	14069	2,22	56,67
AAEL002132	1-2	10741	30	5622	2,79	52,34
AAEL002132	2-3	7618	24	3150	3,15	41,35
AAEL002145	4-5	11198	17	6115	1,52	54,61

AAEL002154	2-3	3283	3	2782	0,91	84,74
AAEL002170	1-2	29943	56	16871	1,87	56,34
AAEL002194	1-2	17645	49	7300	2,78	41,37
AAEL002205	1-2	3049	10	911	3,28	29,88
AAEL002220	3-4	27738	70	18305	2,52	65,99
AAEL002235	1-2	10998	20	6612	1,82	60,12
AAEL002250	1-2	1598	3	1341	1,88	83,92
AAEL002277	1-2	6570	18	2871	2,74	43,70
AAEL002299	1-2	17242	25	6168	1,45	35,77
AAEL002300	1-2	3333	1	3333	0,30	100,00
AAEL002319	2-3	19598	34	12504	1,73	63,80
AAEL002330	1-2	9968	17	7324	1,71	73,48
AAEL002345	2-3	7992	27	4280	3,38	53,55
AAEL002345	3-4	19159	40	11964	2,09	62,45
AAEL002376	2-3	6471	16	2093	2,47	32,34
AAEL002397	1-2	27371	65	11019	2,37	40,26
AAEL002403	1-2	5029	1	4482	0,20	89,12
AAEL002421	1-2	4685	11	2231	2,35	47,62
AAEL002446	1-2	11613	28	3255	2,41	28,03
AAEL002446	5-6	1770	2	253	1,13	14,29
AAEL002465	2-3	10253	13	5265	1,27	51,35
AAEL002487	4-5	8727	11	1619	1,26	18,55
AAEL002500	1-2	4791	6	1695	1,25	35,38
AAEL002521	1-2	6035	7	5124	1,16	84,90
AAEL002544	2-3	4366	8	3709	1,83	84,95
AAEL002557	2-3	3137	8	1343	2,55	42,81
AAEL002575	1-2	7565	18	2904	2,38	38,39
AAEL002575	4-5	8017	22	4215	2,74	52,58
AAEL002581	1-2	13263	30	3629	2,26	27,36
AAEL002593	2-3	10636	18	7175	1,69	67,46
AAEL002600	1-2	5922	19	3760	3,21	63,49
AAEL002615	1-2	4497	9	1229	2,00	27,33
AAEL002627	1-2	3913	14	2503	3,58	63,97
AAEL002649	1-2	3221	7	1347	2,17	41,82
AAEL002661	1-2	16905	33	11628	1,95	68,78
AAEL002687	1-2	10862	12	6856	1,10	63,12
AAEL002700	2-3	8869	18	6988	2,03	78,79
AAEL002710	1-2	2680	4	2498	1,49	93,21
AAEL002715	1-2	9586	19	5408	1,98	56,42
AAEL002727	3-4	18724	42	10132	2,24	54,11
AAEL002758	1-2	16143	27	7074	1,67	43,82
AAEL002770	1-2	25117	35	20564	1,39	81,87
AAEL002789	1-2	15673	29	6002	1,85	38,30
AAEL002802	1-2	16959	13	12589	0,77	74,23
AAEL002819	2-3	20199	37	13141	1,83	65,06
AAEL002835	2-3	1818	3	161	1,65	8,86
AAEL002851	1-2	15591	20	3135	1,28	20,11
AAEL002869	1-2	3733	6	1368	1,61	36,65
AAEL002888	1-2	1370	2	140	1,46	10,22
AAEL002903	1-2	26041	61	8976	2,34	34,47
AAEL002913	1-2	20038	49	8131	2,45	40,58
AAEL002922	1-2	9925	22	5644	2,22	56,87
AAEL002925	2-3	11513	22	8990	1,91	78,09
AAEL002937	1-2	20341	48	10997	2,36	54,06
AAEL002949	1-2	29851	73	10355	2,45	34,69
AAEL002961	1-2	11730	17	6588	1,45	56,16
AAEL002971	1-2	17558	39	6289	2,22	35,82

AAEL002985	3-4	13636	29	6446	2,13	47,27
AAEL002987	1-2	16226	28	7858	1,73	48,43
AAEL003003	1-2	26321	63	8625	2,39	32,77
AAEL003015	1-2	39477	75	18092	1,90	45,83
AAEL003015	3-4	13229	23	6230	1,74	47,09
AAEL003027	2-3	14595	29	5801	1,99	39,75
AAEL003052	1-2	44650	112	19155	2,51	42,90
AAEL003078	2-3	25056	79	12833	3,15	51,22
AAEL003091	4-5	10797	34	7027	3,15	65,08
AAEL003107	2-3	5013	30	4232	5,98	84,42
AAEL003126	1-2	7431	25	3860	3,36	51,94
AAEL003129	1-2	24999	58	13938	2,32	55,75
AAEL003154	3-4	14267	34	7995	2,38	56,04
AAEL003179	1-2	11137	17	5111	1,53	45,89
AAEL003199	2-3	6594	12	1391	1,82	21,09
AAEL003205	1-2	3674	11	3119	2,99	84,89
AAEL003220	1-2	81976	152	39643	1,85	48,36
AAEL003220	3-4	18454	34	8531	1,84	46,23
AAEL003229	2-3	3451	9	1915	2,61	55,49
AAEL003243	1-2	7090	9	5392	1,27	76,05
AAEL003269	1-2	9196	18	2323	1,96	25,26
AAEL003291	1-2	21506	41	11746	1,91	54,62
AAEL003301	1-2	23890	50	13537	2,09	56,66
AAEL003318	1-2	13214	21	2971	1,59	22,48
AAEL003327	1-2	5696	16	1888	2,81	33,15
AAEL003349	3-4	19214	28	8129	1,46	42,31
AAEL003363	2-3	1439	2	916	1,39	63,66
AAEL003381	1-2	18901	44	8574	2,33	45,36
AAEL003399	1-2	17238	34	10571	1,97	61,32
AAEL004005	1-2	3948	11	1982	2,79	50,20
AAEL004017	2-3	9661	32	4495	3,31	46,53
AAEL004031	1-2	6434	19	2571	2,95	39,96
AAEL004056	1-2	1257	3	142	2,39	11,30
AAEL004070	1-2	4991	7	1714	1,40	34,34
AAEL004076	1-2	2448	5	448	2,04	18,30
AAEL004099	2-3	4816	11	3186	2,28	66,15
AAEL004105	1-2	4804	10	2281	2,08	47,48
AAEL004117	2-3	7657	17	2637	2,22	34,44
AAEL004132	2-3	1208	2	215	1,66	17,80
AAEL004152	1-2	6802	18	2001	2,65	29,42
AAEL004177	2-3	30367	58	18523	1,91	61,00
AAEL004189	1-2	7062	32	5374	4,53	76,10
AAEL004199	1-2	1270	4	900	3,15	70,87
AAEL004199	2-3	1747	4	243	2,29	13,91
AAEL004200	2-3	13916	39	6550	2,80	47,07
AAEL004208	5-6	2803	6	737	2,14	26,29
AAEL004212	1-2	48577	109	18459	2,24	38,00
AAEL004221	1-2	7648	23	2902	3,01	37,94
AAEL004235	1-2	5147	15	1812	2,91	35,20
AAEL004251	1-2	12020	33	5887	2,75	48,98
AAEL004268	4-5	22316	42	15120	1,88	67,75
AAEL004281	2-3	9799	22	7259	2,25	74,08
AAEL004297	1-2	10894	26	6532	2,39	59,96
AAEL004301	2-3	6373	19	3768	2,98	59,12
AAEL004311	1-2	13104	38	7523	2,90	57,41
AAEL004317	2-3	22518	45	15727	2,00	69,84
AAEL004327	1-2	1705	6	841	3,52	49,33

AAEL004327	2-3	4903	5	3946	1,02	80,48
AAEL004346	1-2	10753	14	1708	1,30	15,88
AAEL004351	1-2	11902	24	3376	2,02	28,36
AAEL004375	2-3	17892	43	7525	2,40	42,06
AAEL004381	1-2	7188	7	3754	0,97	52,23
AAEL004381	3-4	32268	28	7305	0,87	22,64
AAEL004393	4-5	15683	22	3045	1,40	19,42
AAEL004401	1-2	2682	6	728	2,24	27,14
AAEL004410	3-4	12985	29	6339	2,23	48,82
AAEL004418	1-2	3471	8	2416	2,30	69,61
AAEL004426	1-2	11124	30	7688	2,70	69,11
AAEL004432	2-3	9672	17	3690	1,76	38,15
AAEL004457	2-3	4333	10	1083	2,31	24,99
AAEL004465	2-3	28676	54	21267	1,88	74,16
AAEL004471	1-2	7903	24	4476	3,04	56,64
AAEL004494	1-2	6940	17	3123	2,45	45,00
AAEL004501	2-3	13521	33	5029	2,44	37,19
AAEL004511	1-2	1838	4	844	2,18	45,92
AAEL004515	3-4	14552	33	10264	2,27	70,53
AAEL004518	1-2	2048	1	73	0,49	3,56
AAEL004522	1-2	1346	5	620	3,71	46,06
AAEL004534	3-4	52971	115	19307	2,17	36,45
AAEL004559	1-2	19199	38	6649	1,98	34,63
AAEL004571	1-2	8398	18	2726	2,14	32,46
AAEL004587	1-2	8375	17	5325	2,03	63,58
AAEL004601	1-2	8225	20	3131	2,43	38,07
AAEL004624	2-3	10765	24	4257	2,23	39,54
AAEL004653	2-3	14814	25	8299	1,69	56,02
AAEL004673	1-2	6092	25	3297	4,10	54,12
AAEL004691	4-5	12924	24	6363	1,86	49,23
AAEL004703	1-2	17245	47	8366	2,73	48,51
AAEL004719	2-3	2205	3	1163	1,36	52,74
AAEL004719	3-4	23383	48	10350	2,05	44,26
AAEL004725	1-2	14473	30	3987	2,07	27,55
AAEL004732	1-2	28344	67	13102	2,36	46,22
AAEL004739	2-3	14968	26	8901	1,74	59,47
AAEL004739	2-3	14968	26	8901	1,74	59,47
AAEL004741	1-2	8831	16	3006	1,81	34,04
AAEL004756	3-4	5462	8	4962	1,46	90,85
AAEL004761	2-3	5385	9	1043	1,67	19,37
AAEL004774	2-3	11957	31	5393	2,59	45,10
AAEL004784	1-2	1068	3	383	2,81	35,86
AAEL004796	1-2	7754	20	1483	2,58	19,13
AAEL004800	1-2	5962	12	2069	2,01	34,70
AAEL004805	2-3	12029	39	6453	3,24	53,65
AAEL004815	2-3	8313	14	7289	1,68	87,68
AAEL004838	1-2	5738	16	2618	2,79	45,63
AAEL004855	1-2	5395	9	1392	1,67	25,80
AAEL004866	2-3	5741	15	2925	2,61	50,95
AAEL004872	1-2	22156	55	10380	2,48	46,85
AAEL004890	1-2	12405	15	8302	1,21	66,92
AAEL004900	3-4	11314	23	3573	2,03	31,58
AAEL004938	1-2	43738	80	20524	1,83	46,92
AAEL004951	2-3	10894	23	4301	2,11	39,48
AAEL004977	2-3	10164	14	4978	1,38	48,98
AAEL004985	1-2	3485	11	1160	3,16	33,29
AAEL004991	1-2	7772	19	3401	2,44	43,76

AAEL005001	1-2	27369	62	15402	2,27	56,28
AAEL005030	3-4	15091	30	9130	1,99	60,50
AAEL005080	1-2	16721	39	6584	2,33	39,38
AAEL005080	5-6	4590	15	4074	3,27	88,76
AAEL005121	1-2	26307	43	14474	1,63	55,02
AAEL005121	3-4	9194	22	4450	2,39	48,40
AAEL005200	1-2	16729	25	8306	1,49	49,65
AAEL005200	3-4	16681	45	7042	2,70	42,22
AAEL005290	1-2	9943	27	5356	2,72	53,87
AAEL005290	2-3	1044	5	753	4,79	72,13
AAEL005330	2-3	10229	23	6200	2,25	60,61
AAEL005351	4-5	1836	5	976	2,72	53,16
AAEL005372	2-3	22846	47	10152	2,06	44,44
AAEL005380	3-4	15675	28	5625	1,79	35,89
AAEL005400	3-4	9141	28	6115	3,06	66,90
AAEL005422	1-2	27270	59	14809	2,16	54,31
AAEL005422	6-7	5706	15	2150	2,63	37,68
AAEL005440	1-2	3502	9	1392	2,57	39,75
AAEL005440	2-3	5355	9	3098	1,68	57,85
AAEL005460	1-2	42677	88	22671	2,06	53,12
AAEL005460	4-5	9238	32	4454	3,46	48,21
AAEL005490	4-5	12621	38	7080	3,01	56,10
AAEL005495	3-4	8339	17	3495	2,04	41,91
AAEL005495	8-9	2935	4	2047	1,36	69,74
AAEL005500	1-2	33833	59	24871	1,74	73,51
AAEL005510	2-3	1654	2	612	1,21	37,00
AAEL005510	3-4	45444	77	28879	1,69	63,55
AAEL005515	4-5	2474	7	973	2,83	39,33
AAEL005515	5-6	4917	4	306	0,81	6,22
AAEL005520	2-3	5550	19	1735	3,42	31,26
AAEL005530	1-2	33289	74	12321	2,22	37,01
AAEL005530	2-3	16802	26	8369	1,55	49,81
AAEL005560	4-5	6534	16	2190	2,45	33,52
AAEL005570	7-8	2732	9	1557	3,29	56,99
AAEL005580	1-2	6392	10	1689	1,56	26,42
AAEL005615	1-2	11890	24	9531	2,02	80,16
AAEL005627	1-2	1165	4	295	3,43	25,32
AAEL005640	2-3	44149	53	24441	1,20	55,36
AAEL005655	1-2	16988	36	4162	2,12	24,50
AAEL005672	1-2	12876	37	7449	2,87	57,85
AAEL005680	2-3	10487	22	6092	2,10	58,09
AAEL005683	1-2	9233	32	6330	3,47	68,56
AAEL005697	4-5	36730	70	12350	1,91	33,62
AAEL005700	1-2	28023	52	14641	1,86	52,25
AAEL005740	2-3	7547	18	2746	2,39	36,39
AAEL005750	6-7	24474	53	15342	2,17	62,69
AAEL005762	1-2	17756	39	7993	2,20	45,02
AAEL005773	1-2	1364	6	1210	4,40	88,71
AAEL005780	3-4	1615	3	1119	1,86	69,29
AAEL005790	1-2	10696	20	4251	1,87	39,74
AAEL005795	2-3	11221	25	5879	2,23	52,39
AAEL009002	2-3	14159	39	6055	2,75	42,76
AAEL008001	1-2	29939	51	14828	1,70	49,53
AAEL008001	2-3	16420	37	6140	2,25	37,39
AAEL005801	4-5	10940	26	4393	2,38	40,16
AAEL005099	2-3	7336	23	2896	3,14	39,48
AAEL005821	2-3	9199	24	3492	2,61	37,96

AAEL005842	2-3	5448	11	3586	2,02	65,82
AAEL005858	1-2	11855	25	6888	2,11	58,10
AAEL005874	3-4	20798	50	10066	2,40	48,40
AAEL005892	1-2	36997	74	14421	2,00	38,98
AAEL005911	2-3	3723	13	1343	3,49	36,07
AAEL005927	1-2	1465	4	519	2,73	35,43
AAEL005942	8-9	7496	18	4187	2,40	55,86
AAEL005960	1-2	11872	12	5441	1,01	45,83
AAEL005975	1-2	15419	43	7152	2,79	46,38
AAEL005991	2-3	8809	19	3592	2,16	40,78
AAEL006012	6-7	15780	44	8777	2,79	55,62
AALE006031	2-3	7503	19	3956	2,53	52,73
AAEL006047	1-2	3723	8	1235	2,15	33,17
AAEL006062	1-2	4048	4	504	0,99	12,45
AAEL006085	1-2	51361	80	28268	1,56	55,04
AAEL006102	1-2	8984	14	1705	1,56	18,98
AAEL006120	2-3	21347	45	9144	2,11	42,84
AAEL006137	1-2	4371	11	2575	2,52	58,91
AAEL006140	2-3	20806	48	13077	2,31	62,85
AAEL006160	1-2	79601	160	51027	2,01	64,10
AAEL006080	3-4	22373	48	7730	2,15	34,55
AAEL006204	1-2	29412	63	16584	2,14	56,39
AAEL006222	1-2	3378	10	1136	2,96	33,63
AAEL006240	3-4	14174	37	4419	2,61	31,18
AAEL006257	1-2	13518	23	8718	1,70	64,49
AAEL006271	3-4	14419	24	7422	1,66	51,47
AAEL006291	1-2	5650	20	3335	3,54	59,03
AAEL006300	2-3	16771	34	9772	2,03	58,27
AAEL006318	1-2	5465	11	1277	2,01	23,37
AAEL006339	1-2	10739	30	3485	2,79	32,45
AAEL006369	2-3	28943	60	10804	2,07	37,33
AAEL006380	2-3	16474	37	7041	2,25	42,74
AAEL006397	3-4	8806	19	2773	2,16	31,49
AAEL006409	3-4	2580	8	1802	3,10	69,84
AAEL006422	2-3	20528	49	12532	2,39	61,05
AAEL006440	2-3	3783	10	1460	2,64	38,59
AAEL006459	1-2	4329	12	963	2,77	22,25
AAEL006478	5-6	30795	46	18469	1,49	59,97
AAEL006492	2-3	8946	22	4659	2,46	52,08
AAEL006510	1-2	18612	19	12086	1,02	64,94
AAEL006527	1-2	5431	11	2507	2,03	46,16
AAEL006545	1-2	4218	11	1562	2,61	37,03
AAEL006561	1-2	4278	10	2288	2,34	53,48
AAEL006581	1-2	29808	64	13920	2,15	46,70
AAEL006599	6-7	10174	18	5864	1,77	57,64
AAEL006621	1-2	13582	28	8189	2,06	60,29
AAEL006650	1-2	13171	32	6190	2,43	47,00
AAEL006669	1-2	2814	7	2201	2,49	78,22
AAEL006683	1-2	23903	53	10131	2,22	42,38
AAEL006698	2-3	5873	8	1245	1,36	21,20
AAEL006719	1-2	1765	2	1436	1,13	81,36
AAEL006733	3-4	16425	25	7696	1,52	46,86
AAEL006750	1-2	4781	10	2363	2,09	49,42
AAEL006768	1-2	10796	23	4952	2,13	45,87
AAEL006790	1-2	11103	30	5488	2,70	49,43
AAEL006812	1-2	22541	59	8489	2,62	37,66
AAEL006830	1-2	24214	50	7607	2,06	31,42

AAEL006847	1-2	14176	30	9655	2,12	68,11
AAEL006861	2-3	3647	3	2928	0,82	80,29
AAEL006885	5-6	8515	16	2561	1,88	30,08
AAEL006899	2-3	10362	29	5835	2,80	56,31
AAEL006899	3-4	32417	74	11590	2,28	35,75
AAEL006912	2-3	4875	11	1865	2,26	38,26
AAEL006934	1-2	8253	19	2375	2,30	28,78
AAEL006951	2-3	10747	19	7267	1,77	67,62
AAEL006970	1-2	5597	13	1385	2,32	24,75
AAEL006881	4-5	8687	23	4508	2,65	51,89
AAEL006881	8-9	3873	12	2152	3,10	55,56
AAEL006899	3-4	32417	74	11590	2,28	35,75
AAEL006910	2-3	14707	35	8674	2,38	58,98
AAEL006922	2-3	89357	193	38772	2,16	43,39
AAEL006942	3-4	16421	39	6224	2,38	37,90
AAEL006955	3-4	19508	56	11832	2,87	60,65
AAEL006970	2-3	12678	30	4591	2,37	36,21
AAEL006982	1-2	4055	5	1945	1,23	47,97
AAEL006993	2-3	26619	44	11102	1,65	41,71
AAEL007001	1-2	9902	24	5119	2,42	51,70
AAEL007015	1-2	7058	23	4711	3,26	66,75
AAEL007028	1-2	14576	32	6912	2,20	47,42
AAEL007040	2-3	45208	93	14616	2,06	32,33
AAEL007065	3-4	24781	54	13942	2,18	56,26
AAEL007082	9-10	10440	27	4030	2,59	38,60
AAEL007100	4-5	16625	30	14558	1,80	87,57
AAEL007114	1-2	15004	25	4506	1,67	30,03
AAEL007129	2-3	12855	19	6643	1,48	51,68
AAEL007139	1-2	14674	27	5571	1,84	37,97
AAEL007151	3-4	34914	84	13262	2,41	37,98
AAEL007164	4-5	24190	49	13847	2,03	57,24
AAEL007178	4-5	4690	11	1289	2,35	27,48
AAEL007191	1-2	5459	11	1581	2,02	28,96
AAEL007200	2-3	28702	60	13688	2,09	47,69
AAEL007218	1-2	31664	68	12615	2,15	39,84
AAEL007128	6-7	14505	41	8822	2,83	60,82
AAEL007230	1-2	4901	11	1385	2,24	28,26
AAEL007244	3-4	1905	6	1740	3,15	91,34
AAEL007255	2-3	19270	33	6139	1,71	31,86
AAEL007268	1-2	5002	14	2779	2,80	55,56
AAEL007280	1-2	6314	10	2358	1,58	37,35
AAEL007293	1-2	10676	32	5109	3,00	47,86
AAEL007310	1-2	10408	33	5037	3,17	48,40
AAEL007322	4-5	28794	51	16280	1,77	56,54
AAEL007331	3-4	11951	29	6862	2,43	57,42
AAEL007342	1-2	22861	47	14794	2,06	64,71
AAEL007342	2-3	12121	25	7227	2,06	59,62
AAEL007359	4-5	47883	91	29178	1,90	60,94
AAEL007368	1-2	11020	22	3866	2,00	35,08
AAEL007381	2-3	20341	35	11386	1,72	55,98
AAEL007397	2-3	55632	100	11208	1,80	20,15
AAEL007397	3-4	18169	23	3362	1,27	18,50
AAEL007410	3-4	9491	20	5872	2,11	61,87
AAEL007425	1-2	44540	94	18811	2,11	42,23
AAEL007435	1-2	10505	27	3816	2,57	36,33
AAEL007438	1-2	5444	16	2117	2,94	38,89
AAEL007448	2-3	11148	21	6322	1,88	56,71

AAEL007459	1-2	5418	2	5210	0,37	96,16
AAEL007470	1-2	37584	79	16731	2,10	44,52
AAEL007483	1-2	25851	64	14477	2,48	56,00
AAEL007498	4-5	8414	29	4058	3,45	48,23
AAEL007512	1-2	11696	26	3562	2,22	30,45
AAEL007524	1-2	1235	1	39	0,81	3,16
AAEL007536	1-2	10355	30	5272	2,90	50,91
AAEL007563	1-2	174301	351	78299	2,01	44,92
AAEL007575	1-2	8651	23	5174	2,66	59,81
AAEL007587	1-2	3147	12	946	3,81	30,06
AAEL007597	1-2	23022	49	7730	2,13	33,58
AAEL007610	1-2	14041	14	5683	1,00	40,47
AAEL007610	3-4	34325	69	20221	2,01	58,91
AAEL007624	1-2	53750	117	23886	2,18	44,44
AAEL007638	1-2	7755	18	2237	2,32	28,85
AAEL007649	2-3	7602	18	4418	2,37	58,12
AAEL007662	1-2	16638	44	6199	2,64	37,26
AAEL007678	1-2	7063	21	5278	2,97	74,73
AAEL007680	1-2	10533	36	4871	3,42	46,25
AAEL007693	1-2	9739	30	4313	3,08	44,29
AAEL007693	2-3	9366	20	5906	2,14	63,06
AAEL007721	1-2	7141	24	4451	3,36	62,33
AAEL007731	4-5	10320	24	4384	2,33	42,48
AAEL007739	1-2	7874	15	2387	1,91	30,31
AAEL007748	1-2	5143	18	3386	3,50	65,84
AAEL007748	2-3	6200	15	1550	2,42	25,00
AAEL007763	2-3	10615	21	7373	1,98	69,46
AAEL007770	2-3	33924	69	20021	2,03	59,02
AAEL007770	4-5	10775	23	3962	2,13	36,77
AAEL007791	1-2	5977	10	3400	1,67	56,88
AAEL007799	3-4	3914	5	598	1,28	15,28
AAEL007810	2-3	12861	15	9361	1,17	72,79
AAEL007820	2-3	7164	22	2938	3,07	41,01
AAEL007830	1-2	4626	11	1343	2,38	29,03
AAEL007843	2-3	4887	7	1920	1,43	39,29
AAEL007856	5-6	5205	3	4793	0,58	92,08
AAEL007880	1-2	1409	1	54	0,71	3,83
AAEL007894	1-2	1422	4	291	2,81	20,46
AAEL007902	1-2	4173	11	2726	2,64	65,32
AAEL007912	1-2	5120	18	2196	3,52	42,89
AAEL007920	1-2	21203	31	11108	1,46	52,39
AAEL007930	2-3	15576	29	8453	1,86	54,27
AAEL007944	1-2	1692	1	1079	0,59	63,77
AAEL007050	1-2	9196	19	4489	2,07	48,81
AAEL007965	1-2	7184	23	5264	3,20	73,27
AAEL007978	2-3	13877	27	3363	1,95	24,23
AAEL007990	1-2	41940	78	22209	1,86	52,95
AALE008001	2-3	16420	37	6140	2,25	37,39
AAEL008010	5-6	131077	235	80846	1,79	61,68
AALE008015	1-2	32485	68	10298	2,09	31,70
AAEL008021	1-2	8336	18	5521	2,16	66,23
AAEL008028	1-2	37348	65	15517	1,74	41,55
AAEL008034	1-2	9772	19	1504	1,94	15,39
AAEL008042	1-2	1770	6	1247	3,39	70,45
AAEL008056	1-2	25033	53	7885	2,12	31,50
AAEL008060	2-3	16118	46	7987	2,85	49,55
AAEL008063	3-4	1845	1	57	0,54	3,09

AAEL008078	2-3	5940	23	4274	3,87	71,95
AAEL008078	8-9	12371	18	9193	1,46	74,31
AAEL008096	1-2	12188	13	6420	1,07	52,67
AAEL008111	1-2	8022	16	3665	1,99	45,69
AAEL008123	1-2	10466	25	5038	2,39	48,14
AAEL008135	4-5	3529	11	3187	3,12	90,31
AAEL008146	3-4	18929	25	11701	1,32	61,82
AAEL008170	2-3	3950	9	2424	2,28	61,37
AAEL008183	1-2	10767	24	4441	2,23	41,25
AAEL008200	1-2	5240	10	3466	1,91	66,15
AAEL008216	1-2	5538	12	1645	2,17	29,70
AAEL008230	1-2	5670	10	2096	1,76	36,97
AAEL008242	1-2	14715	31	4515	2,11	30,68
AAEL008260	1-2	5282	15	1877	2,84	35,54
AAEL008272	1-2	1506	9	652	5,98	43,29
AAEL008285	1-2	7963	11	1122	1,38	14,09
AAEL008298	3-4	13401	29	4222	2,16	31,51
AAEL008314	1-2	7847	15	2822	1,91	35,96
AAEL008340	2-3	10008	26	5501	2,60	54,97
AAEL008357	1-2	2403	4	490	1,66	20,39
AAEL008390	2-3	5029	13	3677	2,59	73,12
AAEL008420	1-2	20798	60	11968	2,88	57,54
AAEL008459	3-4	13515	32	8190	2,37	60,60
AAEL008479	1-2	5972	14	5219	2,34	87,39
AAEL008893	1-2	21360	56	9034	2,62	42,29
AAEL008498	2-3	19452	56	7617	2,88	39,16
AAEL008520	1-2	16627	30	10542	1,80	63,40
AAEL008545	1-2	9452	30	3684	3,17	38,98
AAEL008578	1-2	17172	44	7561	2,56	44,03
AAEL008610	1-2	7978	25	5202	3,13	65,20
AAEL008634	1-2	9241	23	3600	2,49	38,96
AAEL008650	3-4	9439	23	5644	2,44	59,79
AAEL008670	5-6	1299	2	119	1,54	9,16
AAEL009002	1-2	33677	67	17897	1,99	53,14
AAEL009002	2-3	14159	39	6055	2,75	42,76
AAEL009024	1-2	8493	23	4641	2,71	54,65
AAEL009024	4-5	12358	32	4520	2,59	36,58
AAEL009049	1-2	4045	6	3131	1,48	77,40
AAEL009049	4-5	12724	16	6192	1,26	48,66
AAEL009068	1-2	9189	17	5252	1,85	57,16
AAEL009083	1-2	5491	2	4705	0,36	85,69
AAEL009100	1-2	1634	4	814	2,45	49,82
AAEL009100	4-5	7856	23	2962	2,93	37,70
AAEL009110	1-2	4836	7	640	1,45	13,23
AAEL009110	3-4	13168	36	8531	2,73	64,79
AAEL009139	1-2	7041	15	4896	2,13	69,54
AAEL009154	3-4	9617	23	3815	2,39	39,67
AAEL009154	4-5	1127	4	205	3,55	18,19
AAEL009173	1-2	18752	40	10944	2,13	58,36
AAEL009173	2-3	11779	30	6380	2,55	54,16
AAEL009193	1-2	8979	26	3855	2,90	42,93
AAEL009200	1-2	12226	22	6219	1,80	50,87
AAEL009221	1-2	1683	2	450	1,19	26,74
AAEL009221	2-3	2272	7	922	3,08	40,58
AAEL009246	5-6	7166	17	5129	2,37	71,57
AAEL009268	5-6	11823	16	8117	1,35	68,65
AAEL009281	1-2	6639	19	2618	2,86	39,43

AAEL009297	2-3	11656	34	4655	2,92	39,94
AAEL009300	1-2	6957	19	4369	2,73	62,80
AAEL009324	1-2	35034	58	21119	1,66	60,28
AAEL009347	1-2	4219	12	3242	2,84	76,84
AAEL009369	1-2	1655	2	719	1,21	43,44
AAEL009369	2-3	18533	31	9277	1,67	50,06
AAEL009381	2-3	11037	21	4918	1,90	44,56
AAEL009400	1-2	14835	37	5049	2,49	34,03
AAEL009415	1-2	5035	12	1318	2,38	26,18
AAEL009437	1-2	13060	33	7634	2,53	58,45
AAEL009437	5-6	4309	10	1396	2,32	32,40
AAEL009451	1-2	1087	3	374	2,76	34,41
AAEL009472	1-2	1882	1	417	0,53	22,16
AAEL009495	2-3	7573	20	2388	2,64	31,53
AAEL009495	5-6	10316	18	7309	1,74	70,85
AAEL009500	2-3	6142	12	3496	1,95	56,92
AAEL009521	1-2	44903	123	24995	2,74	55,66
AAEL009521	2-3	3236	11	1860	3,40	57,48
AAEL009543	1-2	17901	38	7956	2,12	44,44
AAEL009561	1-2	24654	45	17066	1,83	69,22
AAEL009585	1-2	31136	70	16367	2,25	52,57
AAEL009600	4-5	11444	22	4555	1,92	39,80
AAEL009616	1-2	47596	88	27452	1,85	57,68
AAEL009634	3-4	21091	49	8376	2,32	39,71
AAEL009634	4-5	28060	53	11747	1,89	41,86
AAEL009654	2-3	23257	40	12524	1,72	53,85
AAEL009674	5-6	2533	6	1026	2,37	40,51
AAEL009695	1-2	8916	13	3012	1,46	33,78
AAEL009695	2-3	4090	8	2690	1,96	65,77
AAEL009700	1-2	2329	7	820	3,01	35,21
AAEL009725	1-2	2655	8	1490	3,01	56,12
AAEL009747	2-3	14380	25	9349	1,74	65,01
AAEL009765	1-2	16909	30	5935	1,77	35,10
AAEL009780	1-2	7262	16	3534	2,20	48,66
AAEL009800	1-2	4428	14	3510	3,16	79,27
AAEL009817	1-2	1963	8	1032	4,08	52,57
AAEL009848	1-2	1297	2	224	1,54	17,27
AAEL009861	1-2	40032	78	16928	1,95	42,29
AAEL009879	2-3	5332	15	3340	2,81	62,64
AAEL009898	2-3	12899	28	10129	2,17	78,53
AAEL009900	1-2	1443	4	559	2,77	38,74
AAEL009925	1-2	35975	85	14562	2,36	40,48
AAEL009942	1-2	1506	3	916	1,99	60,82
AAEL009942	3-4	28294	42	14326	1,48	50,63
AAEL009960	2-3	15554	41	7297	2,64	46,91
AAEL009976	2-3	3216	10	785	3,11	24,41
AAEL009976	3-4	15776	36	9315	2,28	59,05
AAEL009995	1-2	18140	24	13005	1,32	71,69
AAEL009915	2-3	10150	18	5478	1,77	53,97
AAEL009915	5-6	9333	19	2051	2,04	21,98
AAEL010000	3-4	8.617	23	2718	2,67	31,54
AAEL010020	2-3	20.833	58	9546	2,78	45,82
AAEL010043	1-2	8159	13	1850	1,59	22,67
AAEL010064	1-2	22132	44	9087	1,99	41,06
AAEL010085	1-2	4333	13	1130	3,00	26,08
AAEL010090	1-2	3168	6	1052	1,89	33,21
AAEL010105	1-2	4542	13	3880	2,86	85,42

AAEL010127	1-2	5058	11	1653	2,17	32,68
AAEL10133	1-2	4123	8	3303	1,94	80,11
AAEL010148	1-2	38816	60	17829	1,55	45,93
AAEL010162	1-2	10587	26	5523	2,46	52,17
AAEL010184	1-2	30315	53	20267	1,75	66,85
AAEL010200	2-3	16734	30	6855	1,79	40,96
AAEL010200	3-4	4592	12	1177	2,61	25,63
AAEL010215	2-3	18018	34	11551	1,89	64,11
AAEL010230	2-3	11258	35	8065	3,11	71,64
AAEL010240	1-2	8303	10	2299	1,20	27,69
AAEL010261	1-2	2125	5	569	2,35	26,78
AAEL010270	1-2	5598	8	3946	1,43	70,49
AAEL010282	1-2	67200	138	31243	2,05	46,49
AAEL010299	3-4	1681	2	113	1,19	6,72
AAEL010300	2-3	24729	48	12187	1,94	49,28
AAEL010315	2-3	11298	31	7372	2,74	65,25
AAEL010325	4-5	10922	21	4737	1,92	43,37
AAEL010333	1-2	4629	9	1453	1,94	31,39
AAEL010349	2-3	6176	19	2658	3,08	43,04
AAEL010361	2-3	15764	31	5920	1,97	37,55
AAEL010370	1-2	8469	23	3380	2,72	39,91
AAEL010389	2-3	9984	27	5359	2,70	53,68
AAEL010401	3-4	1373	3	433	2,18	31,54
AAEL010401	7-8	8817	23	5233	2,61	59,35
AAEL010423	2-3	11823	33	6667	2,79	56,39
AAEL010445	1-2	40997	92	12784	2,24	31,18
AAEL010445	2-3	10075	19	3631	1,89	36,04
AAEL010460	1-2	11138	20	8875	1,80	79,68
AAEL010480	1-2	10084	21	5666	2,08	56,19
AAEL010492	2-3	7387	14	1538	1,90	20,82
AAEL010502	1-2	9045	25	7630	2,76	84,36
AAEL010502	4-5	10339	21	6777	2,03	65,55
AAEL010523	2-3	1496	4	619	2,67	41,38
AAEL010530	2-3	15570	28	10523	1,80	67,59
AAEL010545	3-4	18245	47	8299	2,58	45,49
AAEL010560	1-2	11138	20	8875	1,80	79,68
AAEL010578	1-2	2530	6	811	2,37	32,06
AAEL010595	2-3	3034	10	1033	3,30	34,05
AAEL010595	1-2	6198	15	2016	2,42	32,53
AAEL010612	1-2	7521	21	3504	2,79	46,59
AAEL010635	1-2	7744	12	5394	1,55	69,65
AAEL010655	2-3	10906	25	2658	2,29	24,37
AAEL010672	2-3	30117	67	11276	2,22	37,44
AAEL010690	1-2	8469	26	4929	3,07	58,20
AAEL010713	1-2	2223	5	1122	2,25	50,47
AAEL010729	3-4	14911	33	5390	2,21	36,15
AAEL010740	2-3	8024	15	1506	1,87	18,77
AAEL010765	1-2	32590	71	19785	2,18	60,71
AAEL010781	1-2	3962	9	1006	2,27	25,39
AAEL010796	2-3	21804	30	14476	1,38	66,39
AAEL010813	1-2	8520	19	5818	2,23	68,29
AAEL010823	2-3	19843	41	8531	2,07	42,99
AAEL010834	2-3	10165	20	3681	1,97	36,21
AAEL010850	1-2	13315	28	4737	2,10	35,58
AAEL010850	4-5	6061	3	227	0,49	3,75
AAEL010860	3-4	14717	34	7088	2,31	48,16
AAEL010871	1-2	3520	12	1716	3,41	48,75

AAEL010871	2-3	7932	14	1262	1,77	15,91
AAEL010881	2-3	3030	7	1666	2,31	54,98
AAEL010890	6-7	11732	25	3838	2,13	32,71
AAEL010900	2-3	7857	15	4139	1,91	52,68
AAEL010922	1-2	1360	2	177	1,47	13,01
AAEL010930	1-2	5147	8	2739	1,55	53,22
AAEL010946	4-5	7904	20	4461	2,53	56,44
AAEL010961	2-3	7288	12	2911	1,65	39,94
AAEL010971	2-3	2417	6	1069	2,48	44,23
AAEL010985	1-2	1932	2	669	1,04	34,63
AAEL011003	1-2	7141	13	2955	1,82	41,38
AAEL011025	1-2	14842	33	6053	2,22	40,78
AAEL011050	1-2	7334	17	3320	2,32	45,27
AAEL011061	8-9	7259	23	3420	3,17	47,11
AAEL011061	10-11	4789	15	2280	3,13	47,61
AAEL011074	2-3	2223	5	715	2,25	32,16
AAEL011097	1-2	4996	17	1964	3,40	39,31
AAEL011119	2-3	4850	1	4368	0,21	90,06
AAEL011142	1-2	10677	26	3074	2,44	28,79
AAEL011165	1-2	2977	4	2123	1,34	71,31
AAEL011176	1-2	3337	9	1119	2,70	33,53
AAEL011191	1-2	10146	19	2304	1,87	22,71
AAEL011211	1-2	18378	44	9869	2,39	53,70
AAEL011235	2-3	21524	28	11791	1,30	54,78
AAEL011257	1-2	5774	11	3339	1,91	57,83
AAEL011276	1-2	6759	14	1269	2,07	18,77
AAEL011298	1-2	11400	31	4648	2,72	40,77
AAEL011319	1-2	8693	24	3600	2,76	41,41
AAEL011330	3-4	9850	23	3594	2,34	36,49
AAEL011345	2-3	5877	19	1677	3,23	28,53
AAEL011361	1-2	14751	22	8204	1,49	55,62
AAEL011371	1-2	4668	4	232	0,86	4,97
AAEL011398	1-2	5295	17	2004	3,21	37,85
AAEL011412	1-2	6095	11	1762	1,80	28,91
AAEL011434	1-2	2073	4	466	1,93	22,48
AAEL011447	1-2	1818	3	697	1,65	38,34
AAEL011447	4-5	7064	18	3404	2,55	48,19
AAEL011459	1-2	2671	9	886	3,37	33,17
AAEL011469	1-2	10360	27	4986	2,61	48,13
AAEL011479	6-7	7282	23	2879	3,16	39,54
AAEL011492	1-2	11871	20	7452	1,68	62,77
AAEL011513	6-7	18419	34	10974	1,85	59,58
AAEL011534	6-7	10522	14	8524	1,33	81,01
AAEL011556	1-2	7115	19	4064	2,67	57,12
AAEL011570	3-4	17375	37	10274	2,13	59,13
AAEL011583	2-3	11888	24	6169	2,02	51,89
AAEL011599	1-2	8029	13	7046	1,62	87,76
AAEL011612	1-2	41071	68	18607	1,66	45,30
AAEL011622	1-2	5699	17	2696	2,98	47,31
AAEL011638	3-4	14889	19	2935	1,28	19,71
AAEL011654	2-3	10411	24	3627	2,31	34,84
AAEL011654	3-4	12441	22	6856	1,77	55,11
AAEL011671	2-3	5303	13	1581	2,45	29,81
AAEL011689	2-3	12998	24	5479	1,85	42,15
AAEL0116701	2-3	9051	24	4631	2,65	51,17
AAEL011722	2-3	24410	45	17464	1,84	71,54
AAEL011745	1-2	12826	33	4782	2,57	37,28

AAEL011760	1-2	6395	14	5629	2,19	88,02
AAEL011779	1-2	20762	36	14929	1,73	71,91
AAEL011792	2-3	8062	23	5035	2,85	62,45
AAEL011800	2-3	17317	36	10229	2,08	59,07
AAEL011810	1-2	2850	7	1618	2,46	56,77
AAEL011834	2-3	25715	42	15294	1,63	59,48
AAEL011857	1-2	3158	9	2537	2,85	80,34
AAEL011878	3-4	5787	16	3373	2,76	58,29
AAEL011899	2-3	14315	35	6833	2,44	47,73
AAEL011922	1-2	4187	7	813	1,67	19,42
AAEL011945	1-2	9728	21	4232	2,16	43,50
AAEL011969	1-2	15609	25	6149	1,60	39,39
AAEL011969	2-3	8860	11	6212	1,24	70,11
AAEL011994	1-2	16236	41	7995	2,53	49,24
AAEL012018	1-2	9213	30	5215	3,26	56,60
AAEL012048	1-2	17854	48	6807	2,69	38,13
AAEL012076	3-4	11645	29	5421	2,49	46,55
AAEL012091	3-4	10187	15	2326	1,47	22,83
AAEL012091	4-5	8600	27	3940	3,14	45,81
AAEL012105	4-5	23399	9	1029	0,38	4,40
AAEL012130	1-2	14295	49	5833	3,43	40,80
AAEL012151	3-4	3468	7	3204	2,02	92,39
AAEL012178	2-3	5694	12	4552	2,11	79,94
AAEL012203	1-2	25280	57	8478	2,25	33,54
AAEL012231	1-2	3186	1	3186	0,31	100,00
AAEL012048	1-2	17854	48	6807	2,69	38,13
AAEL012062	3-4	11169	20	2601	1,79	23,29
AAEL012279	2-3	2376	8	1485	3,37	62,50
AAEL012290	1-2	7971	16	3231	2,01	40,53
AAEL012290	3-4	9037	16	2380	1,77	26,34
AAEL012310	2-3	16593	34	5487	2,05	33,07
AAEL012329	1-2	1749	5	873	2,86	49,91
AAEL012354	2-3	4892	11	2327	2,25	47,57
AAEL012370	1-2	11347	25	5573	2,20	49,11
AAEL012396	1-2	6342	14	2953	2,21	46,56
AAEL012420	3-4	8504	19	3245	2,23	38,16
AAEL012440	1-2	20381	34	11782	1,67	57,81
AAEL012440	2-3	13392	34	5127	2,54	38,28
AAEL012451	1-2	3941	9	1047	2,28	26,57
AAEL012465	2-3	1356	5	588	3,69	43,36
AAEL012482	1-2	1147	5	870	4,36	75,85
AAEL012500	3-4	9935	28	6509	2,82	65,52
AAEL012524	1-2	9837	24	3690	2,44	37,51
AAEL012532	2-3	4865	15	2078	3,08	42,71
AAEL012549	1-2	23451	54	13144	2,30	56,05
AAEL012467	1-2	19183	29	13318	1,51	69,43
AAEL012467	3-4	13151	40	6911	3,04	52,55
AAEL012604	1-2	38542	86	14709	2,23	38,16
AAEL012620	1-2	37079	73	14619	1,97	39,43
AAEL012640	2-3	11636	28	4656	2,41	40,01
AAEL012662	1-2	5539	16	2226	2,89	40,19
AAEL012662	2-3	6482	17	2994	2,62	46,19
AAEL012684	2-3	6177	19	4200	3,08	67,99
AAEL012699	1-2	9147	18	2743	1,97	29,99
AAEL012714	1-2	5260	11	4867	2,09	92,53
AAEL012730	8-9	20006	40	11082	2,00	55,39
AAEL012749	1-2	8107	13	5676	1,60	70,01

AAEL012761	2-3	7971	16	4147	2,01	52,03
AAEL012784	1-2	2521	6	756	2,38	29,99
AAEL012802	2-3	4474	13	1765	2,91	39,45
AAEL012820	3-4	9039	22	4082	2,43	45,16
AAEL012844	3-4	13869	27	6000	1,95	43,26
AAEL012860	1-2	18110	34	10037	1,88	55,42
AAEL012881	2-3	3833	13	1530	3,39	39,92
AAEL012881	3-4	3815	9	654	2,36	17,14
AAEL012992	1-2	3721	14	1787	3,76	48,02
AAEL013015	1-2	25296	52	10005	2,06	39,55
AAEL013034	3-4	4766	7	2832	1,47	59,42
AAEL013034	4-5	18655	28	13950	1,50	74,78
AAEL013049	1-2	9598	18	2904	1,88	30,26
AAEL013059	2-3	18158	39	8581	2,15	47,26
AAEL013004	4-5	1760	4	925	2,27	52,56
AAEL013025	2-3	11672	22	6377	1,88	54,64
AAEL013050	1-2	8756	24	4883	2,74	55,77
AAEL013071	1-2	11003	23	7039	2,09	63,97
AAEL013092	2-3	3521	10	1559	2,84	44,28
AAEL013109	1-2	2494	5	1976	2,00	79,23
AAEL013128	2-3	40075	76	13219	1,90	32,99
AAEL013145	1-2	5155	11	1870	2,13	36,28
AAEL013172	1-2	13352	25	6440	1,87	48,23
AAEL013199	4-5	4056	7	2933	1,73	72,31
AAEL013219	3-4	7507	9	1534	1,20	20,43
AAEL013219	6-7	11138	17	3693	1,53	33,16
AAEL013230	4-5	7117	16	2115	2,25	29,72
AAEL013251	2-3	17707	29	11526	1,64	65,09
AAEL013274	2-3	10534	21	3564	1,99	33,83
AAEL013297	1-2	1445	2	106	1,38	7,34
AAEL013313	1-2	7237	10	4102	1,38	56,68
AAEL013333	2-3	14215	30	9360	2,11	65,85
AAEL013359	1-2	12741	30	4053	2,35	31,81
AAEL013378	4-5	15679	40	9312	2,55	59,39
AAEL013378	5-6	10079	21	4147	2,08	41,14
AAEL013398	4-5	12680	26	8411	2,05	66,33
AAEL013425	2-3	41048	93	19711	2,27	48,02
AAEL013441	1-2	25962	57	15625	2,20	60,18
AAEL013464	2-3	5542	9	3971	1,62	71,65
AAEL013482	2-3	15020	32	4349	2,13	28,95
AAEL013483	1-2	8807	10	6859	1,14	77,88
AAEL013501	3-4	7060	18	5518	2,55	78,16
AAEL013521	2-3	6060	14	2200	2,31	36,30
AAEL013530	1-2	5578	13	1649	2,33	29,56
AAEL013530	2-3	55054	42	8688	0,76	15,78
AALE013542	2-3	11431	30	4124	2,62	36,08
AAEL013559	8-9	2964	2	149	0,67	5,03
AAEL013574	4-5	12165	20	2731	1,64	22,45
AAEL013592	1-2	10822	20	9580	1,85	88,52
AAEL013592	2-3	6916	12	3227	1,74	46,66
AAEL013610	1-2	4099	7	2421	1,71	59,06
AAEL013625	3-4	14475	33	4761	2,28	32,89
AAEL013644	6-7	6840	12	2078	1,75	30,38
AAEL013662	3-4	13903	28	5107	2,01	36,73
AAEL013683	1-2	14528	32	6402	2,20	44,07
AAEL013700	1-2	16004	36	8301	2,25	51,87
AAEL013729	1-2	5219	14	1488	2,68	28,51

AAEL013746	3-4	2498	9	748	3,60	29,94
AAEL013761	1-2	49073	92	21259	1,87	43,32
AAEL013770	1-2	7050	22	3416	3,12	48,45
AAEL013783	3-4	17369	35	6078	2,02	34,99
AAEL013799	1-2	13400	29	7803	2,16	58,23
AAEL013811	4-5	8828	23	5172	2,61	58,59
AAEL013829	1-2	43545	73	12782	1,68	29,35
AAEL013841	1-2	17216	34	12268	1,97	71,26
AAEL013860	1-2	16091	22	9879	1,37	61,39
AAEL013880	3-4	17966	28	10630	1,56	59,17
AALE013899	1-2	7432	12	1723	1,61	23,18
AAEL013921	1-2	1873	4	940	2,14	50,19
AAEL013941	1-2	5103	23	2755	4,51	53,99
AAEL013959	1-2	1149	2	482	1,74	41,95
AAEL013978	1-2	17401	39	8602	2,24	49,43
AAEL013995	2-3	9269	21	7546	2,27	81,41
AAEL014010	1-2	22231	43	11406	1,93	51,31
AAEL014034	1-2	18366	34	9503	1,85	51,74
AAEL014034	2-3	5737	9	1409	1,57	24,56
AAEL014054	2-3	9921	26	4821	2,62	48,59
AAEL014071	3-.4	3557	8	791	2,25	22,24
AAEL014098	2-3	18201	23	11707	1,26	64,32
AAEL014109	1-2	5522	13	2837	2,35	51,38
AAEL014129	2-3	5023	9	1158	1,79	23,05
AAEL014149	2-3	2953	6	955	2,03	32,34
AAEL014165	2-3	13913	34	5934	2,44	42,65
AAEL014177	3-4	20629	44	11927	2,13	57,82
AAEL014189	1-2	1729	3	507	1,74	29,32
AAEL014200	1-2	3179	5	1677	1,57	52,75
AAEL014221	2-3	6601	20	4102	3,03	62,14
AAEL014243	1-2	11968	33	5510	2,76	46,04
AAEL014265	1-2	9257	20	6982	2,16	75,42
AAEL014286	1-2	9370	28	3589	2,99	38,30
AAEL014301	5-6	2718	8	1558	2,94	57,32
AAEL014332	2-3	25539	62	9470	2,43	37,08
AAEL014354	2-3	1687	4	667	2,37	39,54
AAEL014365	4-5	6699	19	4299	2,84	64,17
AAEL014379	1-2	14804	32	3540	2,16	23,91
AAEL014395	5-6	15088	35	6631	2,32	43,95
AAEL014419	1-2	4979	13	2123	2,61	42,64
AAEL014436	4-5	4341	10	2203	2,30	50,75
AAEL014445	1-2	19423	44	8042	2,27	41,40
AAEL014450	1-2	5776	12	3417	2,08	59,16
AAEL014456	1-2	25894	48	12959	1,85	50,05
AAEL014489	1-2	4921	17	2243	3,45	45,58
AAEL014511	1-2	5405	14	2233	2,59	41,31
AAEL014520	2-3	7094	20	5355	2,82	75,49
AAEL014530	1-2	11285	30	6424	2,66	56,93
AAEL014545	1-2	2204	9	1605	4,08	72,82
AAEL014545	2-3	6317	8	3378	1,27	53,47
AAEL014558	4-5	28896	67	17058	2,32	59,03
AAEL014571	1-2	10028	18	2493	1,79	24,86
AAEL014581	1-2	5042	8	1483	1,59	29,41
AAEL014599	1-2	6734	13	2299	1,93	34,14
AAEL014627	4-5	8005	23	3845	2,87	48,03
AAEL014637	2-3	5095	13	2075	2,55	40,73
AAEL014653	7-8	21200	34	10865	1,60	51,25

AAEL014666	3-4	15062	28	5454	1,86	36,21
AAEL014682	1-2	4257	10	1823	2,35	42,82
AAEL014694	2-3	19150	49	5778	2,56	30,17
AAEL014801	2-3	11865	20	2584	1,69	21,78
AAEL014820	2-3	1595	3	651	1,88	40,82
AAEL014845	1-2	4259	9	2593	2,11	60,88
AAEL014871	1-2	9776	17	4725	1,74	48,33
AAEL014895	1-2	1112	2	162	1,80	14,57
AAEL014913	2-3	9816	15	3353	1,53	34,16
AAEL014931	1-3	13032	32	4135	2,46	31,73
AAEL014943	3-4	9227	11	1120	1,19	12,14
AAEL014963	3-4	6810	22	3790	3,23	55,65
AAEL014980	1-2	4446	5	3636	1,12	81,78
AAEL014998	4-5	6714	15	2374	2,23	35,36
AAEL015013	1-2	16654	34	10411	2,04	62,51
AAEL015039	3-4	10923	41	7045	3,75	64,50
AAEL015056	7-8	5381	13	2205	2,42	40,98
AAEL015080	1-2	10159	18	4450	1,77	43,80
AAEL015100	1-2	8125	24	4335	2,95	53,35
AAEL015110	4-5	4492	1	4421	0,22	98,42
AAEL015130	5-6	17978	38	7311	2,11	40,67
AAEL015142	2-3	4234	10	1470	2,36	34,72
AAEL015156	1-2	11846	29	5386	2,45	45,47
AAEL015170	2-3	1467	3	285	2,04	19,43
AAEL015111	1-2	4741	13	1949	2,74	41,11
AAEL015120	2-3	7054	23	3063	3,26	43,42
AAEL015145	1-2	11754	27	5272	2,30	44,85
AAEL015161	1-2	4611	9	1472	1,95	31,92
AAEL015170	2-3	1467	3	285	2,04	19,43
AAEL015202	1-2	3953	8	629	2,02	15,91
AAEL015235	2-3	5043	9	2719	1,78	53,92
AAEL015251	2-3	8140	12	2235	1,47	27,46
AAEL015271	3-4	9278	25	3640	2,69	39,23
AAEL015291	1-2	6197	12	2036	1,94	32,85
AAEL015314	1-2	7739	17	5027	2,20	64,96
AAEL015352	5-6	1012	1	636	0,99	62,85
AAEL015399	1-2	1775	7	565	3,94	31,83
AAEL015425	2-3	4855	16	2842	3,30	58,54
AAEL015466	1-2	4724	11	1227	2,33	25,97
AAEL015512	5-6	1763	4	247	2,27	14,01
AAEL015575	1-2	2769	7	702	2,53	25,35
