# Supplementary material for: The Orthologue of the Fruitfly Sex Behaviour Gene Fruitless in the Mosquito Aedes aegypti: Evolution of Genomic Organisation and Alternative Splicing
Source: PLoS One. 2013 Feb 13;8(2):e48554. doi: 10.1371/journal.pone.0048554 (PMC3572092; doi:10.1371/journal.pone.0048554)
Supplement: Table S3 — Regulatory elements in the sex-specifically regulated region of fru homologs. Sequence of the putative Aeafru cis identified in the female-specific exon P1-f. The upper case indicate conserved nucleotides respect to the consensus sequences of Drosophila. The distance of these elements from the male- and female-specific 5′ splicing donor sites are indicated. (PDF) [file pone.0048554.s010.pdf]

Table S3 - Regulatory elements in the sex-specifically regulated region of *fru* homologs

Element	Element sequence	Identity	bp to male-specific 5'splicing donor	bp to female-specific 5'splicing donor
**Dmdsx TRA/TRA-2 b.s.**	**TCTTCAATCAACAAA**			
Dmfru TRA/TRA-2 b.s. 1	TCATCAATCAACA	13/13	1352	238
Dmfru TRA/TRA-2 b.s. 2	TCTTCAATCAACA	13/13	1387	203
Dmfru TRA/TRA-2 b.s. 3	aCTTCAATCAACA	12/13	1540	50
Angfru TRA/TRA-2 b.s. 1	TaAACAAaCAAac	9/13	1439	311
Angfru TRA/TRA-2 b.s. 2	gCTTCAATCAAac	10/13	1503	247
Angfru TRA/TRA-2 b.s. 3	ctATCAAcCAAac	8/13	1524	226
Aeafru TRA/TRA-2 b.s. 1	TCATCAATCtAct	10/13	1091	265
Aeafru TRA/TRA-2 b.s. 2	gCAcCtgTCAACA	9/13	1148	208
Aeafru TRA/TRA-2 b.s. 3	cCcTCAATCAgCA	10/13	1205	151
**Dm TRA-2-ISS**	**CAAGGA**			
Aeafru TRA-2-ISS 1	CAAGG	5/5	153	1137
Aeafru TRA-2-ISS 2	CAAGG	5/5	485	805
Aeafru TRA-2-ISS 3	CAAGA	5/5	728	562
**Dm RBP1-Type B**	**ATCCNNAT**			
Aeafru RBP1-Type B 1	ATCTGAA	7/7	351	1646
Aeafru RBP1-Type B 2	ATCTAGA	7/7	266	1561
Aeafru RBP1-Type B 3	ATCCCCA	7/7	61	1356
Aeafru RBP1-Type B 4	ATCTTTA	7/7	454	834
Aeafru RBP1-Type B 5	ATCCGTA	7/7	743	545
Aeafru RBP1-Type B 6	ATCTAAA	7/7	968	320
Aeafru RBP1-Type B 7	ATCCGAA	7/7	1025	263
Aeafru RBP1-Type B 8	ATCCGAA	7/7	1068	220
Aeafru RBP1-Type B 9	ATCCGAA	7/7	1109	179

In lower case nucleotides not matching with the consensus
