# Supplementary material for: The Orthologue of the Fruitfly Sex Behaviour Gene Fruitless in the Mosquito Aedes aegypti: Evolution of Genomic Organisation and Alternative Splicing
Source: PLoS One. 2013 Feb 13;8(2):e48554. doi: 10.1371/journal.pone.0048554 (PMC3572092; doi:10.1371/journal.pone.0048554)
Supplement: Table S4 — Correspondence between MEME identified motifs and motif from RegRNA database. The nucleotides of MEME motifs corresponding to RegRNA motifs areshaded in light grey. (PDF) [file pone.0048554.s011.pdf]

Table S4 - Correspondence between MEME identified motifs and motif from RegRNA database.

RegRNA Acc. number	RegRNA motif (consensus)	Motif class	Footprinter motif (in grey the RegRNA motif)	Corresponding gene	Binding Protein	Drosophila homolog
**R0928**	AAGT	Exonic splicing enhancer	Motif 18 GCAAGTTCT<br>Motif 18 GCAAGTTCA	*Aeadsx*<br>*Aeafru*	hnRNPG	Hrb27C (CG10377)
**R0612**	GRYYMCYR	Exonic splicing enhancer	Motif 13 GGCCACTGT	*Aeafru*	---	SC35 (CG5442)
**R0915**	TGGT	Exonic splicing silencer	Motif 17 CGTTGGTAT<br>Motif 21 GTGGTACTT<br>Motif 26 CATGGTGT<br>Motif 26 CATGGTGG	*Aeafru*<br>*Aeafru*<br>*Aeadsx*<br>*Aeafru*	Unknown	---
**R0621**	YRCRKM	Exonic splicing enhancer	Motif 5 CGATCGCGTC	*Aeafru*	SRp40	B52 (CG10851)
**R1033**	YCAY	Intronic splicing enhancer	Motif 9 GCAGCCATTC<br>Motif 13 CGCCACTGT<br>Motif 13 GGCCACTGT	*Aeadsx*<br>*Aeafru*<br>*Aeafru*	Nova-1	Pasilla (CG42670)
**R0987**	CTGN	Intronic splicing enhancer	Motif 10 GAGCCTGAAT<br>Motif 13 CGCCACTGT<br>Motif 13 GGCCACTGT<br>Motif 22 ATCTGAAA<br>Motif 35 ACTGTTCT<br>Motif 35 ACTGTTCT	*Aeadsx*<br>*Aeafru*<br>*Aeafru*<br>*Aeafru*<br>*Aeadsx*<br>*Aeafru*	Unknown	---
**R0952**	WGGG	Intronic splicing enhancer	Motif 17 CGTGGGTAT	*Aeadsx*	Unknown	---
**R0805**	CATGG	Exonic splicing silencer	Motif 26 CATGGTGT<br>Motif 26 CATGGTGG	*Aeadsx*<br>*Aeafru*	Unknown	---
**R601**	WCWWC	Exonic splicing enhancer	Motif 1 AACATCCATCCGAAA<br>Motif 1 AACAACCATCCGAAA<br>Motif 8 TCTACTTGTG<br>Motif 8 TCTACTAGTG<br>Motif 20 TGTCTTCCA	*Aeadsx*<br>*Aeafru*<br>*Aeadsx*<br>*Aeafru*<br>*Aeafru*	SRp20	x16 (CG10203)

Shaded in light grey the nucleotides of MEME motifs corresponding to RegRNA motifs

Y= C/T
R= A/G
K= G/T
M= A/C
W= A/T
S= C/G
